# Discovery of Highly Selective Noncovalent Macrocyclic Peptide Inhibitors Suppressing Both TMPRSS2 Protease Activity and Viral Receptor Function

**DOI:** 10.1021/acs.jmedchem.6c01094

**Published:** 2026-08-12

**Authors:** Benjamin J. Tombling, Gabriel Lemieux, Alexandre Joushomme, Yuehua Wei, Emel Adaligil, Brennan Farrell, Steven Fleming, Qinying Yu, Bin Ma, Christian N. Cunningham, Robin Krystufek, Aimin Song, Antoine Désilets, Richard Leduc, Daniel Kirchhofer

**Affiliations:** † Department of Early Discovery Biochemistry, Genentech Inc., 1 DNA Way, South San Francisco, California 94080, United States; ‡ Department of Pharmacology-Physiology, Faculty of Medicine and Health Sciences, Institut de Pharmacologie de Sherbrooke, Université de Sherbrooke, Sherbrooke, Québec J1H5H3, Canada; § Department of Drug Metabolism & Pharmacokinetics, Genentech Inc., 1 DNA Way, South San Francisco, California 94080, United States

## Abstract

The type II transmembrane serine protease TMPRSS2 plays a critical role in respiratory virus entry, including SARS-CoV-2. Using an mRNA display platform with genetic code reprogramming, we identified macrocyclic peptide (MCP) inhibitors of TMPRSS2 that bound to the TMPRSS2 active site. The most potent MCPs displayed picomolar binding affinities and excellent selectivity across a panel of 22 trypsin-fold serine proteases. Although the lead MCP showed strong *in vitro* potency, proteolytic instability reduced its cellular activity. Rational optimization yielded a stability-enhanced variant, T2-MCP-19, which bound TMPRSS2 with high affinity (*K*_D_ = 80 pM) and inhibited the uptake of virus-like particles pseudotyped with SARS-CoV-2 spike protein in lung epithelial Calu-3 cells with nanomolar potency. Notably, several MCPs exhibited dual functionality by inhibiting both the enzymatic and receptor functions of TMPRSS2, as demonstrated by their blockade of the HKU1 coronavirus spike protein binding. These results highlight MCPs as promising, highly selective TMPRSS2-directed antiviral lead compounds.

## Graphical abstract

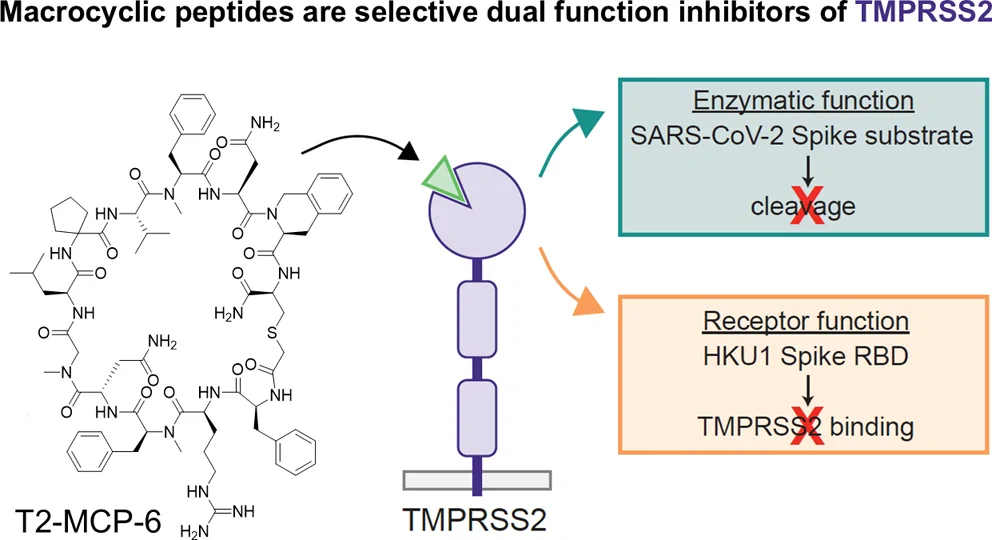

## Introduction

The type II transmembrane serine proteases (TTSPs) are a family of membrane-anchored enzymes that perform a broad range of biological functions and are implicated in numerous disease pathways.^1–3^ One of the most prominent TTSPs is the transmembrane serine protease 2 (TMPRSS2), which plays a major role in respiratory viral pathogenesis, including SARS-CoV-2 and influenza.^4–9^ The extracellular domain of TMPRSS2 is composed of an LDL receptor type A (LDLR-A) domain, a scavenger receptor cysteine-rich (SRCR) domain, and a C-terminal trypsin-fold protease domain.^10^ The synthesized inactive zymogen undergoes autocatalytic cleavage at the R255-I256 bond between the disulfide-bonded SRCR and protease domains to form the enzymatically active membrane-bound protease.^11^ TMPRSS2 predominantly promotes viral pathogenesis by priming viral surface glycoproteins to enable host cell entry.^12^ While TMPRSS2 was reported to cleave the SARS-CoV-2 spike protein at both S1/S2 and S2′ sites,^13,14^ its primary role in viral entry is mainly associated with S2′ cleavage, which occurs after the polybasic S1/S2 site has been processed by furin. TMPRSS2 cleavage of the spike protein at the S2′ leads to the exposure of the viral fusion peptide that triggers membrane fusion with host cells.^4,8,15^ Although additional cell surface and endosomal proteases have been shown to cleave the SARS-CoV-2 spike protein for viral entry,^16–19^ TMPRSS2^–/–^ knockout mouse models have revealed the essential contribution of TMPRSS2 to the viral infection process from evolving SARS-CoV-2 variants of concern (VOCs).^20,21^ In addition to enzymatically priming viral surface glycoproteins, TMPRSS2 was recently shown to promote viral entry of the human coronavirus strain HKU1 in a nonproteolytic manner by the usage of its catalytic domain as a receptor for the HKU1 spike protein.^22–25^ This dual functionality establishes an intriguing new paradigm within the TTSP family, where TMPRSS2 serves as both a conventional protease and a surface receptor.

Because of its important function in viral entry, TMPRSS2 has attracted strong interest as a promising therapeutic target. A key advantage of host-directed antiviral strategies, such as targeting TMPRSS2, is the reduced propensity for resistance driven by target mutations, which is commonly observed with virus-directed SARS-CoV-2 therapies.^18,26–30^ To date, efforts to develop TMPRSS2 inhibitors have largely centered on repurposing antiviral small molecules or developing short peptidomimetics.^4,31–39^ Two small molecules, camostat mesylate and nafamostat, were investigated in clinical trials for preventing disease severity in patients with SARS-CoV-2. However, neither molecule met their primary end points,^40–42^ potentially due to pharmacokinetic limitations. N-0385, a peptidomimetic with a ketobenzothiazole-based covalent warhead, showed potent TMPRSS2 inhibition and *in vivo* antiviral efficacy against the SARS-CoV-2 USA-WA1/2020 reference strain and the Delta VOC (B.1.617.2).^31^ Comparable *in cellulo* antiviral efficacy of this compound was also reported across pre-Omicron and Omicron variants.^31,36,43^ However, a common drawback to these TMPRSS2 inhibitors is their lack of selectivity, which is potentially due to their substrate-like binding and covalent mechanisms of action. Beyond covalent inhibitors, several nanobodies were recently identified that inhibit TMPRSS2 enzymatic activity and receptor function with moderate potency; however, to our knowledge, their selectivity profiles have not been reported.^22,24,44^

Macrocyclic peptides (MCPs) have gained interest as an attractive therapeutic modality due to their ability to target challenging protein interfaces with high potency, affinity, and selectivity in a noncovalent manner.^45,46^ mRNA display has become a powerful technology for identifying potent MCPs with drug-like properties from highly diverse peptide libraries (up to the 10^13^ unique peptides) through genetic code reprogramming with non-natural amino acids. The incorporation of non-natural amino acids into the peptide libraries significantly increases the chemical diversity and proteolytic stability of identified MCPs.^47^ mRNA display with genetic code reprogramming technology has enabled the successful identification of MCPs against protease targets, such as FXIIa^48,49^ and Zika virus NS2B-NS3 protease.^50^ Of note, MCPs discovered from mRNA display have been identified to interfere with SARS-CoV-2 entry, such as inhibitors of the SARS-CoV-2 spike protein binding to ACE2,^51,52^ and inhibitors of the SARS-CoV-2 main protease.^53,54^

In this study, we utilized an mRNA display platform to identify MCPs with strong inhibitory activity against TMPRSS2. Biochemical studies revealed that these MCPs bound to the TMPRSS2 active site and displayed exquisite selectivity over related serine proteases. Notably, some MCPs also effectively disrupted TMPRSS2 viral receptor function, which involves its active-site region. Engineering of the lead MCP yielded several derivatives with significantly enhanced proteolytic stability and improved binding affinities, which translated into potent inhibition of pericellular TMPRSS2 activity and prevented entry of virus-like particles pseudotyped with the SARS-CoV-2 spike protein. Given their high potency and remarkable selectivity, the identified MCPs are promising candidates for further development as antiviral agents and valuable tools for investigating TMPRSS2 function in viral and nonviral biological pathways.

## Results

### *In Vitro* Selection of Macrocyclic Peptides Targeting Engineered TMPRSS2 Using mRNA Display

The recombinant expression and purification of TTSP family members, including TMPRSS2, have proven to be challenging.^14,55^ A successful strategy reported involves replacement of the autocatalytic activation sequence of TTSPs with an enterokinase cleavage tag (DDDDK) to control activation and enhancing expression.^13,56–58^ We applied this approach to the mammalian cell expression of a soluble form of the extracellular domain of human TMPRSS2 (amino acids M109–V492) with an inserted enterokinase cleavage sequence (DDDDK) replacing the native activation sequence (^251^SRQSR^255^; see Figure 1a). Following expression and purification of the single-chain zymogen, we activated the protein using enterokinase and confirmed that the engineered recombinant human TMPRSS2 (subsequently referred to as TMPRSS2) formed a disulfide-linked heterodimeric protein (Figure S1a) and was enzymatically active toward the fluorogenic substrate Boc-QAR-AMC (Figure S1b).

**Figure 1:**
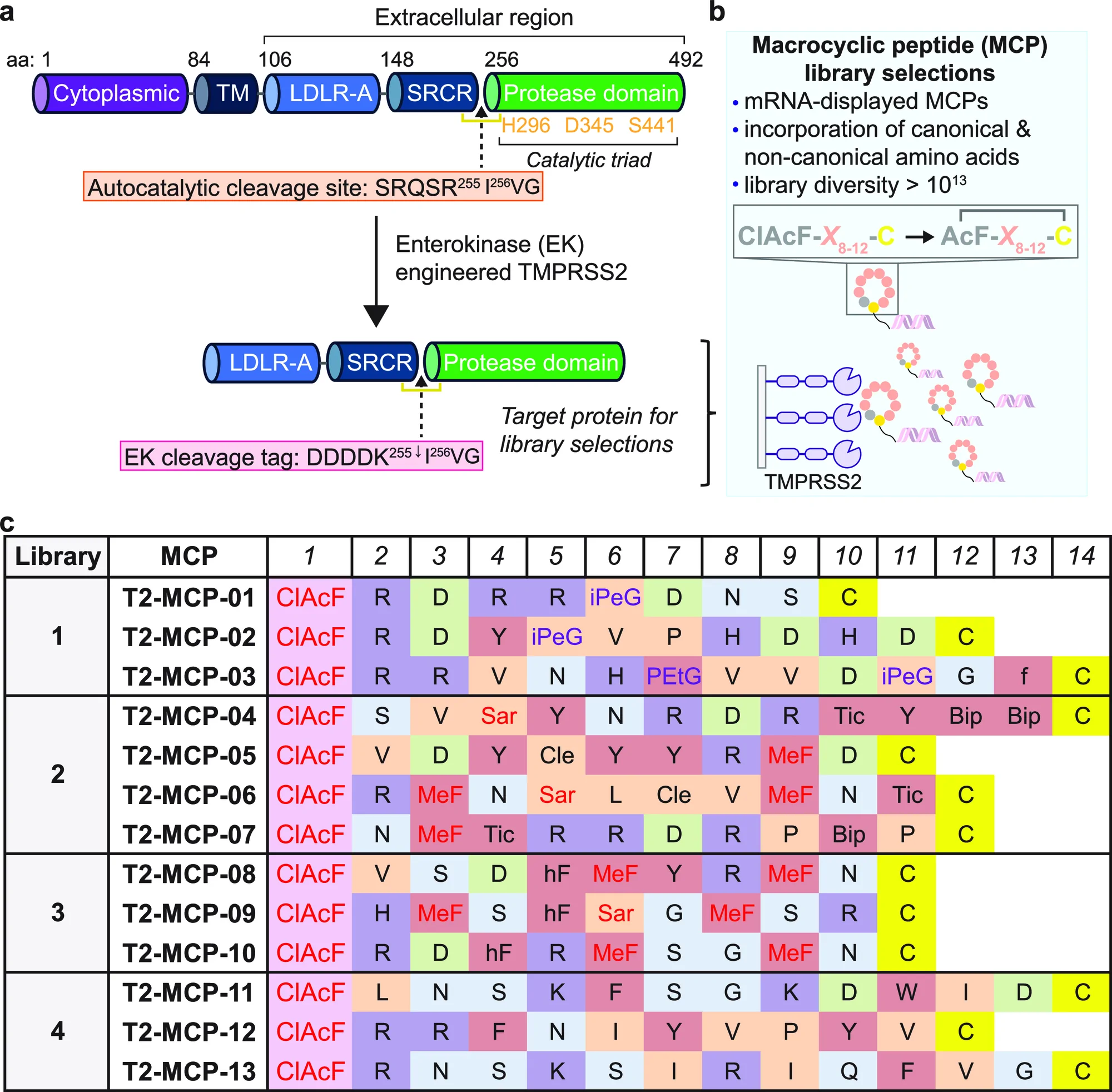
Discovery of macrocyclic peptide binders against recombinant TMPRSS2 using mRNA display. (a) Schematic representation of the protein construct design of engineered human TMPRSS2 used for library selections: TM, transmembrane domain; LDLR-A, low-density lipoprotein receptor class A domain; SRCR, scavenger receptor cysteine-rich domain. The autocatalytic cleavage site sequence of TMPRSS2 was replaced with an enterokinase (EK) cleavage tag (DDDDK^↓^) to allow EK-mediated activation of the engineered zymogen to form the mature protease. Amino acid numbering and domain boundaries are derived from Uniprot ID O15393. (b) mRNA display platform was used to perform affinity selections of macrocyclic peptide (MCP) libraries against immobilized TMPRSS2 on neutravidin-coated magnetic beads. MCP libraries containing eight to 12 randomized amino acids are diversified by incorporation of noncanonical amino acids through genetic code reprogramming, and they were cyclized by a thioether bond between the *N*-terminal chloroacetyl-l-phenylalanine (ClAcF) and the *C*-terminal Cys. (c) Selected enriched MCPs from affinity selections for further chemical synthesis based on their library source (see Figure S2 for library designs). Residues are color-coded according to physicochemical properties: cysteine (C), yellow; negatively charged (D), green; positively charged (R, K, H), purple; small/polar (G, S, N, Q), light blue; hydrophobic (V, L, I, P, Sar, Cle, iPeG), orange; and aromatic (F, W, Y, Tic, Bip, f, hF, MeF, PEtG), red. *N*-methylated amino acids (MeF, Sar) are colored as red text. Peptoid amino acids (iPeG, PEtG) are colored as text. The *N*-terminal chloroacetyl-l-phenylalanine (ClAcF) initiator is colored pink.

*In vitro* expressed MCP libraries were generated through genetic code reprogramming using a “flexible *in vitro* translation,” incorporating natural and non-natural amino acids (Figures 1b and S2). The four mRNA display libraries (Figure S2a,b) comprised 10–14-membered cyclic peptides that spontaneously cyclized after *in vitro* translation via an N-terminal chloroacetyl-l-phenylalanine (ClAcF) and a C-terminal cysteine, forming a thioether bond. After five rounds of affinity selections against biotinylated activated recombinant TMPRSS2, the enriched MCPs were identified using next-generation sequencing. Based on enrichment levels of peptide sequences (Table S1), library origin, and sequence diversity, 13 MCPs from all four mRNA display screenings were selected for solid-phase peptide synthesis and for further activity characterization (Figure 1c).

### Macrocyclic Peptides Bind to the TMPRSS2 Active Site and Potently Inhibit Enzyme Activity

All synthesized MCPs showed inhibitory activity toward TMPRSS2 in enzyme assays using the fluorescent substrate Boc-QAR-AMC (Table 1). Six of the MCPs, representing each of the four tested libraries, had IC_50_ values <10 nM, and inhibition curves showed these MCPs achieved complete enzyme inhibition (Figure 2a). The most potent MCP, T2-MCP-09, had an IC_50_ of 0.85 ± 0.08 nM, which was 4-fold more potent than camostat mesylate (IC_50_ of 3.6 ± 0.8 nM) in the same substrate cleavage assay (Figure 2a and Table 1). The more potent MCPs also strongly inhibited recombinant murine TMPRSS2 (amino acids W105–S490), designed as an EK-activatable protease just like human TMPRSS2 (see Figure S1c for enzymatic activity using the fluorogenic substrate Boc-QAR-AMC), suggesting the MCPs may display broad cross-species activity (Figure 2b and Table 1).

**Figure 2:**
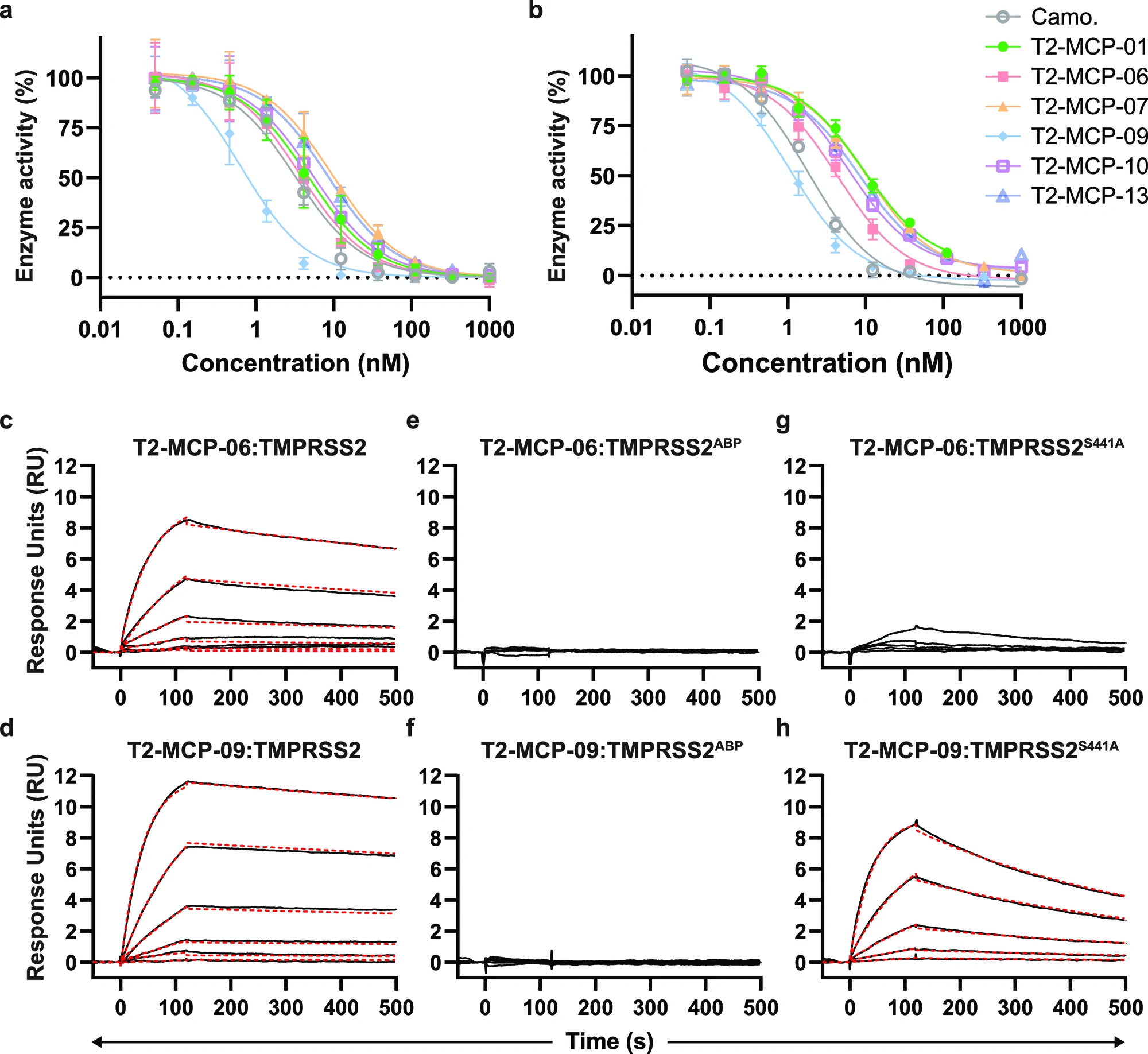
MCPs have potent inhibitory activities and strong binding affinities for recombinant TMPRSS2. (a, b) Selected MCPs fully inhibit the enzymatic activity of huTMPRSS2 (a) and muTMPRSS2 (b) toward the fluorogenic substrate Boc-QAR-AMC. IC_50_ values were calculated by generating a nonlinear regression with variable slope (four parameters) analysis from a log­[compound] versus normalized fluorescence data plot. Camostat mesylate (Camo.) was included as a control inhibitor. Data shows the average ± SD of three independent experiments. (c–h) Representative SPR sensorgrams showing concentration-dependent binding of T2-MCP-06 (1–300 nM) and T2-MCP-09 (0.2–50 nM) to immobilized huTMPRSS2 (c, d), to huTMPRSS2 with a covalently bound activity-based probe (TMPRSS2^ABP^) (e, f), and to huTMPRSS2 with its catalytic Ser mutated to Ala (TMPRSS2^S441A^) (g, h). Experimental curves are shown in black. Kinetic binding fits, based on a 1:1 model, are shown in red. Sensorgrams are representative of three independent experiments.

**Table 1:** Inhibitory Activities and Binding Affinities of mRNA Display-Derived MCPs against Recombinant TMPRSS2^a^

			TMPRSS2				TMPRSS2^S441A^			
MCP	huTMPRSS2 IC_50_ (nM)	muTMPRSS2 IC_50_ (nM)	*k*_on_ (1 × 10^5^ M^–1^s^–1^)	*k*_off_ (1 × 10^–3^ s^–1^)	*t*_1/2_ (min)	*K*_D_ (nM)	*k*_on_ (1 × 10^5^ M^–1^s^–1^)	*k*_off_ (1 × 10^–3^ s^–1^)	*t*_1/2_ (min)	*K*_D_ (nM)
**Camo**	3.6 ± 0.8	2.0 ± 0.1	N.T.	N.T.	N.T.	N.T.	N.T.	N.T.		N.T.
**T2-MCP-01**	3.4 ± 0.3	4.2 ± 1.2	4.3 ± 0.2	1.6 ± 0.3	7.3 ± 1.4	3.8 ± 0.8	1.4 ± 0.2	2.2 ± 0.19	5.3 ± 0.5	15 ± 2
**T2-MCP-02**	126 ± 8	195 ± 80	0.29 ± 0.01	1.4 ± 0.2	8.2 ± 1.0	50 ± 7	0.17 ± 0.03	1.0 ± 0.096	11.3 ± 1.1	64 ± 16
**T2-MCP-03**	111 ± 8	424 ± 69	0.88 ± 0.04	13 ± 3	0.9 ± 0.2	140 ± 30				
**T2-MCP-04**	22.2 ± 2.2	24.9 ± 5.7	0.22 ± 0.03	1.4 ± 0.2	8.6 ± 1.2	61 ± 8				
**T2-MCP-05**	31.7 ± 2.5	44.4 ± 8.5	0.22 ± 0.01	0.47 ± 0.04	24.9 ± 2.3	22 ± 3				
**T2-MCP-06**	3.9 ± 0.5	5.0 ± 1.6	1.3 ± 0.2	0.37 ± 0.02	31.2 ± 1.4	2.8 ± 0.2				
**T2-MCP-07**	9.8 ± 1.0	10.4 ± 0.9	2.7 ± 0.3	2.0 ± 0.03	5.7 ± 0.1	7.6 ± 0.8				
**T2-MCP-08**	18.7 ± 1.0	55.7 ± 14.1	0.24 ± 0.07	0.67 ± 0.02	17.2 ± 0.5	29 ± 10				
**T2-MCP-09**	0.85 ± 0.08	1.3 ± 0.3	5.2 ± 0.3	0.26 ± 0.01	44.1 ± 2.6	0.51 ± 0.03	4.0 ± 1.9	1.7 ± 0.36	7.0 ± 1.3	4.6 ± 1.1
**T2-MCP-10**	5.5 ± 0.7	6.0 ± 1.1	3.0 ± 0.2	1.7 ± 0.2	6.8 ± 0.6	5.7 ± 0.9	0.93 ± 0.08	5.1 ± 1.0	2.3 ± 0.4	56 ± 15
**T2-MCP-11**	17.0 ± 0.1	29.3 ± 7.3	1.4 ± 0.1	2.7 ± 0.2	4.3 ± 0.4	19 ± 1				
**T2-MCP-12**	524 ± 133	611 ± 87				910 ± 300				
**T2-MCP-13**	8.4 ± 1.9	6.8 ± 2.1	11 ± 4	2.4 ± 0.2	4.8 ± 0.3	2.3 ± 0.6	2.2 ± 0.7	8.3 ± 0.6	1.4 ± 0.1	40 ± 10

a IC_50_ values against human TMPRSS2 (huTMPRSS2) and mouse TMPRSS2 (muTMPRSS2) are the average ± SD (*n* = three independent experiments). Camostat mesylate (Camo) was included as a control inhibitor. Associated binding kinetic constants *k*_on_ and *k*_off_, the binding half-life (*t*_1/2_ = ln(2)/*k*_off_), and calculated equilibrium dissociation constant *K*_D_ of MCPs binding to immobilized huTMPRSS2 and the catalytically inactive TMPRSS2^S441A^, where the catalytic Ser was substituted with an Ala residue, were determined by SPR. Values shown are the average ± SD (*n* = three independent experiments). For each experiment, *k*_on_, *k*_off_, and *K*_D_ values were obtained independently from sensorgram fitting. Consequently, the reported mean *K*_D_ values may not exactly equal the ratio of the reported mean *k*_off_ and mean *k*_on_ values. MCPs where no binding kinetic constants or equilibrium dissociation constants could be calculated when tested up to 300 nM are left blank. N.T. = not tested.

To identify the mechanism of action of the MCPs, we performed surface plasmon resonance (SPR) binding studies (Figures 2c–h, S3, and Table 1). First, we confirmed that the MCPs displayed strong affinities toward TMPRSS2, with *K*_D_ values in good agreement with the inhibitory activities (Table 1). Binding sensorgrams of the two potent inhibitors, T2-MCP-06 and T2-MCP-09, showed very slow binding dissociation kinetic constants (*k*_off_), characteristic of a strong TMPRSS2-binding interaction (Figure 2c,d). To identify whether the MCPs directly bind to the active site of TMPRSS2, we blocked the active site with the activity-based probe (ABP) PK401-cmk (Ac-hCha-Phe­(guan)-Oic-Arg-cmk). This ABP is identical to the previously reported PK401 except for the substitution of the diphenyl phosphonate warhead with a chloromethyl ketone (cmk) warhead that enables irreversible covalent binding (Figure S4a).^59^ The probe was designed to occupy the S1–S4 subsites on the protease (nomenclature according to Schechter and Berger^60^) and selectively inhibits neutrophil serine protease 4. However, we discovered that PK401-cmk showed strong inhibition of TMPRSS2 (Figure S4b). SPR binding experiments with TMPRSS2^ABP^, in which the TMPRSS2 active site was covalently blocked by ABP PK401-cmk, demonstrated that all MCPs completely lost binding affinity (Figures 2e,f and S3), suggesting the MCPs directly bind to the active-site region of TMPRSS2. Further evidence to support active-site binding of the MCPs was derived from SPR binding experiments with the catalytically inactive TMPRSS2, where the catalytic Ser was substituted with an Ala residue (S441A) (TMPRSS2^S441A^). All MCPs, including the potent T2-MCP-06 and T2-MCP-09, showed reduced binding affinities for TMPRSS2^S441A^, albeit to variable degrees (Table 1, Figures 2g,h and S3). For instance, while the binding loss of T2-MCP-02 was negligible, the binding of the MCPs T2-MCP-04, T2-MCP-06, T2-MCP-07, and T2-MCP-11 was no longer detectable (Table 1, Figures 2g and S3), suggesting that these four MCPs engage in key binding interactions with the catalytic serine of TMPRSS2. In conclusion, these findings strongly suggest that the MCPs, including the lead hit T2-MCP-09, bind to the active-site region of TMPRSS2 to inhibit enzyme activity of both human and murine TMPRSS2.

### Macrocyclic Peptides Prevent HKU1 Coronavirus Spike Protein Binding to TMPRSS2

Recent studies have demonstrated that TMPRSS2 engages the HKU1 coronavirus spike protein receptor-binding domain (HKU1-S-RBD) through its active-site region without proteolytic cleavage, serving as a receptor for coronavirus entry.^22–25^ Because the MCPs likewise target the TMPRSS2 active-site region, we examined their ability to block HKU1-S-RBD binding. First, we confirmed that HKU1-S-RBD (Uniprot ID Q14EB0 isoform 1B, amino acids N323–L607) bound to recombinant TMPRSS2 (*K*_D_ 400 ± 7 nM), in line with previous studies (Figure S5a). Interestingly, we observed that HKU1-S-RBD shows no binding to active-site-blocked TMPRSS2^ABP^ (Figure S5a). To understand the competitive binding nature of the activity-based probe PK401-cmk, we overlaid a structurally similar probe (Ac-KQLR-cmk) bound to the TTSP Hepsin (PDB 1Z8G) with a structure of the HKU1 spike protein bound to TMPRSS2 (PDB 8Y8A). As proposed by McCallum et al., using similar modeling,^23^ Figure S5b shows that the P4 residue of the probe was predicted to clash with one of the major binding loops of the HKU1 spike protein. Because the PK401-cmk probe used in this study also contains a bulky P4 residue (Ac-hCha), it is likely that the P4 residue occupies this critical region of the HKU1-S binding epitope on TMPRSS2, thereby preventing spike protein binding.

Next, we performed SPR competition binding experiments, where we monitored HKU1-S-RBD binding to immobilized TMPRSS2 in the presence of the panel of six MCPs and measured their inhibitory activity by determining the concentration-dependent reduction of steady-state binding. The small covalent inhibitor camostat mesylate did not block HKU1-S-RBD binding (Figure 3), consistent with its binding limited to the S1 pocket of the TMPRSS2 active site,^35^ thereby supporting that TMPRSS2 receptor function is independent of its enzymatic activity. Of the six MCPs tested, only T2-MCP-01, T2-MCP-06, T2-MCP-07, and T2-MCP-13 exhibited strong and concentration-dependent inhibition of HKU1-S-RBD binding to TMPRSS2, whereas T2-MCP-09 and T2-MCP-10 displayed poor inhibition (Figure 3). Because attempts to obtain crystal structures of TMPRSS2:MCP complexes were unsuccessful, we used available structures of other cyclic peptides bound to TMPRSS2-related trypsin-fold serine proteases to gain molecular insights into how some of the tested MCPs could function as dual inhibitors of enzyme and receptor function.

**Figure 3:**
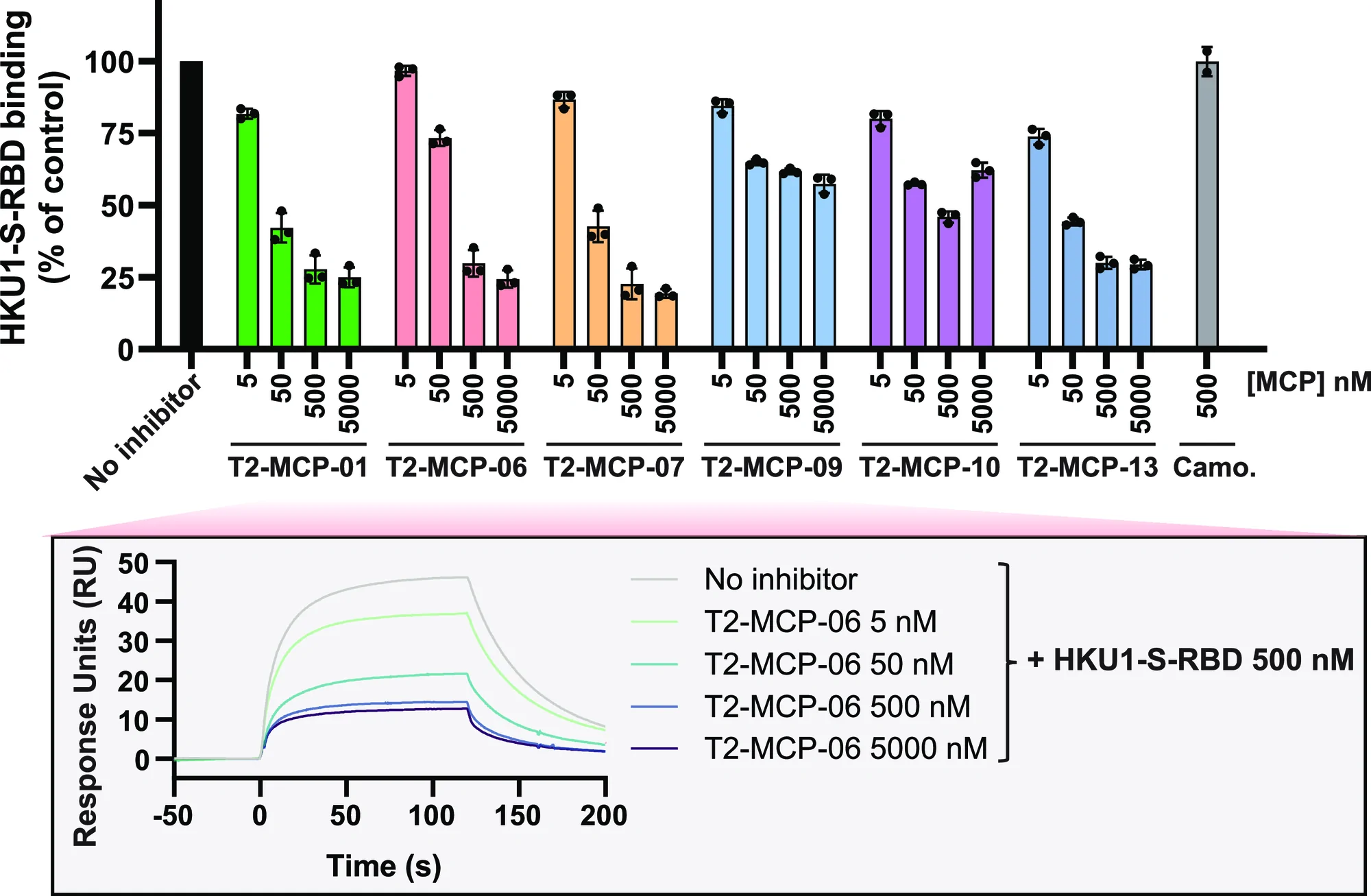
MCPs inhibit binding of TMPRSS2 to HKU1 spike protein. (Top) SPR A-B-A competition binding experiments showing HKU1-S-RBD (HKU1 spike protein receptor-binding domain) (500 nM) binding to immobilized huTMPRSS2 in the presence of selected MCPs (5–5000 nM) or the control inhibitor camostat mesylate (Camo.). Data shows the average ± SD of three independent experiments. (Bottom) SPR sensorgrams showing binding of HKU1-S-RBD (500 nM) to immobilized huTMPRSS2 in the presence of increasing concentrations of T2-MCP-06 (5–5000 nM). Sensorgrams are representative of three independent experiments.

Soybean trypsin inhibitor-1 (SFTI-1) and upain-1 are cyclic peptide inhibitors of trypsin-fold serine proteases with ring sizes comparable to those of the MCPs identified in this study.^61–63^ Importantly, both SFTI-1 and upain-1 bind to the catalytic center of their target proteases, a property shared by the TMPRSS2-binding MCPs as shown by biochemical studies (Table 1, Figures 2 and S3). To explore how only a subset of MCPs effectively inhibit HKU1-S-RBD binding to TMPRSS2, we compared structures of SFTI-1- and upain-1-bound proteases with a TMPRSS2:HKU1-S-RBD complex (Figure S5c). These comparisons suggest that active-site occupancy alone may be insufficient to block HKU1-S-RBD binding. Rather, the orientation of the bound macrocycle appears to be critical. While upain-1 binds close to the HKU1-S-RBD binding site without engaging it, the perpendicularly oriented ring of SFTI-1 extends along the catalytic cleft toward the major HKU1-S-RBD binding loop, resulting in a major steric overlap with the binding interface (magenta residues in Figure S5c). We thus propose that MCPs that demonstrate dual inhibition of both the enzymatic and receptor functions of TMPRSS2 may adopt an SFTI-1-like binding mode that bridges the catalytic center and the adjacent ligand binding site. Conversely, the weak HKU1-S-RBD binding inhibitors T2-MCP-09 and T2-MCP-10 may bind in an alternative orientation that preserves potent enzyme inhibition while failing to effectively engage the HKU1-S-RBD:TMPRSS2-binding interface.

### Macrocyclic Peptides Are Highly Selective for TMPRSS2

For further characterizations and cell-based studies, we focused on the two most potent TMPRSS2 enzyme MCP inhibitors, T2-MCP-06 and T2-MCP-09. Their binding to the TMPRSS2 active site, a region that is structurally conserved among trypsin-fold serine proteases, raised potential concerns about target selectivity. In fact, a common undesirable feature of peptidomimetic active-site inhibitors of TMPRSS2 reported to date is their poor protease selectively for TMPRSS2.^31,32,34^ Therefore, we tested the two MCPs against a panel of 22 trypsin-fold serine proteases, of which 17 have specificity for Arg/Lys at the P1 position. Among these were several other TTSP family members, including TMPRSS11D and TMPRSS13, which are capable of cleaving the SARS-CoV-2 spike protein to enable viral entry.^16,17^ For the recombinant expression of TMPRSS6, TMPRSS11D, and TMPRSS13, we employed the same EK-cleavage design strategy used for TMPRSS2 (see Figure S6 for protein construct designs and enzyme substrate cleavage activity of the engineered TTSPs).^58^ Both T2-MCP-06 and T2-MCP-09 exhibited remarkable selectivity, with little to no inhibitory activity against all 22 proteases tested at a concentration of 1 μM, which is >200-fold and >1000-fold above their respective IC_50_ values against TMPRSS2 (Figure 4). At the concentrations tested, the MCPs showed no appreciable inhibition (<20%) of any protease examined, except for TMPRSS13 and β-tryptase, which were inhibited by T2-MCP-09 by approximately 40% and 50%, respectively. By contrast, camostat mesylate, when tested at 1 μM, showed >80% inhibitory activity against 10 of the proteases, including strong inhibition against all of the TTSPs tested (Figure 4).

**Figure 4:**
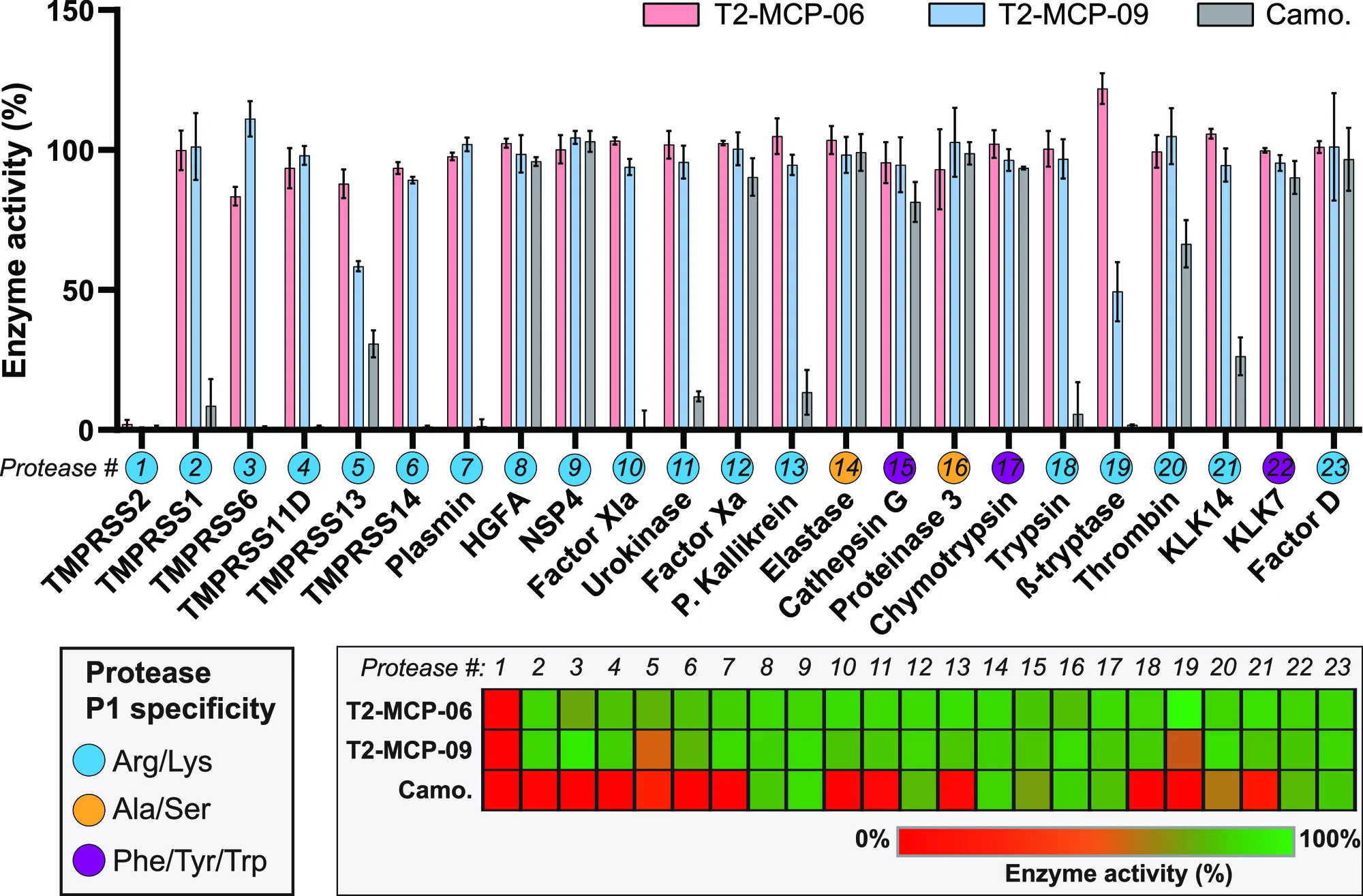
MCPs have strong selectivity for TMPRSS2 among trypsin-fold serine proteases. (Top) The inhibitory activities of the two lead MCPs, T2-MCP-06 and T2-MCP-09, were tested against a panel of trypsin-fold serine proteases, including several TMPRSS2-related TTSP family members. Camostat mesylate (Camo.) was used as a reference. Compounds were investigated at a concentration of 1 μM. Relative enzymatic activity (%) refers to the normalized fluorescence units in the absence of inhibitors. The preferred P1 substrate specificity of each protease is highlighted as colored circles, and each protease is numbered (increasing numbers from left to right). (Bottom) The inhibitory activities are shown as a heat-map plot, where the protease numbering is the same as in the above graph. Data shown are the average ± SD of three independent experiments.

### Macrocyclic Peptides Inhibit Cell Surface-Expressed TMPRSS2

Thus far, all experiments with MCPs have been carried out with engineered recombinant TMPRSS2 encompassing the soluble extracellular domain. To assess inhibition of full-length TMPRSS2 in a cellular context, Vero E6 cells transiently expressing human TMPRSS2 were used. Both T2-MCP-06 and T2-MCP-09 inhibited cell surface TMPRSS2 activity toward the fluorogenic substrate Boc-QAR-AMC in a concentration-dependent manner with IC_50_ values of 99 ± 20 nM and 9.8 ± 2.6 nM, respectively (Figure 5a and Table S2). The superior potency of T2-MCP-09 against pericellular TMPRSS2 is in excellent agreement with the results from enzymatic assays with recombinant TMPRSS2 (Table 1).

**Figure 5:**
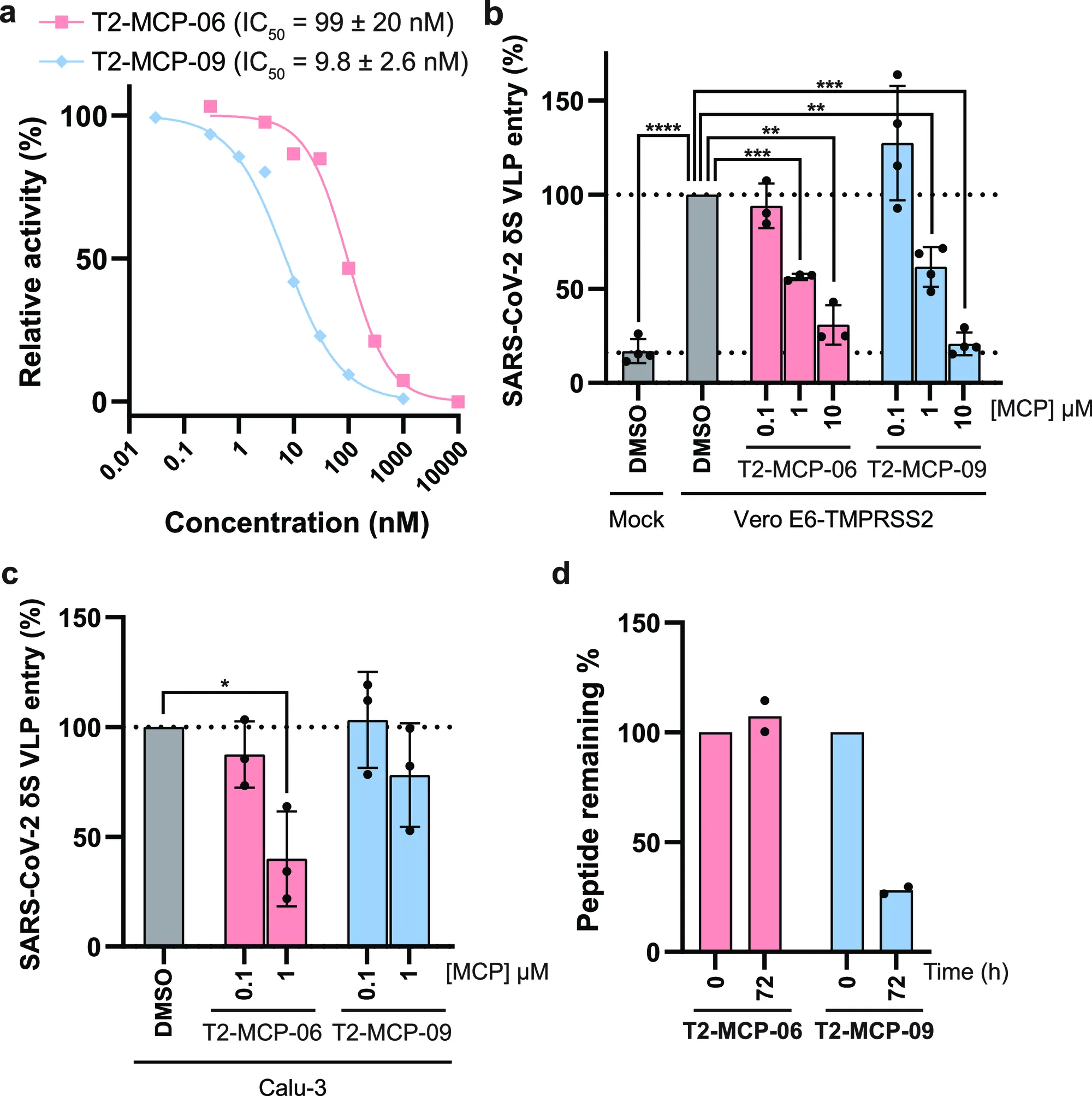
MCPs inhibit pericellular TMPRSS2 activity and reduce cellular entry of SARS-CoV-2 δS virus-like particles (VLPs). (a) T2-MCP-06 and T2-MCP-09 inhibit pericellular cleavage of the fluorogenic substrate Boc-QAR-AMC by Vero E6 cells expressing a TMPRSS2-Myc-His construct (Vero E6-TMPRSS2). IC_50_ values were calculated by generating a nonlinear regression with variable slope (four parameters) analysis from a log­[compound] versus fluorescence data plot. Representative IC_50_ curves are shown, with the mean value ± SD of three (T2-MCP-06) or four (T2-MCP-09) independent experiments. Data is graphically represented by normalizing fluorescence data using the top (100%) and bottom (0%) parameters of the fitted curve used for IC_50_ calculation. These normalized data were then fitted using a four-parameter variable-slope nonlinear regression. (b) Infection of Vero E6-TMPRSS2 cells with SARS-CoV-2 δS VLPs in the presence of T2-MCP-06 or T2-MCP-09 at concentrations of 0.1, 1, 10 μM or DMSO (0.1%). Cells were pretreated with compounds at indicated concentrations or with DMSO for 1 h before infection with VLPs and incubated for 24 h. Inhibitory activities were normalized to Vero E6-TMPRSS2 cells infected with δS VLPs in the presence of DMSO. Mock-transfected Vero E6 infected cells (Mock) were included as a control. Data shown are the average ± SD of three independent experiments. (c) Infection of Calu-3 cells with SARS-CoV-2 δS VLPs in the presence of T2-MCP-06 or T2-MCP-09 at concentrations of 0.1, 1 μM, or DMSO (0.1%). Cells were pretreated with compounds at the indicated concentration or with DMSO for 1 h before infection with VLPs and incubated for 72 h. Vehicle condition (DMSO) infected with δS VLPs is set at 100%, and data is shown in percentage relative to vehicle condition with background signal subtracted (infection with VLPs lacking spike glycoprotein). Data shown are the average ± SD of three independent experiments. Statistical analyses (b, c) were carried out by a Welch’s *t* test: *P < 0.05, **P < 0.01, ***P < 0.001, and ****P < 0.0001. (d) Stability of T2-MCP-06 and T2-MCP-09 upon exposure to Calu-3 cells for 72 h. Data shows the average of two independent experiments.

### Macrocyclic Peptides Inhibit Cellular Uptake of Virus-like Particles Pseudotyped with SARS-CoV-2 Spike Protein

To study the impact of pericellular inhibition of TMPRSS2 by MCPs in the context of SARS-CoV-2 infection, virus-like particles (VLPs) pseudotyped with SARS-CoV-2 spike protein were used. Because VLP uptake in the tested cell lines is enhanced by TMPRSS2-mediated cleavage of the SARS-CoV-2 spike protein at the S2′ site,^4,31,36,43^ we first validated that selected MCPs effectively inhibit this cleavage using recombinant proteins. We confirmed that recombinant TMPRSS2 cleaves a SARS-CoV-2 spike protein in a concentration- and time-dependent manner (Figure S7a). Under experimental conditions where recombinant TMPRSS2 (50 nM) fully cleaved the spike protein at the S2′ site, both T2-MCP-06 and T2-MCP-09 prevented spike cleavage in a dose-dependent manner (Figure S7b). The superior potency of T2-MCP-09 in this assay agrees with its stronger binding affinity for TMPRSS2 (Table 1).

Having confirmed MCP inhibition of spike protein cleavage, we next evaluated their ability to prevent VLP uptake using the engineered Vero E6-TMPRSS2 cell line and the lung epithelial cell line Calu-3, which endogenously expresses low levels of TMPRSS2.^64^ For these experiments, we selected the delta variant of the SARS-CoV-2 spike protein (δS), which is known to be efficiently cleaved by TMPRSS2-expressing cells.^20^ First, we confirmed that Vero E6-TMPRSS2 cells were highly susceptible to SARS-CoV-2 δS VLP entry compared to mock-transfected Vero E6 cells (Figure 5b). Both T2-MCP-06 and T2-MCP-09 reduced SARS-CoV-2 δS VLP entry into Vero E6-TMPRSS2 cells in a concentration-dependent fashion (Figure 5b). However, the ∼10-fold greater potency of T2-MCP-09 compared with T2-MCP-06 in inhibiting cell surface TMPRSS2 activity (Figure 5a) did not translate into enhanced antiviral efficacy, as both compounds were similarly effective at preventing viral entry. Moreover, T2-MCP-09 showed no activity at inhibiting SARS-CoV-2 δS VLP entry in Calu-3 cells up to 1 μM, while T2-MCP-06 reduced VLP entry by ∼60% when tested at 1 μM (Figure 5c).

### Improving the Proteolytic Stability of T2-MCP-09

We hypothesized that the compromised antiviral activity of T2-MCP-09 in the cell assays reflected stability liabilities exacerbated by the longer Calu-3 incubation period (3 days) relative to Vero E6 experiments (1 day). We confirmed that T2-MCP-09 was degraded when incubated with Calu-3 cells for 72 h (28% remaining), whereas T2-MCP-06 was highly stable (see Figure 5d). To assess whether TMPRSS2 contributes to T2-MCP-09 degradation, we incubated both MCPs with a high concentration of recombinant TMPRSS2 (6 μM). Under these conditions, T2-MCP-09 was extensively cleaved, with only 28% remaining after 24 h, whereas T2-MCP-06 exhibited substantially greater stability (85% remaining) (Figure S8a). These results mirror those obtained in Calu-3 cell incubation experiments and suggest that T2-MCP-09, but not T2-MCP-06, engages TMPRSS2 in a substrate-like manner that renders it susceptible to proteolytic cleavage. To further investigate T2-MCP-09 degradation, we determined the MCP stability in both human and mouse plasma. We found that T2-MCP-09 was rapidly degraded, whereas T2-MCP-06 remained stable (Figures 6a and S8b, respectively). The major metabolite of T2-MCP-09, identified by high-resolution mass spectrometry, was generated by a double cleavage at the amide bonds adjacent to Arg10 (Figures 6b and S8c–d). Therefore, it is likely that proteases with P1 specificity for Arg recognize Arg10 as a cleavage-susceptible residue in T2-MCP-09; accordingly, modification of this position may improve peptide stability.

**Figure 6:**
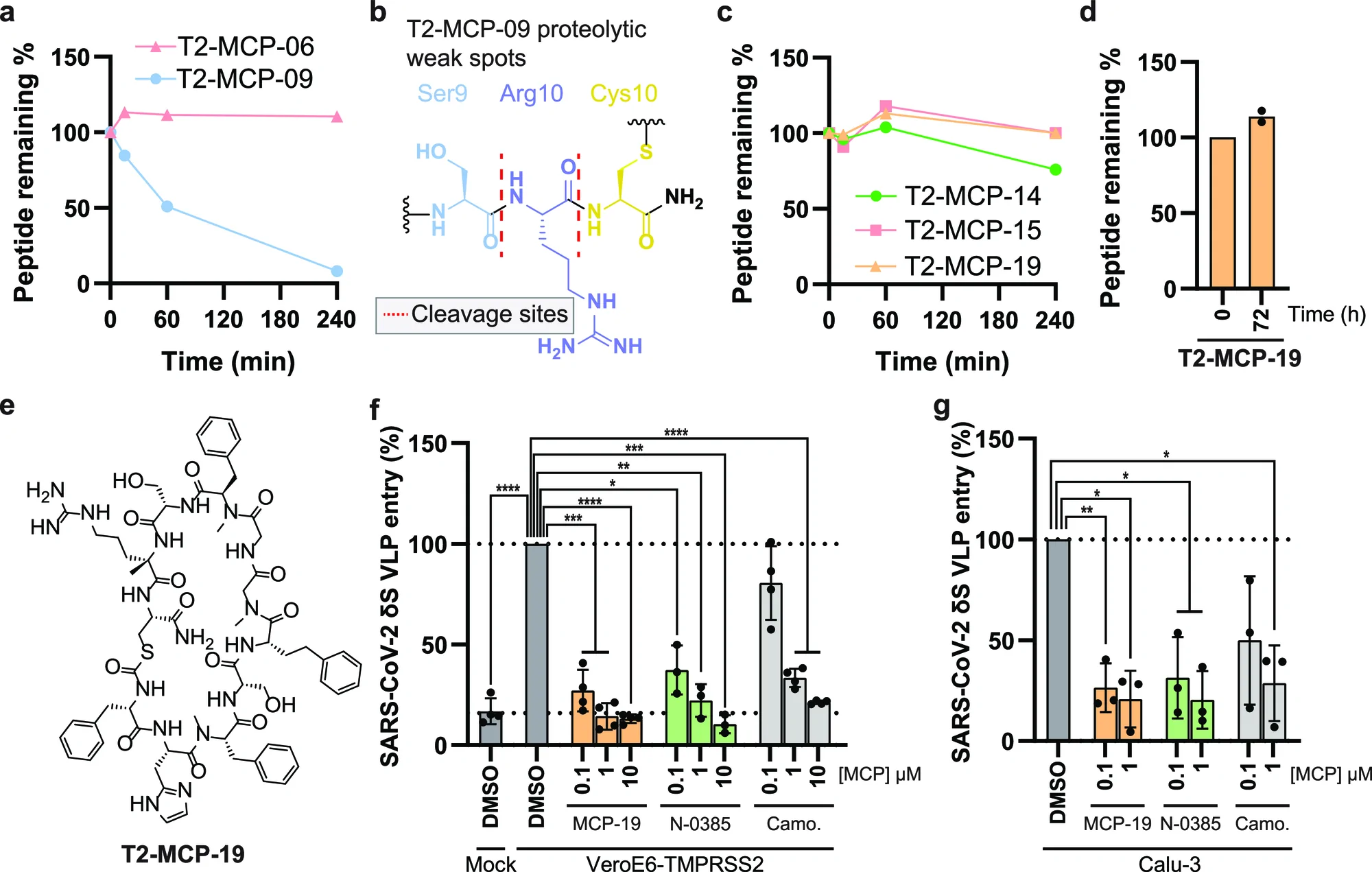
Improving the plasma stability of T2-MCP-09 yields potent TMPRSS2 inhibitors with strong antiviral activities. (a) *In vitro* human plasma stability of T2-MCP-06 and T2-MCP-09. The remaining intact peptides were quantified by LC-MS. Data points are shown as the average of two independent experiments. (b) In human plasma, T2-MCP-09 undergoes proteolytic cleavage at the amide bonds adjacent to Arg10. (c) *In vitro* human plasma stability of designed MCPs T2-MCP-14, T2-MCP-15, and T2-MCP-19. The remaining intact peptides were quantified by LC-MS. Data shows the average of two independent experiments. (d) Stability of T2-MCP-19 upon exposure to Calu-3 cells for 72 h. The remaining intact peptide was quantified by LC-MS. Data shows the average of two independent experiments. (e) 2D chemical structure of T2-MCP-19, the stability-enhanced analogue of T2-MCP-09 containing an α-methylarginine substitution at the TMPRSS2 cleavage site. (f) Infection of Vero E6-TMPRSS2 cells with SARS-CoV-2 δS VLPs was performed in the presence of T2-MCP-19, N-0385, or camostat mesylate (Camo.) at concentrations of 0.1, 1, 10 μM or DMSO (0.1%). Cells were pretreated with compounds at the indicated concentration or with DMSO for 1 h before infection and incubated with VLPs for 24 h. Inhibitory activities were normalized to Vero E6-TMPRSS2 cells infected with δS VLPs in the presence of DMSO. Mock-transfected Vero E6 cells and published benchmark TMPRSS2 inhibitors camostat mesylate and N-0385 were included as controls. Data shown are the average ± SD of three independent experiments. Statistical analyses were carried out by a Welch’s *t* test: *P < 0.05, **P < 0.01, ***P < 0.001, and ****P < 0.0001. (g) Infection of Calu-3 cells with SARS-CoV-2 δS VLPs in the presence of T2-MCP-19 and the published benchmark TMPRSS2 inhibitors N-0385 and camostat mesylate (Camo.) at concentrations of 0.1, 1 μM or DMSO (0.1%). Cells were pretreated with compounds at the indicated concentration or with DMSO for 1 h before infection and incubated with VLPs for 72 h. Vehicle condition (DMSO) infected with δS VLPs is set at 100%, and data is shown in percentage relative to vehicle condition with background signal subtracted (infection with VLPs deprived of spike glycoprotein). Data shown are the average ± SD of three independent experiments. Statistical analyses were carried out by a Welch’s *t* test: *P < 0.05, **P < 0.01.

We confirmed that Arg10 was important for inhibitory activity (Figure S9a). Therefore, we sought to improve the proteolytic stability of T2-MCP-09 without compromising TMPRSS2 inhibitory activity by replacing Arg10 and the adjacent Cys11 with noncanonical amino acids. We designed MCPs that incorporated d-Arg (r), *N* ^α^-methyl Arg (MeR), *N* ^ω^-methyl Arg (RMe), citrulline (Cit), homo-Arg (hR), α-methyl Arg (αMeR), and ornithine (Orn) at position 10, and *N*-methyl Cys (MeC) at position 11 (see Figure S9b for amino acid chemical structures and Figure S9c for MCP designs). Interestingly, all MCPs bearing Arg side-chain modifications—T2-MCP-16 ([R10RMe]), T2-MCP-17 ([R10Cit]), T2-MCP-18 ([R10hR]), and T2-MCP-20 ([R10Orn])—showed markedly reduced TMPRSS2 inhibitory activity compared with the parent peptide T2-MCP-09 (Table 2 and Figure S9d). In addition, T2-MCP-21 ([C11MeC]) had reduced inhibitory activity. T2-MCP-14 ([R10r]) showed low nanomolar inhibitory activity, suggesting stereochemical inversion of the Arg side-chain was well tolerated. Additionally, the MCPs T2-MCP-15 ([R10MeR]) and T2-MCP-19 ([R10αMeR]), which contained modifications to the amino acid backbone, strongly inhibited TMPRSS2, with T2-MCP-19 (IC_50_ = 0.45 ± 0.08 nM) displaying even more potent activity than the parent T2-MCP-09. Both T2-MCP-15 and T2-MCP-19 also showed improved binding affinities for TMPRSS2, which were mainly driven by slower dissociation rates (Table 2 and Figure S10), with T2-MCP-19 displaying a *K*_D_ value of 80 pM. Accordingly, T2-MCP-19 also showed a 6-fold improvement in its inhibitory activity against pericellular TMPRSS2, with an IC_50_ of 1.6 ± 0.6 nM (Figure S9e), when compared to its parent compound T2-MCP-09 (Figure 5a, Table S2). The inhibitory potency of T2-MCP-19 for pericellular TMPRSS2 is similar to that of the published TMPRSS2 inhibitor N-0385 (reported IC_50_ = 1.9 nM).^31^

**Table 2:** *In Vitro* Inhibitory Activities and Binding Affinities of Rationally Designed MCP Analogues of T2-MCP-09 For Recombinant TMPRSS2^a^

	MCP mutation		IC_50_ (nM)		TMPRSS2			
MCP	p10	p11	huTMPRSS2	muTMPRSS2	*k*_on_ (1 × 10^5^ M^–1^s^–1^)	*k*_off_ (1 × 10^–3^ s^–1^)	*t*_1/2_ (min)	*K*_D_ (nM)
**T2-MCP-09**	**R**	C	0.85 ± 0.08	1.3 ± 0.3	5.2 ± 0.3	0.26 ± 0.01	44.1 ± 2.5	0.51 ± 0.03
**T2-MCP-14**	**r**	C	2.1 ± 0.4	2.5 ± 0.9	11.6 ± 1.5	1.7 ± 0.06	6.7 ± 0.3	1.5 ± 0.2
**T2-MCP-15**	**MeR**	C	1.3 ± 0.2	1.0 ± 0.2	4.4 ± 1.0	0.12 ± 0.02	102.4 ± 20.9	0.27 ± 0.09
**T2-MCP-16**	**RMe**	C	>1,000	NT	NT	NT		NT
**T2-MCP-17**	**Cit**	C	>3,000	NT	NT	NT		NT
**T2-MCP-18**	**hR**	C	524 ± 209	NT	NT	NT		NT
**T2-MCP-19**	**αMeR**	C	0.45 ± 0.08	0.67 ± 0.04	8.4 ± 0.6	0.06 ± 0.02	189.6 ± 49.9	0.08 ± 0.02
**T2-MCP-20**	**Orn**	C	246 ± 118	NT	NT	NT		NT
**T2-MCP-21**	R	**MeC**	50 ± 1	NT	NT	NT		NT

a The parent compound T2-MCP-09 is shown for reference. Amino acid mutations at either the position 10 (p10, parent Arg) or the position 11 (p11, parent Cys) are shown and highlighted in bold in the table (see Figure S9b for chemical structures of the noncanonical amino acids). IC_50_ values against huTMPRSS2 and muTMPRSS2 are the average ± SD (*n* = three independent experiments). Associated binding kinetic constants *k*_on_ and *k*_off_, and calculated equilibrium dissociation constant *K*_D_ of MCPs binding to immobilized TMPRSS2 are the average ± SD (*n* = three independent experiments). For each experiment, *k*_on_, *k*_off_, and *K*_D_ values were obtained independently from sensorgram fitting. Consequently, the reported mean *K*_D_ values may not exactly equal the ratio of the reported mean *k*_off_ and mean *k*_on_ values. N.T. = not tested.

The protease selectivity profile of the three potent R10 derivatives (T2-MCP-14, T2-MCP-15, and T2-MCP-19) was comparable to that of T2-MCP-09, showing mostly negligible inhibition for 20 of the selected 22 proteases when tested at 1 μM (Figure S11a). T2-MCP-19 showed moderate inhibition of TMPRSS13 and β-tryptase (IC_50_ = 0.73 ± 0.22 μM and 0.20 ± 0.08 μM, respectively; Figure S11b,c). However, this compound still displayed selectivity indices >1000-fold and >400-fold in favor of TMPRSS2 over these two proteases.

Replacing the proteolytically labile Arg10 residue with the noncanonical amino acids drastically improved proteolytic stability, as T2-MCP-14, T2-MCP-15, and T2-MCP-19 all showed high human and mouse plasma stability compared to T2-MCP-09 (Figures 6c and S12a, respectively). In line with these results, T2-MCP-19, unlike the parent T2-MCP-09, was no longer susceptible to cleavage when incubated with Calu-3 cells (Figure 6d). Interestingly, while T2-MCP-19 likely binds TMPRSS2 in a substrate-like manner, as observed for T2-MCP-09, it is resistant to TMPRSS2-mediated cleavage (Figure S12b), highlighting the stabilizing effect of the α-methyl Arg modification.

### Stability-Enhanced T2-MCP-19 Has Improved Inhibition of SARS-CoV-2 δS VLP Entry

Given the robust stability, potent TMPRSS2 inhibitory activity, and excellent proteolytic selectivity of T2-MCP-19 (see Figure 6e for the 2D chemical structure), we proceeded to evaluate its activity. T2-MCP-19 strongly reduced TMPRSS2-dependent SARS-CoV-2 δS VLP entry into Vero E6-TMPRSS2 cells (Figure 6f), limiting VLP entry to 27% at 0.1 μM, and to background levels (mock-transfected Vero E6 cells) at 1 μM and 10 μM. Similarly, T2-MCP-19 significantly reduced SARS-CoV-2 δS VLP entry into lung epithelial Calu-3 cells (Figure 6g), limiting VLP entry to 26% and 21% at concentrations of 0.1 μM and 1 μM, respectively. Notably, T2-MCP-19 not only showed substantially improved potency over T2-MCP-09 but also performed on par with the benchmark TMPRSS2 inhibitors camostat mesylate and the peptidomimetic N-0385 in both models.^4,31^

## Discussion

This study establishes macrocyclic peptides (MCPs) as a potent and selective class of inhibitors of the type II transmembrane serine protease TMPRSS2, a host factor required for the entry of multiple respiratory viruses. Using an mRNA display platform with genetic code reprogramming, we identified macrocyclic peptides that bind the catalytic region of TMPRSS2 with nanomolar-to-picomolar affinities while exhibiting exceptional selectivity across a large panel of related serine proteases. Optimization of the lead peptide T2-MCP-09 through backbone modification yielded the stability-enhanced analogue T2-MCP-19, which retained picomolar binding affinity for TMPRSS2 while displaying markedly improved proteolytic stability and potent inhibition of SARS-CoV-2 spike-mediated viral entry in lung epithelial cells. Together, these results demonstrate that macrocyclic peptides can achieve highly selective targeting of TMPRSS2 and underscore the promise of this new modality for the development of host-directed antivirals against respiratory viruses.

Achieving selectivity has been a persistent challenge in the development of TMPRSS2 inhibitors because of the highly conserved active-site architecture of trypsin-fold serine proteases. Most reported inhibitors rely on short peptidomimetic scaffolds that mimic natural substrates and incorporate reactive warheads to achieve sufficient potency. While such approaches have yielded potent inhibitors, including the reversible covalent compound N-0385, they often display limited selectivity across related proteases due to their substrate-like binding modes.^31,32,37^ Remarkably, the MCPs identified here bind to the catalytic region of TMPRSS2 while maintaining exceptional selectivity against 22 structurally related proteases. We hypothesize that the large and conformationally constrained macrocyclic scaffold enables interactions that extend beyond the canonical substrate binding pockets, allowing the peptides to engage structural determinants unique to TMPRSS2 and to achieve a degree of selectivity difficult to attain by traditional substrate-like inhibitors. Similar selectivity advantages have been reported for other macrocyclic peptides discovered through mRNA display, highlighting the ability of this modality to target enzyme active sites with exceptional precision.^45,46^ While the protease selectivity panel provides an important measure of biochemical specificity, it should not be interpreted as a direct predictor of *in vivo* pharmacological behavior. The ultimate therapeutic utility of TMPRSS2 inhibitors will depend on additional factors, including pharmacokinetic properties, tissue distribution, and exposure at sites of viral infection. Nevertheless, the observed selectivity profile suggests that TMPRSS2 can be targeted with minimal inhibition of closely related serine proteases, thereby reducing a key liability that has limited the previously reported inhibitors. The enhanced plasma stability of T2-MCP-19 further supports the feasibility of advancing this scaffold to future *in vivo* evaluation.

Mechanistic analyses of the initial lead peptide, T2-MCP-09, suggested that segments of its macrocyclic ring engage the TMPRSS2 active site in a substrate-like manner. Reduced binding affinity toward catalytically inactive TMPRSS2, combined with susceptibility to proteolytic cleavage at the Arg10 residue, indicates that the peptide mimics key interactions involved in natural substrate recognition. While such interactions likely contribute to the high affinity of the peptide, they also introduce stability liabilities that limit cellular antiviral activity. Substitution of the Arg10 residue with an α-methylarginine preserved the essential electrostatic interactions required for binding while preventing proteolytic hydrolysis, resulting in the optimized inhibitor T2-MCP-19. Notably, α-methylation enhanced both potency and proteolytic stability, and we propose this as a valuable strategy for the broader design of inhibitors of trypsin-fold proteases. The resulting compound exhibited picomolar affinity, excellent plasma stability, and strong inhibition of SARS-CoV-2 virus-like particle entry in both engineered and physiologically relevant lung epithelial cell models. These findings highlight the importance of stability engineering when translating potent biochemical inhibitors into functional antiviral agents.

Recent structural studies have demonstrated that TMPRSS2 can function not only as a protease but also as a cell surface receptor that facilitates the entry of the human coronavirus HKU1 independently of its catalytic activity.^22–25^ The observation that MCPs can interfere with both enzymatic- and receptor-mediated functions suggests that active-site-directed ligands may modulate multiple aspects of TMPRSS2 biology. Interestingly, for some MCPs, we observed a functional divergence where the degree of catalytic inhibition was decoupled from blockade of receptor binding. This suggests that individual MCPs engage specific subsites within the catalytic region that may not effectively overlap with the HKU1 spike protein binding interface. Such functional diversity could potentially be exploited to develop both dual-function inhibitors and inhibitors that preferentially target TMPRSS2 enzyme activity.

Beyond their therapeutic potential, the MCPs described herein represent valuable chemical probes for studying TMPRSS2 biology. A major limitation of currently available TMPRSS2 inhibitors is their broad activity against related serine proteases, which can complicate the assignment of biological effects specifically to TMPRSS2. The exceptional selectivity of the MCPs identified in this study enables a more precise interrogation of TMPRSS2-dependent pathways. In viral systems, these compounds may facilitate studies investigating the contribution of TMPRSS2 to respiratory virus entry, viral adaptation, and compensatory utilization of alternative host proteases. Furthermore, the observation that selected MCPs inhibit both TMPRSS2 catalytic activity and HKU1 spike protein binding suggests that they may serve as useful tools for dissecting the recently described receptor functions of TMPRSS2 that are independent of proteolysis. The availability of selective MCP inhibitors provides a complementary approach to gene-silencing techniques, which affect both catalytic and noncatalytic functions, including protein–protein interactions. These inhibitors may enable more refined investigations into endogenous TMPRSS2 interacting partners and potential noncatalytic effects on signaling pathways, helping to define physiological and pathological roles of TMPRSS2 beyond viral infection.

Targeting TMPRSS2 also fits within the broader paradigm of host-directed antiviral therapy.^65^ Unlike direct-acting antivirals that target rapidly mutating viral proteins, host-targeted approaches exploit conserved cellular processes required for viral replication and, therefore, may provide broader activity against diverse viral strains while reducing the likelihood of resistance. The COVID-19 pandemic highlighted the importance of identifying host factors that can be targeted to suppress viral entry and replication across multiple variants. TMPRSS2 is particularly attractive in this regard because it plays a critical role in the entry of multiple respiratory viruses, including SARS-CoV-2 and influenza viruses.^4–9^ Furthermore, genetic studies in TMPRSS2-deficient mice have demonstrated that loss of the protease confers protection against viral infection without causing major physiological defects, suggesting that pharmacological inhibition of TMPRSS2 may be well tolerated.^20,21,66^ Recent analyses of antiviral preparedness strategies have emphasized the importance of developing inhibitors of host entry factors such as TMPRSS2 as part of a broader pandemic response framework.^67–69^ Within this context, the highly selective macrocyclic inhibitors described here represent a promising addition to the repertoire of host-directed antiviral modalities. Translation of TMPRSS2-targeting MCPs into therapeutic agents will require further optimization of the formulation, delivery, and manufacturing processes. However, macrocyclic peptides of comparable size and complexity are routinely produced using established solid-phase peptide synthesis methodologies at scales compatible with clinical development.^70^ The high potency and selectivity of T2-MCP-19 provide a promising starting point for future efforts aimed at advancing this class of molecules toward therapeutic applications.

## Conclusion

This work demonstrates that mRNA display can generate macrocyclic peptides capable of selectively targeting a challenging membrane-associated serine protease. The optimized inhibitor T2-MCP-19 combines picomolar binding affinity with exquisite protease selectivity and enhanced stability, which translates into effective blockade of TMPRSS2-dependent viral entry in cell-based models. Beyond their potential as antiviral therapeutics, the exceptional selectivity of the MCPs makes them powerful tools for studying the diverse biological functions of TMPRSS2. More broadly, these findings highlight mRNA display of macrocyclic peptides as a versatile platform for selectively targeting host proteases that have proven difficult to inhibit using conventional small-molecule approaches.

## Experimental Section

### Recombinant Protein Expression and Purification

The following TTSPs were designed using their extracellular domains and replacing their autocatalytic activation sequences with an enterokinase (EK) tag: human TMPRSS2 (based on Uniprot ID O15393; residues M109–S492; [S251D]­[R252D]­[Q253D]­[S254D]­[R255K]), TMPRSS2^S441A^ (same as TMPRSS2 with additional [S441A] mutation), murine TMPRSS2 (based on Uniprot ID Q9JIQ8; residues W105–S490; [K249D]­[R250D]­[Q251D]­[S252D]­[R253K]), human TMPRSS6 (based on Uniprot ID Q8IU80; residues V447–T811; [G572D]­[P573D]­[S574D]­[S575D]­[R576K]), human TMPRSS11D (based on Uniprot ID O60235; residues A42–I418; [L182D]­[S183D]­[E184D]­[Q185D]­[R186K]), and human TMPRSS13 (based on Uniprot ID Q9BYE2; residues Q187–S562; [A321D]­[M322D]­[T323D]­[G324D]­[R325K]). All proteins were expressed with N-terminal His_6_ tags.

TMPRSS2, TMPRSS2^S441A^, and TMPRSS13 were expressed in Chinese hamster ovary (CHO) cells. Transient transfections were performed at a 2 L working volume in 3 L shake flasks (Corning). Cells were seeded at approximately 6 × 10^6^ cells/mL by removing the spent medium through centrifugation (1100*g* for 10 min) and resuspension in fresh proprietary transfection medium. For transfections, 1 mg/L coding DNA (expression plasmid coding for the gene of interest) and 2.0 mg/L PEIpro (Polyplus-transfection, ref# 101000029) were incubated in proprietary precomplex medium for 5 min before adding to the resuspended cells. Transfected cells were incubated at 37 °C, 5% CO_2_ in a 50 mm orbital diameter shaking incubator (Eppendorf, Model# Innova 2100) on Day 0. The temperature was shifted to 33 °C on Day 1 following a post-transfection feed with proprietary feed medium. After 14 days, the supernatants were centrifuged, collected, and filtered.

Murine TMPRSS2 was expressed in *Trichoplusia ni* (*T. ni*) cells. *T. ni* cells (10 L) were maintained in ESF921 (Expression Systems) at 27 °C, and cells were infected at 2 × 10^6^ cells/mL with a multiplicity of infection of 2.0. After 48 h, the culture was treated with 500 mL of 1 M Tris, pH 8.0, 50 mL of 1 M CaCl_2_, and 10 mL of 1 M NiCl_2_, followed by centrifugation and filtration.

TMPRSS6 was expressed in Sf9 cells. Sf9 cell cultures (10 L) were maintained in ESF921 (Expression Systems) at 27 °C, and cells were infected at 2 × 10^6^ cells/mL with a multiplicity of infection of 2.35. After 72 h, the culture was treated with 500 mL of 1 M Tris, pH 8.0, 50 mL of 1 M CaCl_2_, and 10 mL of 1 M NiCl_2_, followed by centrifugation and filtration.

TMPRSS11D was expressed in High Five (Hi5) cells. Hi5 cell cultures (2 L) were maintained in ESF921 (Expression Systems) at 27 °C, and cells were infected at 2 × 10^6^ cells/mL with a multiplicity of infection of 4.0. After 48 h, the culture was treated with 500 mL of 1 M Tris, pH 8.0, 50 mL of 1 M CaCl_2_, and 10 mL of 1 M NiCl_2_, followed by centrifugation and filtration.

Harvested supernatants containing crude protein were purified by nickel affinity chromatography using either a HisTrap excel 5 mL column (GE Healthcare) or Ni-NTA agarose (Qiagen). After elution with 300 mM imidazole in 50 mM Tris, pH 7.5, 0.3 M NaCl, proteins were further purified by size exclusion chromatography (SEC) using a Sephacryl S-200 column (GE Healthcare) equilibrated with 20 mM HEPES, pH 7.2, and 100 mM NaCl.

Purified protein zymogens were concentrated to 2 mg/mL and activated overnight with EKMax enterokinase (Invitrogen) (0.05 mg/mL final) in 20 mM HEPES, pH 7.2, 150 mM NaCl. Cleaved proteins were confirmed by SDS-PAGE and purified by SEC as described above. Protein-containing fractions were combined and concentrated, and stored at −80 °C.

Biotinylation of TMPRSS2 was performed using EZ-Link NHS-PEG4-Biotin (Thermo Fisher Scientific Inc.) in 20 mM HEPES, pH 7.2, 150 mM NaCl, and the reaction was carried out for 2 h at room temperature. Biotinylation was confirmed by electrospray ionization mass spectrometry (ESI-MS). The biotinylated TMPRSS2 was buffer-exchanged into 20 mM HEPES, pH 7.2, 150 mM NaCl using an Amicon Ultra Centrifugal 10k Filter (Sigma).

HKU1-S-RBD (Uniprot ID Q14EB0, isoform 1B, residues N323–L607) with an N-terminal His_8_ tag was expressed in 293 cells. 293 cell cultures were seeded at 1 × 10^6^ cells/mL and incubated at 37 °C, 5% CO_2_ for 2 h prior to transfection. After 7 days of expression, the supernatants were centrifuged, collected, and filtered. The harvested supernatant was purified using HisTrap excel 5 mL column (GE Healthcare) and further purified by SEC as described above.

### mRNA Display Library Design of MCPs

Four macrocyclic peptide puromycin-conjugated mRNA libraries composed of 10–14-membered scaffolds were constructed using a genetically reprogrammed *in vitro* translation system as previously described.^45,47,71–73^ The libraries were generated with a two degenerate codon system, NNW (Lib4), and NNU (Lib1–3) to generate libraries containing both natural and non-natural amino acids. See Figure S2 for codon designs and the amino acids incorporated within each library. To achieve peptide cyclization following *in vitro* translation, an initiator amino acid N-chloroacetyl-l-phenylalanine (ClAcF) and l-cysteine were incorporated at the N- and C-terminal ends of the MCP scaffolds, respectively, which enabled spontaneous thioether bond formation.

### MCP Library Selections

mRNA display library selections against biotinylated TMPRSS2 were performed as previously described.^73^ Five rounds of selections were performed, with the first round performed for 1 h at 4 °C and all subsequent rounds performed for 20 min at 4 °C. For each round of selections, libraries were mixed with 250 nM of biotinylated TMPRSS2 in binding buffer (25 mM HEPES, pH 7.4, 150 mM NaCl_2_, and 0.05% Tween-20), and TMPRSS2-bound macrocyclic peptides were captured on neutravidin-coated magnetic beads (Cytiva). Nonbinding MCPs were removed by washing captured beads in binding buffer. The bound peptide-cDNA/RNA conjugates were eluted by heating the beads for 5 min at 95 °C. The amount of recovered cDNA was measured by qPCR using SYBR Green I on a QuantStudio 5 thermal cycler (Thermo Fisher Scientific Inc.) and subsequently amplified by PCR for further rounds of selection. After five rounds of selections, the enriched DNAs were sequenced using MiSeq next-generation sequencing (Illumina). Based on analysis of these sequences, 13 MCPs were selected as hits for solid-phase peptide synthesis and further characterization.

### Enzymatic Substrate Cleavage Assays of Selected Recombinant TTSP Family Proteases

All enzymatic assays were carried out in 25 mM Tris, pH 8, 150 mM NaCl, 5 mM CaCl_2_, 0.1% (v/v) Triton X-100 in 384-well black optical bottom plates (Nalge Nunc Int.) in 25 μL reaction volumes using a SpectraMax M5 microplate reader (Molecular Devices) at 25 °C. Recombinant TTSPs (huTMPRSS2, muTMPRSS2, huTMPRSS6, huTMPRSS11D, huTMPRSS13) at increasing concentrations were added to the fluorogenic substrate Boc-QAR-AMC (catalog # ES014, R&D Systems) (0–250 μM). Fluorescence was monitored over 5 min at 380 nm:460 nm (excitation:emission), and reaction velocities (mRFU/min) were plotted against substrate concentration (Michaelis–Menten plots; Figures S1b,c and S6), and curve fitting was performed using the Michaelis–Menten model using GraphPad Prism (v.10.0.2). Data shown for huTMPRSS2 are the average ± SD of three independent experiments. Data for muTMPRSS2, huTMPRSS6, huTMPRSS11D, and huTMPRSS13 are from a single experiment.

### MCP IC_50_ Measurements against Recombinant TMPRSS2

For IC_50_ measurements, dilution series of MCPs were incubated with huTMPRSS2 and muTMPRSS2 for 15 min prior to addition of substrate. The final reaction concentrations were as follows: 0.2 nM huTMPRSS2 or 0.2 nM muTMPRSS2 and 40 μM Boc-QAR-AMC. MCPs were tested using a 3-fold dilution series starting at 1 μM, and the linear reaction velocity rates were normalized to enzymatic activity with assay buffer only containing DMSO (0.1%). Camostat mesylate (Sigma) was also investigated under the same experimental conditions. IC_50_ values were determined by plotting the normalized reaction velocities against MCP concentration, and curve fitting was performed using the log­[inhibitor] *vs* response—variable slope model using GraphPad Prism (v.10.0.2). The IC_50_ values were the mean ± SD of three independent experiments.

### Enzymatic Assays of a Trypsin-Fold Serine Protease Panel

The MCPs T2-MCP-06, T2-MCP-09, T2-MCP-14, T2-MCP-15, T2-MCP-19, and the control inhibitor camostat mesylate were tested in a panel of 23 trypsin-fold serine proteases. MCPs and control inhibitors were incubated at a concentration of 1 μM. Compounds and control inhibitors were incubated with the proteases in assay buffer (50 mM Tris-HCl, 100 mM NaCl, pH 8, 0.1% (v/v) Triton X-100) for 15 min at room temperature prior to the addition of substrates. The linear reaction velocity rates of substrate cleavage were measured on a SpectraMax M5 microplate reader (Molecular Devices) at 25 °C and expressed as a percentage of enzyme activity relative to control (DMSO only). The reported values are the mean ± SD of three independent experiments.

The final concentrations of the proteases and their corresponding substrates in the reaction mixture were as follows: 0.2 nM TMPRSS2, 40 μM Boc-QAR-AMC; 3 nM TMPRSS1 (hepsin), 0.5 mM S-2366; 0.5 nM TMPRSS6, 50 μM Boc-QAR-AMC; 1 nM TMPRSS11D, 10 μM Boc-QAR-AMC; 4 nM TMPRSS13, 50 μM Boc-QAR-AMC; 2 nM TMPRSS14 (matriptase), 25 μM Boc-QAR-AMC; 3 nM plasmin, 0.5 mM S-2366; 20 nM hepatocyte growth factor activator (HGFA), 0.5 mM ADG217L; 4 nM neutrophil serine protease 4 (NSP4), 0.05 mM PK421; 6 nM Factor XIa, 0.5 mM S-2288; 10 nM urokinase, 0.3 mM S-2444; 0.5 nM Factor Xa, 0.3 mM S-2765; 0.25 nM plasma kallikrein, 0.6 mM S-2302; 10 nM neutrophil elastase, 1 mM M4765; 20 nM cathepsin G, 0.5 mM BML-P303 and 0.17 mM DNTB; 10 nM proteinase 3, 0.5 mM BML-P303 and 0.17 mM DNTB; 5 nM chymotrypsin-C, 0.3 mM S-2586; 2.5 nM trypsin-3, 0.5 mM S-2222; 4 nM β-tryptase, 2 mM S-2288, 50 μg/mL heparin sulfate; 0.5 nM thrombin, 0.5 mM S-2238; 1 nM KLK14 (thermolysin activated), 0.01 mM Boc-VPR-AMC; 50 nM KLK7 (thermolysin activated), 0.01 mM Mca-RPKPVE-Nval-WRK­(Dnp)-NH_2_ ES002; 200 nM factor D, 0.2 mM Z-Lys-SBzl and 0.17 mM DNTB. The final concentration of DMSO was 0.2% (v/v).

TMPRSS1 (hepsin), HGFA, ß-tryptase, factor D, and NSP4 were expressed in-house as previously described.^56,74–76^ Coagulation Factor XIa (catalog # HCXIA-0160), Factor Xa (catalog # HCXA-0060), plasmin (catalog # HCPM0140), and thrombin (catalog # HCT-0020) were purchased from Haematologic Technologies. Trypsin-3 (catalog # 3714-SE-010, R&D Systems), KLK7 (catalog # CF 2624-SE-010, R&D Systems), KLK14 (catalog # 2626-SE-010, R&D Systems), and TMPRSS14 (matriptase) (catalog # 3946-SEB, R&D Systems) were purchased from R&D Systems. Neutrophil elastase (catalog # 16–14–051200), proteinase 3 (catalog # 16–14–161820), and cathepsin G (catalog # 16–14–030107) were purchased from Athens Bioscience, Inc. Chymotrypsin-C (catalog # SRP6509, Sigma), plasma kallikrein (catalog # HPKa2650, Enzyme Research Laboratories), and urokinase (catalog # CC4000, Chemicon) were purchased from listed sources. Chromogenic substrates S-2238 (catalog # S820324), S-2288 (catalog # S820852), S-2302 (catalog # S820340), S-2366 (catalog # S821090), S-2444 (catalog # S820357), S-2586 (catalog # S820894), and S-2765 (catalog # S821413) were purchased from Diapharma, and Mca-RPKPVE-Nval-WRK­(Dnp)-NH2 (catalog # ES002) and Boc-VPR-AMC (catalog # ES011) were purchased from R&D Systems. M4765 (catalog # M4765, Sigma-Aldrich) and BML-P303 (catalog # BML-P303–0005, Enzo Life Sciences) were purchased from listed vendors. PK421 was synthesized as previously described.^59^ ADG217L (catalog # ADG217L) was purchased from Sekisui Diagnostics.

For IC_50_ measurements of T2-MCP-19 against TMPRSS13 and β-tryptase, dilution series of T2-MCP-19 (2–10,000 nM) were incubated with the respective proteases for 15 min prior to addition of the substrate. The final protease and substrate concentrations in the assay conditions are mentioned above. IC_50_ values were determined by plotting the normalized reaction velocities against MCP concentration, and curve fitting was performed using the log­[inhibitor] *vs* response–variable slope model using GraphPad Prism (v.10.0.2). The IC_50_ values were the mean ± SD of three independent experiments.

### Surface Plasmon Resonance

TMPRSS2^ABP^, where the active site was blocked with the probe PK401-cmk, was prepared by adding 1 μM biotinylated TMPRSS2 to 100 μM PK401-cmk in 20 mM HEPES, pH 7.2, 150 mM NaCl for 1 h at room temperature. Biotinylated TMPRSS2, TMPRSS2^S441A^, and TMPRSS2^ABP^ were captured on a neutravidin sensor chip (Cytiva) to a response level of approximately 600 RU. Surface plasmon resonance (SPR) experiments were performed at 25 °C using a Biacore T200 instrument. All peptides were prepared in HBS-EP buffer (0.01 M HEPES, pH 7.4, 0.15 M NaCl, 3 mM EDTA, 0.05% (v/v) Surfactant P20). A reference channel was subtracted from the TMPRSS2-captured channels.

Binding kinetics were determined by injecting six serial 3-fold dilutions of peptides for 120 s at a flow rate of 30 μL/min. Peptide maximum concentrations were as follows: T2-MCP-01 300 nM, T2-MCP-02 900 nM, T2-MCP-03 900 nM, T2-MCP-04 900 nM, T2-MCP-05 900 nM, T2-MCP-06 300 nM, T2-MCP-07 300 nM, T2-MCP-08 900 nM, T2-MCP-09 50 nM, T2-MCP-10 300 nM, T2-MCP-11 900 nM, T2-MCP-12 2700 nM, T2-MCP-13 300 nM, T2-MCP-14 50 nM, T2-MCP-15 50 nM, and T2-MCP-19 50 nM. Slow dissociating peptides were removed from the TMPRSS2-captured channels using six repeated 30 s injections of 20 mM sodium acetate, pH 4.2, 1 M NaCl. All peptide binding affinities were derived from the kinetic model analysis and tested using the multi-cycle injection method, except the binding affinity for T2-MCP-12, which was derived from the steady-state affinity model. Data were analyzed using BIAevaluation software (Cytiva, v.1.1.1) using a 1:1 Langmuir binding model. Associated binding kinetic constants *k*_on_ and *k*_off_, the binding half-life (*t*_1/2_), and calculated equilibrium dissociation constant *K*_D_ values were the mean ± SD of three independent experiments.

HKU1-S-RBD binding to TMPRSS2 was tested using a single-cycle injection method, where six serial 3-fold dilutions starting at 3000 nM were injected for 120 s at 30 μL/min. Binding affinity was derived from the steady-state affinity model using the single-cycle injection method. Data were analyzed using BIAevaluation software (Cytiva, v.1.1.1) using a 1:1 Langmuir binding model. The *K*_D_ values were the mean ± SD of three independent experiments.

### SPR Competition Assays Measuring MCP Inhibition of HKU1-S-RBD Binding to TMPRSS2

Competition binding experiments measuring HKU1-S-RBD binding to TMPRSS2 in the presence of MCPs were carried out using the A-B-A injection method. Selected MCPs (5, 50, 500, 5000 nM) were injected over the TMPRSS2 immobilized surface for 120 s before a solution of MCPs (at the same respective concentration used during the presample stage), and HKU1-S-RBD (500 nM) was immediately injected over the TMPRSS2:MCP surface for 120 s. HKU1-S-RBD binding to TMPRSS2 in the presence of MCPs was expressed as a percentage of binding in the absence of MCP. The reported values are the mean ± SD of three independent experiments.

### SARS-CoV-2 Spike Protein Cleavage Assay

Recombinant SARS-CoV-2 spike trimer protein (catalog # SPN-C52Hs, Acro Biosystems; amino acids V16–P1213, lambda variant – Pango lineage: C.37) that contained mutations which stabilize the trimeric prefusion state (F817P, A892P, A899P, A942P, K986P, V987P),^77^ and which abolish S1/S2 cleavage (R683A, R685A) (800 μg/mL) was incubated with recombinant TMPRSS2 (2, 10, 50 nM) in 25 mM Tris, pH 8, 150 mM NaCl, 5 mM CaCl_2_, 0.1% (v/v) Triton X-100 in 25 μL reaction volumes for 1, 2, 4, and 8 h at 37 °C. Recombinant SARS-CoV-2 spike trimer protein control was incubated in assay buffer only for 8 h at 37 °C. SDS–PAGE samples were prepared by adding 4× SDS–PAGE reducing loading buffer and boiling for 5 min at 95 °C before loading onto Novex 8% Tris-Gly gels (Invitrogen). Gels were visualized by Coomassie blue staining using Simply Blue SafeStain (Invitrogen). For MCP competition experiments, T2-MCP-06 and T2-MCP-09 (50, 200, 800 nM) were incubated with TMPRSS2 (50 nM) for 15 min at room temperature prior to adding SARS-CoV-2 spike protein (800 μg/mL). Reaction mixtures were incubated for 2 h at 37 °C and analyzed by SDS-PAGE as described above.

### TMPRSS2-Mediated MCP Cleavage

T2-MCP-06, T2-MCP-09, and T2-MCP-19 (6 μM) were mixed with recombinant TMPRSS2 (6 μM) in 25 mM Tris, pH 8, 150 mM NaCl, 5 mM CaCl_2_, 0.1% (v/v) Triton X-100. Samples were left at room temperature for 0 or 24 h. Samples were quenched with the addition of 0.1% (v/v) TFA (1:1 ratio to sample volume) and vortexed for 5 min. Samples were analyzed by LC-MS/MS (Agilent 6545XT AdvianceBio LC Q/TOF) on a PLRP-S column (1000 Å, 2.1 mm × 50 mm, 8 μm) using a linear gradient of 15–85% (v/v) acetonitrile containing 0.05% (v/v) FA over 4 min. The percentage of peptide remaining was quantified by measuring the peak area from the chromatograms at 220 nm and normalized to the levels detected at the 0 h time point using Agilent MassHunter Qualitative Analysis v.B.07.00. The reported values are the mean ± SD of three independent experiments.

### Peptide Stability Assays with Calu-3 Cells

Calu-3 cells were purchased from ATCC and cultured in RPMI containing 10% heat-inactivated FBS and 2 mM l-glutamine at 37 °C, 5% CO_2_. Cells were plated at 10,000 cells/well in clear 96-well plates (Corning) and grown for 24 h before treating cells with 10 μM MCPs T2-MCP-06, T2-MCP-09, and T2-MCP-19 in RPMI containing 10% heat-inactivated FBS and 2 mM l-glutamine for 72 h at 37 °C, 5% CO_2_. Medium (50 μL) was removed and quenched with 350 μL acetonitrile with 5% (v/v) formic acid (FA) and vortexed for 25 min. Samples were centrifuged at 3750 rpm for 15 min at 4 °C, and supernatants were analyzed by LC-MS/MS as described above. The percentage of intact peptide remaining was quantified by measuring the peak area from the LC-MS/MS chromatograms at 214 nm and normalized to the levels detected at the 0 h time point. Results represent the data from two independent experiments.

### Peptide Stability Assays in Human and Mouse Plasma

Human and mouse plasma were thawed and centrifuged at 3750 rpm for 15 min at 4 °C to remove clots. Plasma supernatant was adjusted to pH 7.25–7.35. The MCPs T2-MCP-06, T2-MCP-09, T2- T2-MCP-14, T2-MCP-15, and MCP-19 were added to 50 μL plasma to a final concentration of 1 μM and incubated for 0 min, 15 min, 1 h, and 4 h. At the respective time points, reactions were quenched with 350 μL acetonitrile with 5% (v/v) formic acid and vortexed for 25 min. Samples were centrifuged at 3750 rpm for 15 min at 4 °C, and supernatants were diluted with water and then analyzed by high-resolution LC-MS/MS (Thermo Fisher Scientific Inc.). The percentage of intact peptide remaining was quantified by measuring the peak area from LC-MS extracted ion chromatograms and normalized to the levels detected at the 0 h time point. Results represent the data from two independent experiments.

### Identification of T2-MCP-09 Cleavage Sites in Human and Mouse Plasma

The cleavage sites of T2-MCP-09 were identified on the basis of metabolite formation during plasma incubation. Using the high-resolution mass spectrometry data obtained from the plasma stability assay, a major metabolite was detected in both mouse and human plasma with an exact mass (*m*/*z*) of 624.7790 and a charge state of 2, which was characterized to be a metabolite due to the loss of Arg from the parent molecule (Figure S8c,d). These results suggest that the amide bonds adjacent to Arg10 are the initial cleavage sites.

### Inhibition of Pericellular TMPRSS2 Activity by MCPs and IC_50_ Determination

Vero E6 cells (ATCC, CRL-1586) were cultured in Eagle’s minimum essential medium (EMEM) supplemented with 2 mM l-glutamine, 100 IU/ml penicillin, 100 μg/mL streptomycin, and 10% FBS (Wisent Bioproducts) at 37 °C, 5% CO_2_. Cells were seeded at 350,000 cells/well in a 12-well plate and grown for 24 h. Cells were transfected with 1000 ng of pcDNA3.1-TMPRSS2 or 1000 ng of pcDNA3.1 using Lipofectamine 3000 (Thermo Fisher Scientific) for 24 h. Cells were then washed with PBS before adding T2-MCP-06, T2-MCP-09, T2-MCP-19, or DMSO control (0.1%) in HCell-100 medium (Wisent Bioproducts) containing 200 μM Boc-QAR-AMC fluorogenic substrate. T2-MCP-06 was tested from 10 μM to 0.3 nM, and both T2-MCP-09 and T2-MCP-19 were tested from 1 μM to 0.03 nM. Following treatment for 24 h, 90 μL of cell medium was transferred to a black 96-well plate, and fluorescence was acquired at room temperature in a BioTek Synergy HTX Multimode microplate reader (Agilent, BTS1LFA), with excitation at 360 nm and emission at 460 nm. IC_50_ values were calculated by generating a nonlinear regression with variable slope (four parameters) analysis from a log­[compound] versus fluorescence data plot. For a normalized graphical representation, fluorescence data were normalized using the top (100%) and bottom (0%) parameters of the fitted curve used for IC_50_ calculation. These normalized data were then fitted through a nonlinear regression with variable slope (four parameters) analysis. Data analysis was performed using GraphPad Prism 10.6.1. Calculated IC_50_ values represent the mean ± SD of three independent experiments.

### SARS-CoV-2 δS Virus-like Particle (VLP) Production

VLPs pseudotyped with the SARS-CoV-2 spike protein delta variant were produced as previously described.^43,78^ Briefly, HEK293T/T7 cells were seeded at 6 × 10^6^ cells per T75 cell culture flask in Dulbecco’s modified Eagle’s medium (DMEM) supplemented with 2 mM l-glutamine, 100 IU/mL penicillin, 100 μg/mL streptomycin, and 10% FBS (Wisent Bioproducts) for 24 h at 37 °C, 5% CO_2_. Cells were transfected using Lipofectamine 3000 (Thermo Fisher Scientific) following the manufacturer’s instructions using the following plasmids: 8 μg of reporter gene (Luciferase-ZsGreen, NR52516), 6 μg helper plasmid (psPAX2, Addgene #12260), and 6 μg of SARS-CoV-2 spike protein (delta variant), which harbored a deletion of the 19 first amino acid for enhanced expression at cell surface.^43^ Control VLPs deprived of spike glycoprotein were produced by transfecting 6 μg of empty vector (pcDNA3.1). VLP quantification and normalization was performed by immunoblotting as previously described.^43^ For infection assays, internal normalization standard was set at 50% (v/v) of VLPs when infecting Vero E6 and Calu-3 cells.

### SARS-CoV-2 δS VLP Infectivity Assay in Vero E6-TMPRSS2 Cells

Vero E6 were seeded at 25,500 cells/well in a 96-well white cell culture-treated plates (Falcon, catalog # 353296) and grown for 24 h at 37 °C, 5% CO_2_. Cells were transfected with 40 ng of pcDNA3.1-TMPRSS2 or 40 ng pcDNA3.1 (empty vector) using Lipofectamine 3000 (Thermo Fisher Scientific) for 24 h. Prior to VLP treatment, cells were treated with 0.1, 1, 10 μM of the MCPs T2-MCP-06, T2-MCP-09, T2-MCP-19, or the control inhibitors N-0385 and camostat mesylate (B2082, APExBIO) diluted in DMSO or DMSO (0.1%) control in serum-free EMEM for 1 h. Compound N-0385 was kindly gifted by Pr. Pierre-Luc Boudreault (Université de Sherbrooke) and was synthesized as previously described.^31^ Following compound treatment, a normalized preparation of VLPs mixed with respective concentrations of inhibitors or DMSO in DMEM containing 10% FBS were then added to wells (5% FBS final in each well). After incubation for 24 h, luminescence was measured using One-Glo-EX luciferase assay reagent (Promega). Readings were taken at room temperature, with a counting time of 1 s using a Berthold TriStar2 LB 942 microplate reader and ICE program version 1.0.9.8. Statistical analyses were carried out by a Welch’s *t* test.

### SARS-CoV-2 δS VLP Infectivity Assay in Calu-3 Cells

Calu-3 cells were cultured in EMEM supplemented with 2 mM l-glutamine, 100 IU/ml penicillin, 100 μg/mL streptomycin, and 10% FBS (Wisent Bioproducts) and were seeded at 10,000 cells/well in 96-well white cell culture-treated plates (Falcon, catalog # 353296) and incubated for 24 h at 37 °C, 5% CO_2_. Prior to VLP infection, cells were treated with 0.1, 1 μM of the MCPs T2-MCP-06, T2-MCP-09, T2-MCP-19, or the control inhibitors N-0385 and camostat mesylate (B2082, APExBIO) diluted in DMSO or DMSO (0.1%) in serum-free medium for 1 h. Following compound treatment, a normalized preparation of VLPs mixed with indicated concentrations of inhibitors or DMSO in medium containing 10% FBS were added to wells (5% FBS final in each well). Final concentrations of 0.1% DMSO in compound-treated and control conditions were kept throughout the experiment. After 72 h of infection, luminescence was detected using One-Glo-EX luciferase assay reagent (Promega). Detected luminescence signals were subtracted from the background (infection with VLPs lacking spike glycoprotein) and are presented as the percentage of residual entry compared to a vehicle-treated condition (0.1% DMSO). Luminescence readings were acquired identically as in the infectivity assays in Vero E6-TMPRSS2 cells. Statistical analyses were carried out by a Welch’s *t* test.

### Solid-Phase Peptide Synthesis of Selected Macrocyclic Peptides

All peptides were synthesized using standard 9-fluorenylmethoxycarbonyl (Fmoc)-based solid-phase peptide synthesis using standard protocols. Briefly, peptides were assembled using the rink amide MBHA resin at a 0.36 mmol scale. Amino acid couplings were performed using Fmoc-protected amino acids (3.0 equiv), *N,N*-diisopropylethylamine (DIPEA) (6.0 equiv), and hexafluorophosphate azabenzotriazole tetramethyl uronium (HATU) (2.85 equiv) in *N,N*-dimethylformamide (DMF) for 1 h at room temperature. Fmoc-protecting groups were removed using 20% (v/v) 4-methylpiperidine in DMF for 20 min, and the resin was subsequently washed with DMF. For the synthesis of thioether-cyclized peptides, the final Fmoc-protecting group was removed, and peptide-resin (0.7 g, 0.36 mmol, 1.0 equiv) was mixed with chloroacetic anhydride (0.65 g, 3.6 mmol, 10.0 equiv) and DIPEA (0.63 mL, 3.6 mmol, 10.0 equiv) in 20 mL DMF and left overnight at room temperature. A ninhydrin test was performed to monitor reaction completion. Peptide cleavage from the resin was performed using 15 mL trifluoroacetic acid (TFA)/phenol/water/thioanisole/triisopropylsilane (v/w/v/v/v 90:2.5:1.5:3.5:2.5) for 3 h at room temperature. Peptides were precipitated using anhydrous diethyl ether, centrifuged, and dried in vacuo. Peptide cyclization via thioether bond formation was performed in 50% (v/v) acetonitrile in water at a concentration of 10 mg/mL, and the pH was adjusted to 8–9 by dropwise addition of DIPEA. The reaction was stirred overnight, and reaction progress was monitored using liquid chromatography–mass spectrometry (LC-MS). Following reaction completion, crude cyclic peptides were dried in vacuo. The crude cyclic peptides were purified by reverse-phase high-performance liquid chromatography (RP-HPLC, C18 column, 21 mm × 250 mm, 5 μm, 100 Å, 10 mL/min, 60 min gradient from 30 to 60% (v/v) acetonitrile in water containing 0.1% (v/v) TFA). All peptides were purified to >95% as determined by analytical LC-MS using a Agilent 1260 Infinity II liquid chromatograph coupled to an Agilent 6230 TOF MS (0.7 mL min–1 flow rate, gradient elution from 15 to 85% ACN over 4 min) using an Agilent PLRP-S polymeric reversed-phase analytical column (50 mm × 2.1 mm ID, Part No. PL1912–1502) (Figure S13 and Table S3).

### Synthesis of Activity-Based Probe PK401-cmk

PK401-cmk (Ac-hCha-Phe­(guan)-Oic-Arg-cmk) was synthesized by condensation of Ac-hCha-Phe­(guan­(Boc_2_))-Oic–OH with H-Arg­(Mtr)-cmk, followed by the removal of the protecting groups. H-Arg­(Mtr)-cmk was synthesized using a literature method.^79^ Ac-hCha-Phe­(guan­(Boc_2_))-Oic–OH was synthesized on 2-chlorotrityl resin using standard solid-phase Fmoc chemistry. Upon completion of peptide assembly, the resin was treated with 30% (v/v) 1,1,1,3,3,3-hexafluoro-2-propanol, (HFIP)/DCM (20 mL) at room temperature for 1 h, followed by filtration. The filtrate was concentrated on a rotary evaporator to dryness to afford the crude Ac-hCha-Phe­(guan­(Boc_2_))-Oic–OH. A solution of Ac-hCha-Phe­(guan­(Boc_2_))-Oic–OH (78 mg, 0.10 mmol), HOBt (16 mg, 0.12 mmol), and EDC (23 mg, 0.12 mmol) in DMF (1.5 mL) was stirred at room temperature for 30 min, followed by the addition of DIPEA (52 μL, 0.30 mmol) and H-Arg­(Mtr)-cmk.HCl (53 mg, 0.12 mmol). The reaction was stirred at room temperature for 2 h and then quenched by the addition of water (6 mL). The resulting mixture was filtered. The filter cake was washed with water (1 mL × 3 mL) and dried in vacuo. The obtained off-white solid was dissolved in a mixture of TFA (1.9 mL), TIS (50 μL), and water (50 μL). The resulting solution was shaken at room temperature for 2 h and then diluted with ether (25 mL). The resulting suspension was allowed to stand at 0 °C for 30 min, followed by centrifugation. The white precipitate was washed with ether (10 mL × 3 mL) and air-dried. The crude peptide was purified with RP-HPLC (C18 column, 21 mm × 250 mm, 5 μm, 100 Å, 10 mL/min, 60 min gradient from 30 to 60% (v/v) acetonitrile containing 0.1% (v/v) TFA). PK401-cmk was obtained as a white solid (27 mg, yield 27%).

## Supplementary Material





## Notes

Molecular formula strings list of compounds with their SMILES codes (CSV)
Kinetic and selection data and compound LC traces (Figures S1–13) (Tables S1–S3) (PDF)

## Abbreviations

(HFIP): 1,1,1,3,3,3-hexafluoro-2-propanol
(Fmoc): 9-fluorenylmethoxycarbonyl
(αMeR): α-methylated arginine
(ABP): activity-based probe
(ACE2): angiotensin-converting enzyme 2
(CHO): Chinese hamster ovary
(ClAcF): chloroacetyl-l-phenylalanine
(cmk): chloromethyl ketone
(Cit): citrulline
(r): d-Arg
(δS): delta variant of the SARS-CoV-2 spike protein
(DMEM): Dulbecco’s modified Eagle’s medium
(EMEM): Eagle’s minimum essential medium
(ESI-MS): electrospray ionization mass spectrometry
(EK): enterokinase
(FA): formic acid
(HATU): hexafluorophosphate azabenzotriazole tetramethyl uronium
(Hi5): High Five cells
(HKU1-S-RBD): HKU1 coronavirus spike protein
(hR): homo-Arg
(TMPRSS2^S441A^): recombinant human TMPRSS2 with catalytic serine substituted with an alanine
(TMPRSS2^ABP^): recombinant human TMPRSS2 with the catalytic site covalently blocked by an activity-based probe
(LDLR-A): LDL receptor type A
(LC-MS): liquid chromatography–mass spectrometry
(MCPs): macrocyclic peptides
(MeC): *N*-methyl Cys
(MeR): *N*α-methyl Arg
(RMe): *N*ω-methyl Arg
(DIPEA): *N*,*N*-diisopropylethylamine
(DMF): *N*,*N*-dimethylformamide
(RP-HPLC): reverse-phase high-performance liquid chromatography
(SRCR): scavenger receptor cysteine-rich
(SEC): size exclusion chromatography
(SPR): surface plasmon resonance
(TFA): trifluoroacetic acid
(TTSPs): type II transmembrane proteases
(TMPRSS2): transmembrane serine protease 2
(VOCs): variants of concern
(*T. ni*): *Trichoplusia ni*
(Vero E6-TMPRSS2): Vero E6 cells transiently expressing- wild type human TMPRSS2
(VLPs): virus-like particles

## References

[1] Bugge T. H., Antalis T. M., Wu Q. (2009). Type II transmembrane serine proteases. J. Biol. Chem..

[2] Tanabe L. M., List K. (2017). The role of type II transmembrane serine protease-mediated signaling
in cancer. FEBS J..

[3] Szabo R., Bugge T. H. (2011). Membrane-Anchored
Serine Proteases in Vertebrate Cell
and Developmental Biology*. Ann. Rev. Cell Dev.
Biol..

[4] Hoffmann M., Kleine-Weber H., Schroeder S. (2020). SARS-CoV-2 Cell Entry
Depends on ACE2 and TMPRSS2 and Is Blocked by a Clinically Proven
Protease Inhibitor. Cell.

[5] Limburg H., Harbig A., Bestle D. (2019). TMPRSS2 Is the Major
Activating Protease of Influenza A Virus in Primary Human Airway Cells
and Influenza B Virus in Human Type II Pneumocytes. J. Virol..

[6] Shen L. W., Mao H. J., Wu Y. L., Tanaka Y., Zhang W. (2017). TMPRSS2: A
potential target for treatment of influenza virus and coronavirus
infections. Biochimie.

[7] Matsuyama S., Nao N., Shirato K. (2020). Enhanced isolation of SARS-CoV-2 by TMPRSS2-expressing
cells. Proc. Natl. Acad. Sci. U.S.A..

[8] Bestle D., Heindl M. R., Limburg H. (2020). TMPRSS2 and furin are
both essential for proteolytic activation of SARS-CoV-2 in human airway
cells. Life Sci. Alliance.

[9] Han Y., Ma Y., Wang Z. (2024). TMPRSS13 promotes the cell entry of swine acute
diarrhea syndrome coronavirus. J. Med. Virol..

[10] Paoloni-Giacobino A., Chen H., Peitsch M. C., Rossier C., Antonarakis S. E. (1997). Cloning
of the TMPRSS2 Gene, Which Encodes a Novel Serine Protease with Transmembrane,
LDLRA, and SRCR Domains and Maps to 21q22.3. Genomics.

[11] Afar D. E., Vivanco I., Hubert R. (2001). Catalytic cleavage of
the androgen-regulated TMPRSS2 protease results in its secretion by
prostate and prostate cancer epithelia. Cancer
Res..

[12] Lubinski B., Whittaker G. R. (2024). Host Cell
Proteases Involved in Human Respiratory Viral
Infections and Their Inhibitors: A Review. Viruses.

[13] Fraser B. J., Beldar S., Seitova A. (2022). Structure and activity
of human TMPRSS2 protease implicated in SARS-CoV-2 activation. Nat. Chem. Biol..

[14] Végh B., Kocsis A., Dobó J. (2025). Efficient production
of fully active, SARS-CoV-2-priming, wildtype TMPRSS2 ectodomain via
co-expression of HAI-2 allows for both auto- and cross-activation
mechanisms. Biochem. J..

[15] Jackson C. B., Farzan M., Chen B., Choe H. (2022). Mechanisms of SARS-CoV-2
entry into cells. Nat. Rev. Mol. Cell Biol..

[16] Kishimoto M., Uemura K., Sanaki T. (2021). TMPRSS11D and TMPRSS13
Activate the SARS-CoV-2 Spike Protein. Viruses.

[17] Chan J. F.-W., Huang X., Hu B. (2023). Altered host protease
determinants for SARS-CoV-2 Omicron. Sci. Adv..

[18] Willett B. J., Grove J., MacLean O. A. (2022). SARS-CoV-2
Omicron is
an immune escape variant with an altered cell entry pathway. Nat. Microbiol..

[19] Milewska A., Cofas-Vargas L. F., Poma A. B., Pyrć K. (2025). Evolution
of the SARS-CoV-2 spike protein in utilizing host transmembrane serine
proteases. iScience.

[20] Iwata-Yoshikawa N., Kakizaki M., Shiwa-Sudo N. (2022). Essential role of TMPRSS2
in SARS-CoV-2 infection in murine airways. Nat.
Commun..

[21] Metzdorf K., Jacobsen H., Greweling-Pils M. C. (2023). TMPRSS2 Is Essential
for SARS-CoV-2 Beta and Omicron Infection. Viruses.

[22] Saunders N., Fernandez I., Planchais C. (2023). TMPRSS2 is a functional
receptor for human coronavirus HKU1. Nature.

[23] McCallum M., Park Y. J., Stewart C. (2024). Human coronavirus HKU1
recognition of the TMPRSS2 host receptor. Cell.

[24] Fernández I., Saunders N., Duquerroy S. (2024). Structural basis of
TMPRSS2 zymogen activation and recognition by the HKU1 seasonal coronavirus. Cell.

[25] Wang H., Liu X., Zhang X. (2024). TMPRSS2 and glycan receptors synergistically
facilitate coronavirus entry. Cell.

[26] Carabelli A. M., Peacock T. P., Thorne L. G. (2023). SARS-CoV-2 variant biology:
immune escape, transmission and fitness. Nat.
Rev. Microbiol..

[27] Duan Y., Zhou H., Liu X. (2023). Molecular mechanisms
of SARS-CoV-2 resistance to nirmatrelvir. Nature.

[28] Iketani S., Mohri H., Culbertson B. (2023). Multiple pathways for
SARS-CoV-2 resistance to nirmatrelvir. Nature.

[29] Stevens L. J., Pruijssers A. J., Lee H. W. (2022). Mutations in the SARS-CoV-2
RNA-dependent RNA polymerase confer resistance to remdesivir by distinct
mechanisms. Sci. Transl. Med..

[30] Gandhi S., Klein J., Robertson A. J. (2022). De novo emergence of
a remdesivir resistance mutation during treatment of persistent SARS-CoV-2
infection in an immunocompromised patient: a case report. Nat. Commun..

[31] Shapira T., Monreal I. A., Dion S. P. (2022). A TMPRSS2 inhibitor
acts as a pan-SARS-CoV-2 prophylactic and therapeutic. Nature.

[32] Damalanka V. C., Banas V., De Bona P. (2024). Mechanism-Based
Macrocyclic
Inhibitors of Serine Proteases. J. Med. Chem..

[33] Hoffmann M., Schroeder S., Kleine-Weber H. (2020). Nafamostat Mesylate
Blocks Activation of SARS-CoV-2: New Treatment Option for COVID-19. Antimicrob. Agents Chemother..

[34] Mahoney M., Damalanka V. C., Tartell M. A. (2021). A novel class of TMPRSS2
inhibitors potently block SARS-CoV-2 and MERS-CoV viral entry and
protect human epithelial lung cells. Proc. Natl.
Acad. Sci. U.S.A..

[35] Wang H., Yang Q., Liu X. (2023). Structure-based discovery
of dual pathway inhibitors for SARS-CoV-2 entry. Nat. Commun..

[36] Lemieux G., Pérez-Vargas J., Désilets A. (2025). From N-0385 to N-0920:
Unveiling a Host-Directed Protease Inhibitor with Picomolar Antiviral
Efficacy against Prevalent SARS-CoV-2 Variants. J. Med. Chem..

[37] Ferková S., Lepage M., Désilets A. (2025). Optimizing the pharmacokinetics
and selectivity of TMPRSS2 inhibitors. Eur.
J. Med. Chem..

[38] Wettstein L., Knaff P. M., Kersten C. (2022). Peptidomimetic
inhibitors
of TMPRSS2 block SARS-CoV-2 infection in cell culture. Commun. Biol..

[39] Banas V., Seehra K., Mahoney M. (2026). Inhibitors of membrane
associated serine proteases block replication of coronavirus SARS-CoV-2
and influenza virus H1N1. Eur. J. Med. Chem..

[40] Kim Y. S., Jeon S. H., Kim J. (2023). A Double-Blind, Randomized,
Placebo-Controlled, Phase II Clinical Study To Evaluate the Efficacy
and Safety of Camostat Mesylate (DWJ1248) in Adult Patients with Mild
to Moderate COVID-19. Antimicrob. Agents Chemother..

[41] Morpeth S. C., Venkatesh B., Totterdell J. A. (2023). A Randomized Trial of
Nafamostat for Covid-19. NEJM Evid..

[42] Hernández-Mitre M. P., Morpeth S. C., Venkatesh B. (2024). TMPRSS2 inhibitors for
the treatment of COVID-19 in adults: a systematic review and meta-analysis
of randomized clinical trials of nafamostat and camostat mesylate. Clin. Microbiol. Infect..

[43] Pérez-Vargas J., Lemieux G., Thompson C. A. (2024). Nanomolar anti-SARS-CoV-2
Omicron activity of the host-directed TMPRSS2 inhibitor N-0385 and
synergistic action with direct-acting antivirals. Antiviral Res..

[44] Zhao Z., Yang Q., Liu X. (2025). The crystal structure
of coronavirus RBD-TMPRSS2 complex provides basis for the discovery
of therapeutic antibodies. Nat. Commun..

[45] Vinogradov A. A., Yin Y., Suga H. (2019). Macrocyclic
Peptides as Drug Candidates: Recent Progress
and Remaining Challenges. J. Am. Chem. Soc..

[46] Colas K., Bindl D., Suga H. (2024). Selection
of Nucleotide-Encoded Mass
Libraries of Macrocyclic Peptides for Inaccessible Drug Targets. Chem. Rev..

[47] Goto Y., Katoh T., Suga H. (2011). Flexizymes for genetic
code reprogramming. Nat. Protoc..

[48] Ford D. J., Duggan N. M., Fry S. E. (2021). Potent Cyclic Peptide
Inhibitors of FXIIa Discovered by mRNA Display with Genetic Code Reprogramming. J. Med. Chem..

[49] Katoh T., Sengoku T., Hirata K., Ogata K., Suga H. (2020). Ribosomal
synthesis and de novo discovery of bioactive foldamer peptides containing
cyclic β-amino acids. Nat. Chem..

[50] Nitsche C., Passioura T., Varava P. (2019). De Novo Discovery of
Nonstandard Macrocyclic Peptides as Noncompetitive Inhibitors of the
Zika Virus NS2B-NS3 Protease. ACS Med. Chem.
Lett..

[51] Norman A., Franck C., Christie M. (2021). Discovery of Cyclic
Peptide Ligands to the SARS-CoV-2 Spike Protein Using mRNA Display. ACS Cent. Sci..

[52] Bedding M. J., Franck C., Johansen-Leete J. (2024). Discovery of High Affinity
Cyclic Peptide Ligands for Human ACE2 with SARS-CoV-2 Entry Inhibitory
Activity. ACS Chem. Biol..

[53] Johansen-Leete J., Ullrich S., Fry S. E. (2022). Antiviral cyclic peptides
targeting the main protease of SARS-CoV-2. Chem.
Sci..

[54] Tan Y., Yang J., Wang M. (2024). De Novo Discovery of
a Noncovalent Cell-Penetrating Bicyclic Peptide Inhibitor Targeting
SARS-CoV-2 Main Protease. J. Med. Chem..

[55] Ye X., Ling X., Wu M. (2023). Improving Soluble Expression
of SARS-CoV-2 Spike Priming Protease TMPRSS2 with an Artificial Fusing
Protein. Int. J. Mol. Sci..

[56] Ganesan R., Zhang Y., Landgraf K. E. (2012). An allosteric anti-hepsin
antibody derived from a constrained phage display library. Protein Eng., Des. Sel..

[57] Xuan J.-A., Schneider D., Toy P. (2006). Antibodies Neutralizing
Hepsin Protease Activity Do Not Impact Cell Growth but Inhibit Invasion
of Prostate and Ovarian Tumor Cells in Culture. Cancer Res..

[58] Fraser B. J., Wilson R. P., Ferková S. (2025). Structural basis of
TMPRSS11D specificity and autocleavage activation. Nat. Commun..

[59] Kasperkiewicz P., Poreba M., Snipas S. J. (2015). Design of a Selective
Substrate and Activity Based Probe for Human Neutrophil Serine Protease
4. PLoS One.

[60] Schechter I., Berger A. (1967). On the size of the
active site in proteases. I. Papain. Biochem.
Biophys. Res. Commun..

[61] Luckett S., Garcia R. S., Barker J. J. (1999). High-resolution
structure
of a potent, cyclic proteinase inhibitor from sunflower seeds. J. Mol. Biol..

[62] de
Veer S. J., White A. M., Craik D. J. (2021). Sunflower Trypsin
Inhibitor-1 (SFTI-1): Sowing Seeds in the Fields of Chemistry and
Biology. Angew. Chem., Int. Ed..

[63] Zhao G., Yuan C., Wind T. (2007). Structural basis of
specificity of a peptidyl urokinase inhibitor, upain-1. J. Struct. Biol..

[64] Laporte M., Raeymaekers V., Van Berwaer R. (2021). The SARS-CoV-2 and other
human coronavirus spike proteins are fine-tuned towards temperature
and proteases of the human airways. PLoS Pathog..

[65] Gu X., Zheng M., Gao Y. (2025). Overview of host-directed
antiviral targets for future research and drug development. Acta Pharm. Sin. B.

[66] Kim T. S., Heinlein C., Hackman R. C., Nelson P. S. (2006). Phenotypic
Analysis
of Mice Lacking the Tmprss2-Encoded Protease. Mol. Cell. Biol..

[67] von
Delft A., Hall M. D., Kwong A. D. (2023). Accelerating
antiviral drug discovery: lessons from COVID-19. Nat. Rev. Drug Discovery.

[68] Jochmans D., Laporte M., Neyts J. (2023). Antiviral strategies
for epidemic
and pandemic preparedness. Cell Host Microbe.

[69] Martin D. E., Tripp R. A. (2024). Host-directed antiviral
therapeutics and the NIAID
research agenda: an underexplored frontier. Front. Virol..

[70] Martin V., Egelund P. H. G., Johansson H. (2020). Greening the synthesis
of peptide therapeutics: an industrial perspective. RSC Adv..

[71] Yamagishi Y., Shoji I., Miyagawa S. (2011). Natural Product-Like
Macrocyclic N-Methyl-Peptide Inhibitors against a Ubiquitin Ligase
Uncovered from a Ribosome-Expressed De Novo Library. Chem. Biol..

[72] Ishizawa T., Kawakami T., Reid P. C., Murakami H. (2013). TRAP Display: A High-Speed
Selection Method for the Generation of Functional Polypeptides. J. Am. Chem. Soc..

[73] Bashore C., Prakash S., Johnson M. C. (2023). Targeted degradation
via direct 26S proteasome recruitment. Nat.
Chem. Biol..

[74] Ganesan R., Eigenbrot C., Wu Y. (2009). Unraveling the Allosteric
Mechanism of Serine Protease Inhibition by an Antibody. Structure.

[75] Maun H. R., Liu P. S., Franke Y. (2018). Dual functionality
of
β-tryptase protomers as both proteases and cofactors in the
active tetramer. J. Biol. Chem..

[76] Lin S. J., Dong K. C., Eigenbrot C., Campagne M. v. L., Kirchhofer D. (2014). Structures
of neutrophil serine protease 4 reveal an unusual mechanism of substrate
recognition by a trypsin-fold protease. Structure.

[77] Hsieh C.-L., Goldsmith J. A., Schaub J. M. (2020). Structure-based design
of prefusion-stabilized SARS-CoV-2 spikes. Science.

[78] Crawford K. H. D., Eguia R., Dingens A. S. (2020). Protocol and Reagents
for Pseudotyping Lentiviral Particles with SARS-CoV-2 Spike Protein
for Neutralization Assays. Viruses.

[79] Sun A., Shoji M., Lu Y., Liotta D., Snyder J. (2006). Synthesis
of EF24–Tripeptide Chloromethyl Ketone: A Novel Curcumin-Related
Anticancer Drug Delivery System. J. Med. Chem..

