# Supplementary material for: Discovery of Highly Selective Noncovalent Macrocyclic Peptide Inhibitors Suppressing Both TMPRSS2 Protease Activity and Viral Receptor Function
Source: J Med Chem. 2026 Aug 12;69(17):20451–68. doi: 10.1021/acs.jmedchem.6c01094 (PMC13576450; doi:10.1021/acs.jmedchem.6c01094)
Supplement: Supplementary file 1 [file jm6c01094_si_002.pdf]

Supporting Information

Discovery of Highly Selective Noncovalent Macrocyclic Peptide Inhibitors Suppressing Both TMPRSS2 Protease Activity and Viral Receptor Function

Benjamin J. Tombling[1], Gabriel Lemieux[2], Alexandre Joushomme[2], Yuehua Wei[1], Emel Adaligil[1], Brennan Farrell[1], Steven Fleming[1], Qinying Yu[3], Bin Ma[3], Christian N. Cunningham[1], Robin Krystufek[1], Aimin Song[1], Antoine Désilets[2], Richard Leduc[2], Daniel Kirchhofer[1*]*

1 Department of Early Discovery Biochemistry, Genentech Inc., 1 DNA Way, South San Francisco, CA 94080, USA

2 Department of Pharmacology-Physiology, Faculty of Medicine and Health Sciences, Institut de Pharmacologie de Sherbrooke, Université de Sherbrooke, Sherbrooke, Québec J1H 5H3, Canada

3 Department of Drug Metabolism & Pharmacokinetics, Genentech Inc., 1 DNA Way, South San Francisco, California 94080, United States

*corresponding authors

kirchhofer.daniel@gene.com

Richard.Leduc@usherbrooke.ca

Figure S1 .... S1
Figure S2 .... S2
Figure S3 .... S4
Figure S4 .... S6
Figure S5 .... S7
Figure S6 .... S9
Figure S7 .... S10
Figure S8 .... S11
Figure S9 .... S12
Figure S10 .... S14
Figure S11 .... S15
Figure S12 .... S16
Figure S13 .... S28
Table S1 .... S29
Table S2 .... S30
Table S3 .... S31

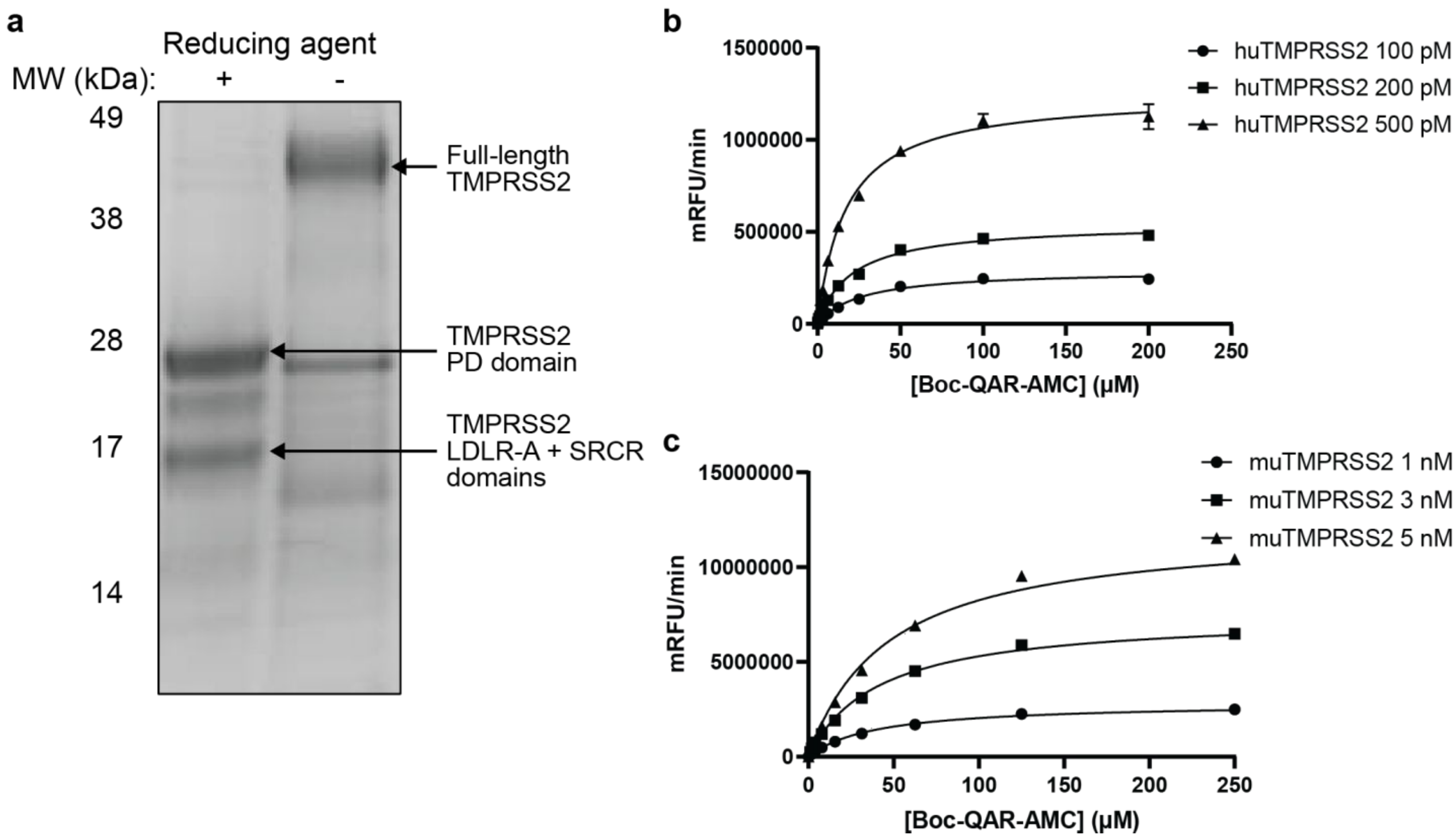

Figure S1. Characterization of engineered recombinant TMPRSS2. **a)** SDS-PAGE of TMPRSS2 under reducing (TCEP) and non-reducing conditions. TMPRSS2 bands are labeled with black arrows. PD = protease domain. **b)** Michaelis-Menten plot showing cleavage of the fluorogenic substrate Boc-QAR-AMC by human TMPRSS2. Data shown are the average ± SD of three independent experiments. **c)** Michaelis-Menten showing cleavage of the fluorogenic substrate Boc-QAR-AMC by murine TMPRSS2. Data shown are from a single experiment.

a

Codon Box	TTT	TAT	TGT	CTT	ATT	GTT	CCT	GCT	TCT	ACT	AAT	AGT	GGT	CAT	CGT	GAT	INI	TGG
	Aromatic			Aliphatic					Polar					Basic		Acidic	Cyclization	
Lib4	F	Y	W	L	I	V	P	A	S	T	N	S	G	H	R	D	ClAcF	C
Lib3	MeF	Y	hF	L	Sar	V	P	MeA	S	MeNle	N	S	G	H	R	D	ClAcF	C
Lib1	PEtG	Y	oBuG	L	iPeG	V	P	a	S	f	N	S	G	H	R	D	ClAcF	C
Lib2	MeF	Y	Cle	L	Sar	V	P	Tic	S	Bip	N	S	G	H	R	D	ClAcF	C

b

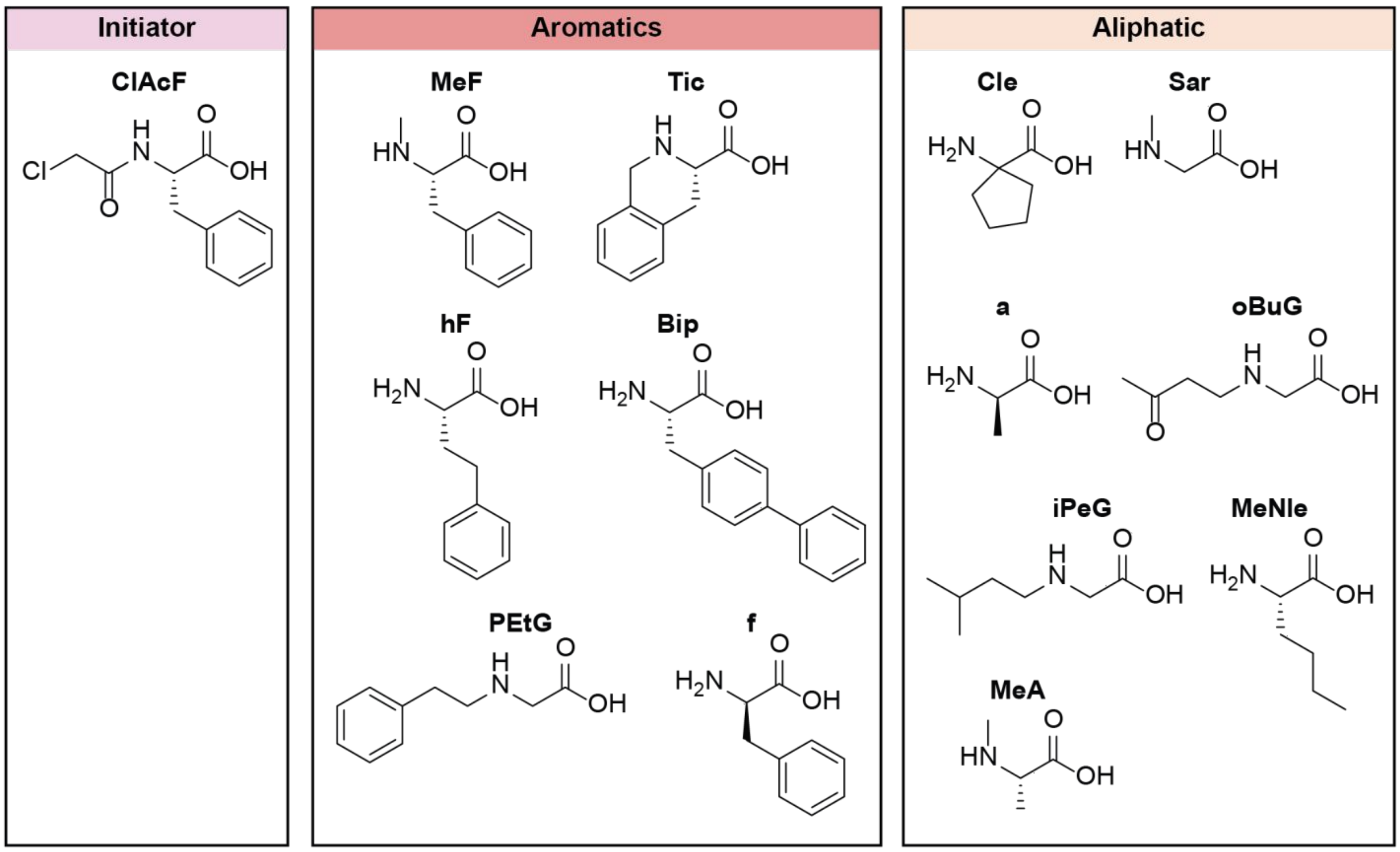

Figure S2. mRNA-display MCP library designs. **a)** Designed codon tables for mRNA display libraries highlighting the genetic code reprogramming. INI: initiator. Non-canonical amino acids are shown in red letters. For residues shown in grey letters, the same codon was used across all libraries, encoding the amino acid indicated for Lib4. **b)** Structures of the non-canonical amino acids used in the MCP library designs. ClAcF, chloroacetyl L-phenylalanine; MeF, N-methylphenylalanine; hF, homo-phenylalanine; Sar, sarcosine (N-methylglycine); MeA, N-methylalanine; MeNle, N-methylnorleucine; PEtG, N-(2-phenethyl)glycine; oBuG, 4-amino-4-oxobutanoic acid; iPeG, N-(isopentyl)glycine; Tic, 1,2,3,4-tetrahydroisoquinoline-3-carboxylic acid; Bip, biphenylalanine; f, *D*-phenylalanine; Cle, cycloleucine; a, *D*-alanine.

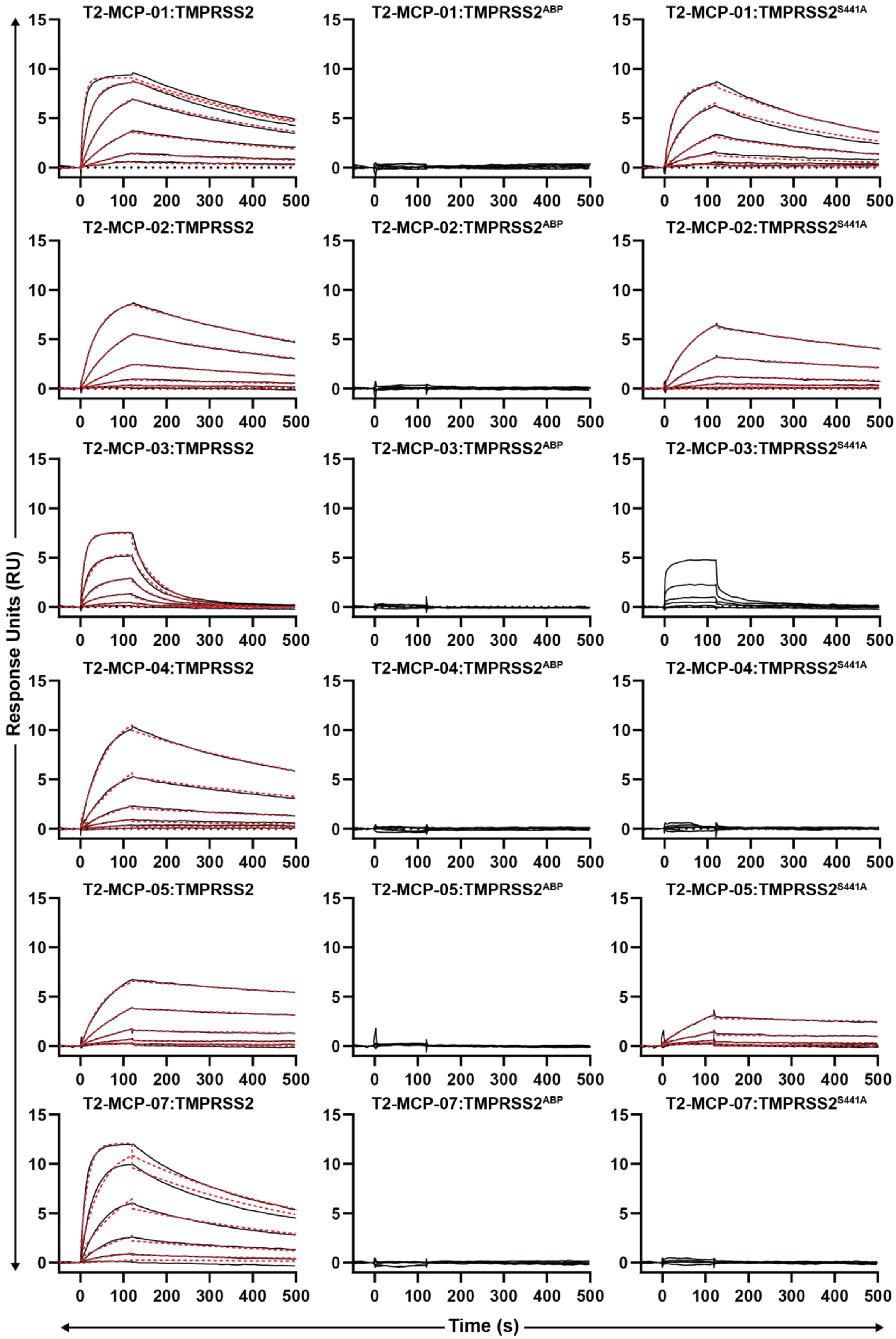
T2-MCP-01:TMPRSS2
T2-MCP-01:TMPRSS2ABP
T2-MCP-01:TMPRSS2S441A
T2-MCP-02:TMPRSS2
T2-MCP-02:TMPRSS2ABP
T2-MCP-02:TMPRSS2S441A
T2-MCP-03:TMPRSS2
T2-MCP-03:TMPRSS2ABP
T2-MCP-03:TMPRSS2S441A
T2-MCP-04:TMPRSS2
T2-MCP-04:TMPRSS2ABP
T2-MCP-04:TMPRSS2S441A
T2-MCP-05:TMPRSS2
T2-MCP-05:TMPRSS2ABP
T2-MCP-05:TMPRSS2S441A
T2-MCP-07:TMPRSS2
T2-MCP-07:TMPRSS2ABP
T2-MCP-07:TMPRSS2S441A
Response Units (RU)
Time (s)

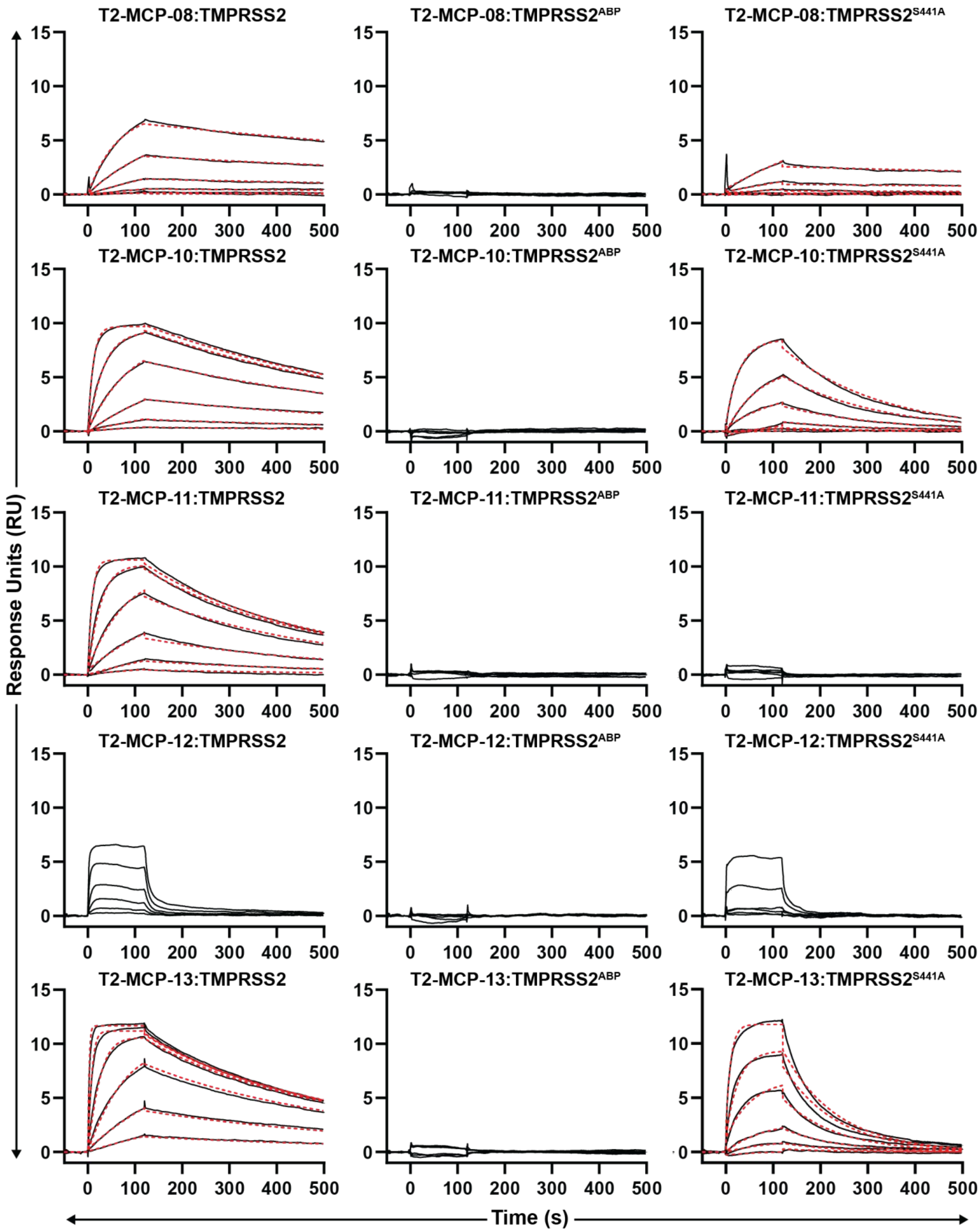

Figure S3. SPR sensorgrams showing concentration-dependent binding of MCPs to immobilized TMPRSS2, TMPRSS2^{ABP} (covalently bound activity-based probe PK401-cmk), and TMPRSS2^{S441A}

(catalytic Ser441 mutated to Ala). Peptides were injected at six serial three-fold dilutions: T2-MCP-01 1.2–300 nM, T2-MCP-02 3.7–900 nM, T2-MCP-03 3.7–900 nM, T2-MCP-04 3.7–900 nM, T2-MCP-05 3.7–900 nM, T2-MCP-07 1.2–300 nM, T2-MCP-08 3.7–900 nM, T2-MCP-10 1.2–300 nM, T2-MCP-11 3.7–900 nM, T2-MCP-12 11–2700 nM, T2-MCP-13 1.2–300 nM. Experimental curves are shown in black. Kinetic binding fits (where applicable), based on a 1:1 model, are shown in red. Kinetic constants determined from the SPR sensorgrams are shown in Table 1. Sensorgrams are representative of three independent experiments.

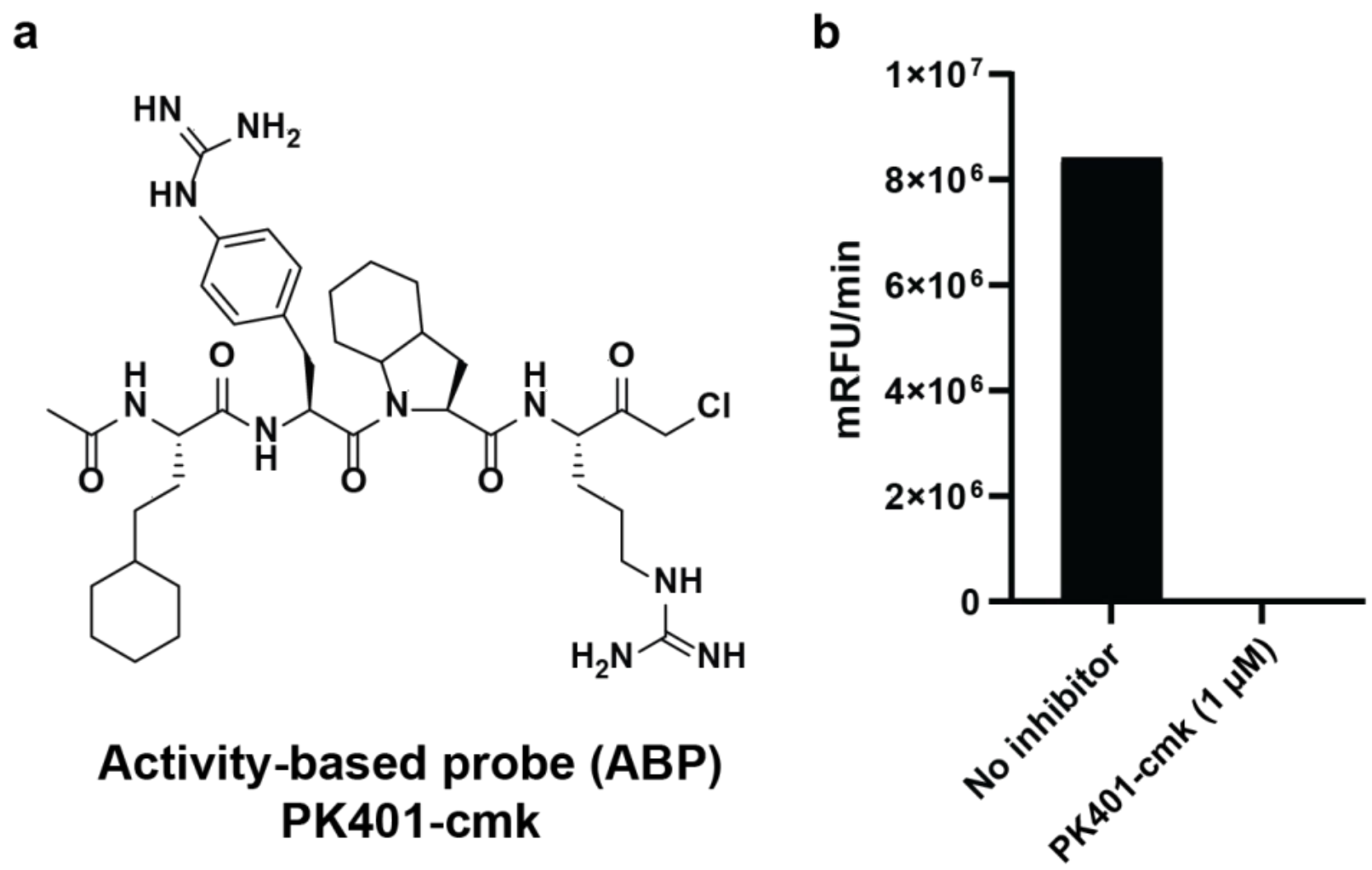

Figure S4. PK401-cmk activity-based probe. **a)** Structure of the activity-based probe Ac-hCha-Phe(guan)-Oic-Arg-chloromethylketone (PK401-cmk). **b)** A 15 min incubation of TMPRSS2 with 1 μM of PK401-cmk fully inhibits cleavage of the fluorogenic substrate Boc-QAR-AMC. Data shown are from a single experiment.

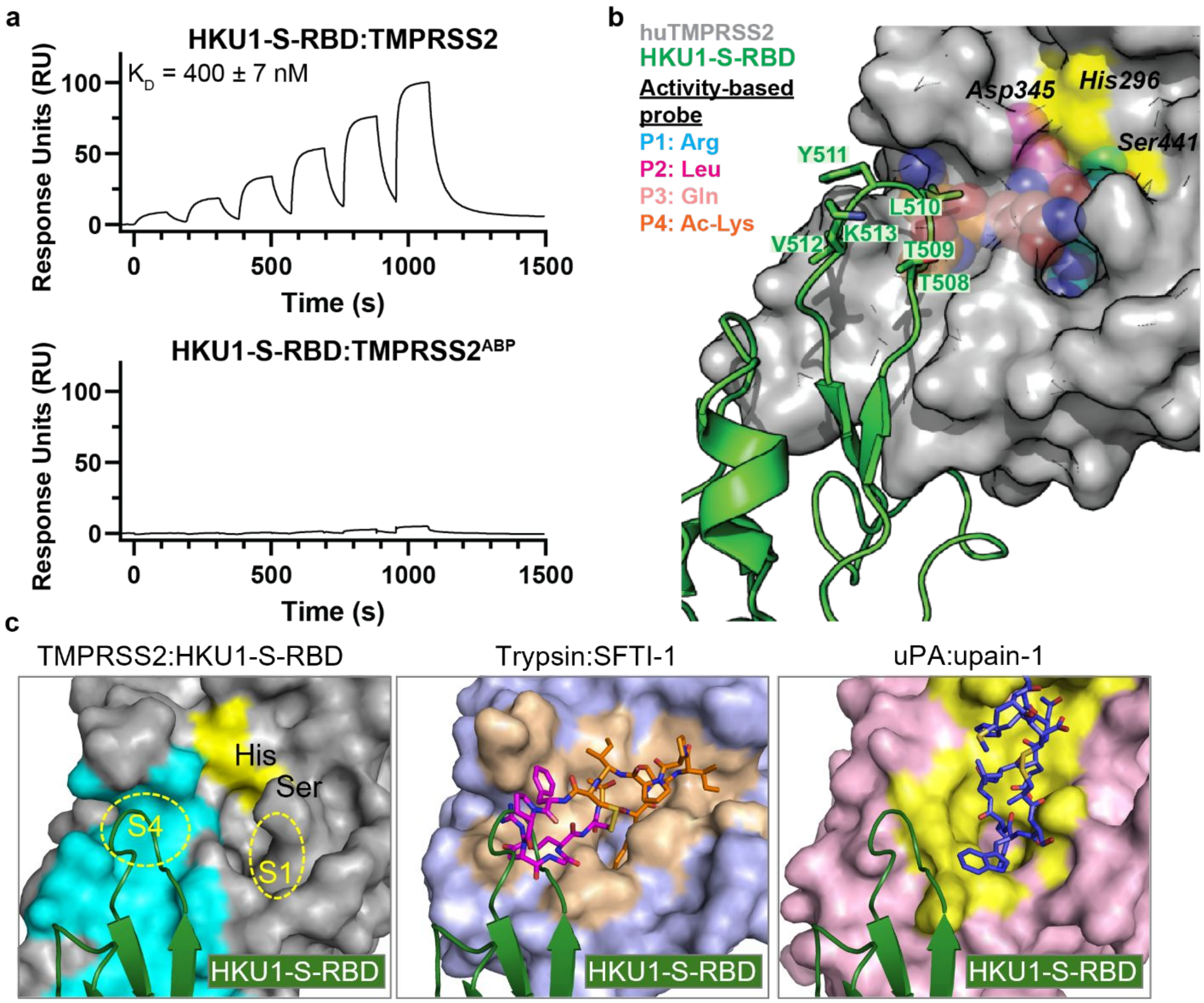

Figure S5. Structural rationale for dual inhibition of TMPRSS2 enzymatic and receptor functions. **a)** SPR sensorgrams showing concentration-dependent binding (12–3000 nM) of HKU1-S-RBD (HKU1 spike protein receptor binding domain) to immobilized TMPRSS2 (top; K_D 400 ± 7 nM) and TMPRSS2^{ABP} (bottom). Sensorgrams are representative of three independent experiments. **b)** Overlay of the structure of HKU1-S (green cartoon) bound to huTMPRSS2 (grey surface representation) (PDB 8Y8A) with hepsin in complex with the activity-based probe Ac-KQLR-cmk (PDB 1Z8G). The bound Ac-KQLR-cmk (spheres) occupies the S1–S4 subsites on the protease, in a manner similar to how PK401-cmk is expected to interact. The P4 residue of Ac-KQLR-cmk (Ac-K; orange, spheres) sterically hinders HKU1-S binding to the TMPRSS2 active site. The corresponding P4 residue of PK401 is the bulky non-canonical amino acid Ac-

hCha, which is predicted to create a similar steric clash with HKU1-S binding, thereby explaining the lack of interaction with TMPRSS2^{ABP}. **c)** Left panel: Structure of TMPRSS2:HKU1-S-RBD (PDB 8Y8A) showing the major binding loop of HKU1-S-RBD (green cartoon) interacting with TMPRSS2 (grey surface representation). The TMPRSS2 catalytic His and Ser residues are shown in yellow, and TMPRSS2 residues within 4 Å of HKU1-S-RBD are shown in cyan. The S1 and S4 subsites on TMPRSS2 are indicated by dotted circles. Center panel: Superposition of the trypsin:SFTI-1 complex (PDB 1SFI) with TMPRSS2:HKU1-S-RBD (PDB 8Y8A). Trypsin is shown as a light blue surface and trypsin residues within 4 Å of SFTI-1 (orange/magenta sticks) are shown in brown. Six SFTI-1 residues (highlighted as magenta sticks) are within 4 Å of the superimposed HKU1-S-RBD main binding loop (green cartoon), resulting in substantial steric overlap. Right panel: Superposition of the uPA:upain-1 complex (PDB 2NWN) with TMPRSS2:HKU1-S-RBD (PDB 8Y8A). UPa is shown as a pink surface and uPA residues within a 4 Å of upain-1 (blue sticks) are shown in yellow. The orientation of upain-1 remains largely confined to the active-site center and is predicted to have minimal impact on HKU1-S-RBD binding, based on the superimposed HKU1-S-RBD main binding loop (green cartoon).

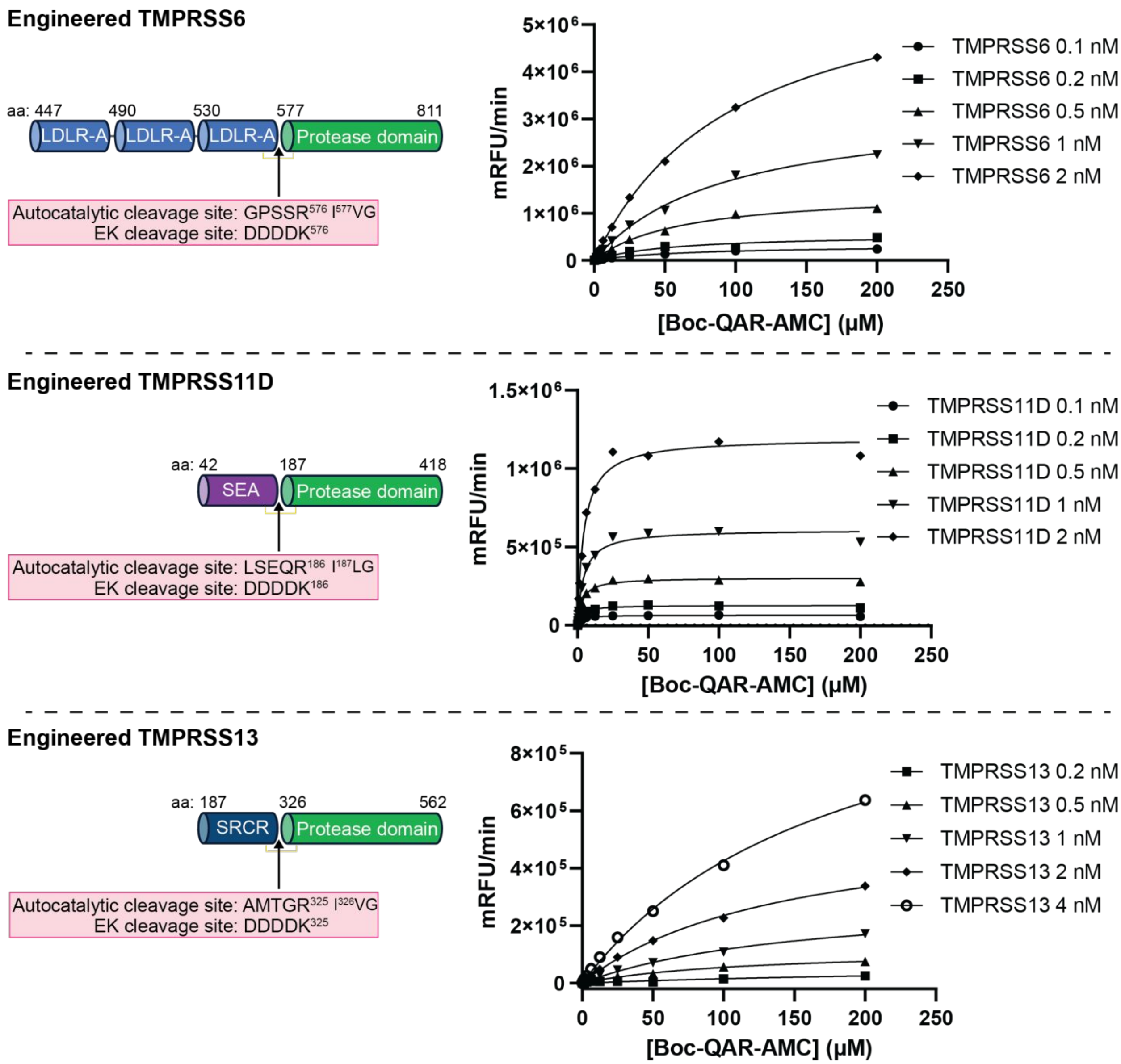

Figure S6. Design and characterization of recombinant human TTSPs using the enterokinase cleavage site sequence. Left panel: A schematic representation of the protein construct designs of TMPRSS6, TMPRSS11D and TMPRSS13 used for selectivity studies. Right panel: Michaelis-Menten plots showing cleavage of the fluorogenic substrate Boc-QAR-AMC by EK-activated TTSPs. Data shown are from a single experiment.

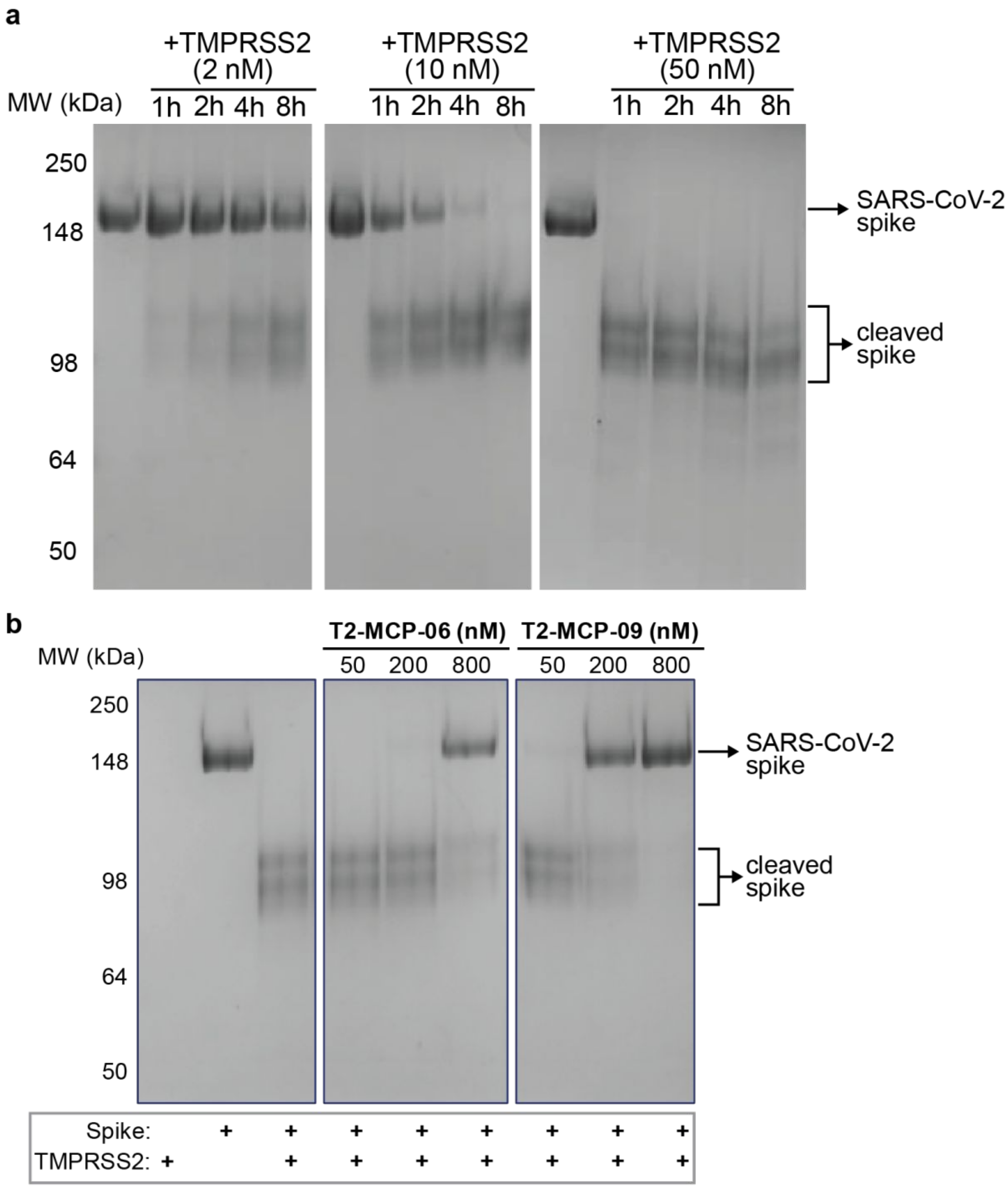

Figure S7. MCPs inhibit TMPRSS2-mediated cleavage of recombinant SARS-CoV-2 spike protein. **a)** Concentration- and time-dependent cleavage of recombinant SARS-CoV-2 spike protein by recombinant TMPRSS2. Samples were analyzed using SDS-PAGE under reducing (TCEP) conditions and proteins were stained with Coomassie blue. **b)** MCPs T2-MCP-06 and T2-MCP-09 prevent TMPRSS2 (50 nM) cleavage of SARS-CoV-2 spike protein in a concentration-dependent manner (50–800 nM). Intact SARS-CoV-2 spike protein and the TMPRSS2-mediated cleaved fragments are indicated.

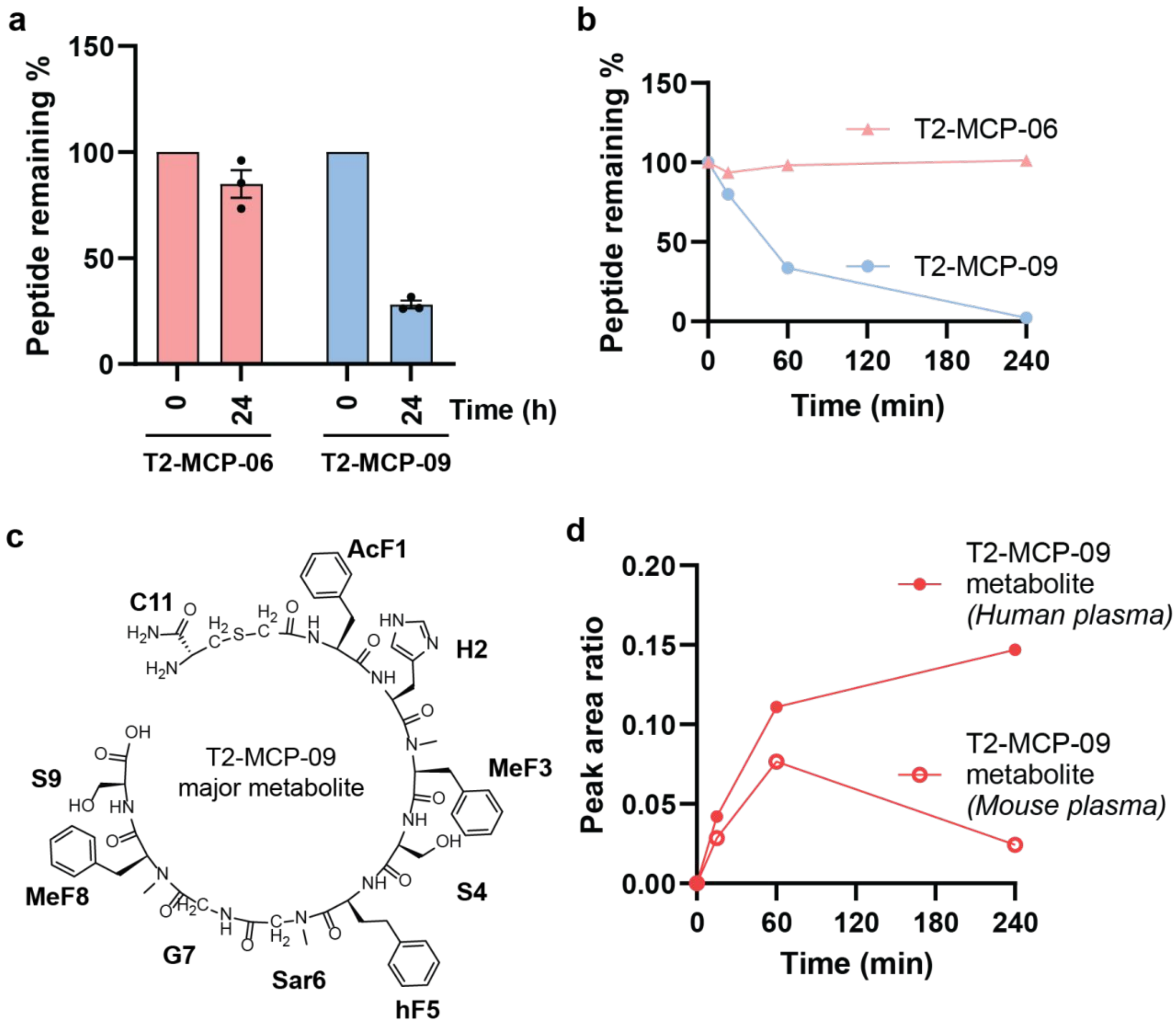

Figure S8. Characterization of T2-MCP-09 proteolytic instability. **a)** Recombinant TMPRSS2 cleaves T2-MCP-09, but not T2-MCP-06. MCPs and TMPRSS2 (1:1 molar ratio) were incubated for 24 h and the remaining intact peptides were quantified by LC-MS. Data shown are the average ± SD of three independent experiments. **b)** In vitro mouse plasma stability of T2-MCP-06 and T2-MCP-09. The remaining intact peptide was quantified by LC-MS. Data points are shown as the average of two independent experiments. **c)** Chemical structure of the major T2-MCP-09 metabolite generated by cleavage at the amide bonds adjacent of Arg10. **d)** Formation kinetics of the major T2-MCP-09 metabolite upon human and mouse plasma incubation. The metabolite was determined by LC-MS/MS. Data shown are from a single experiment.

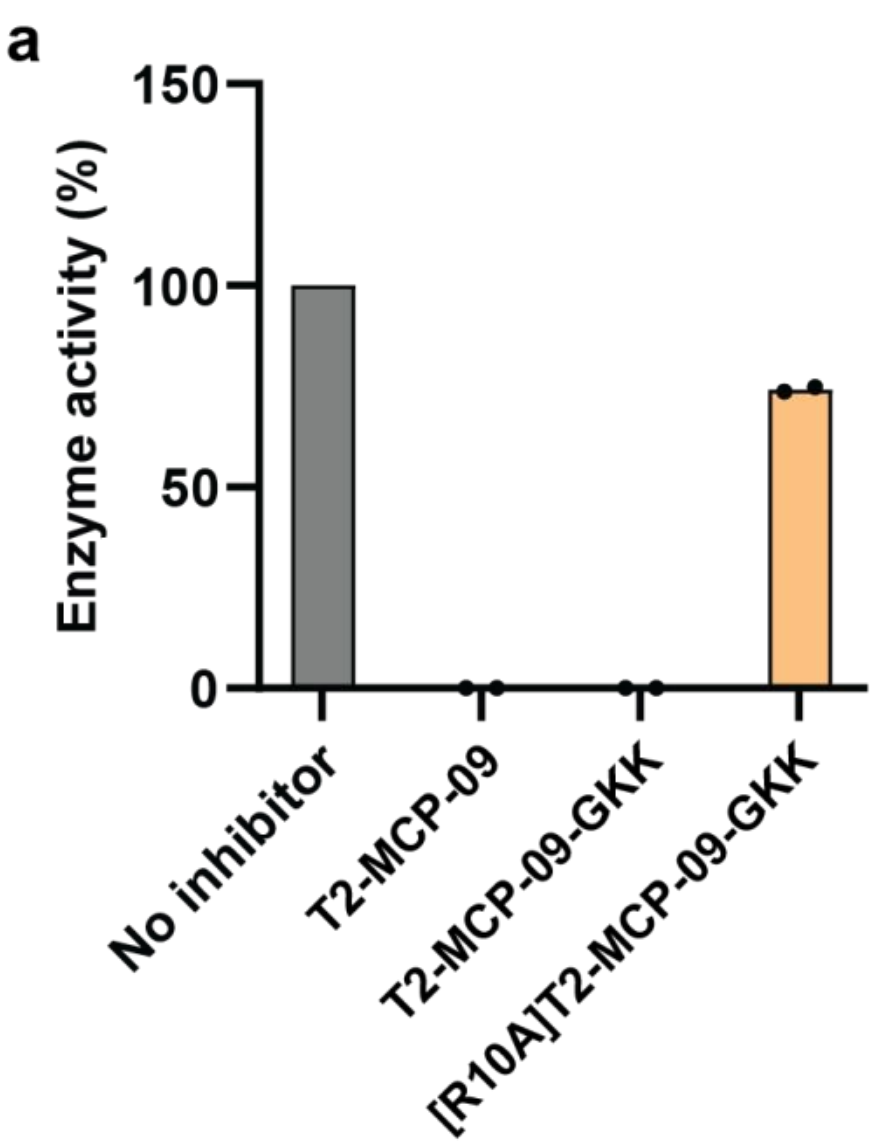

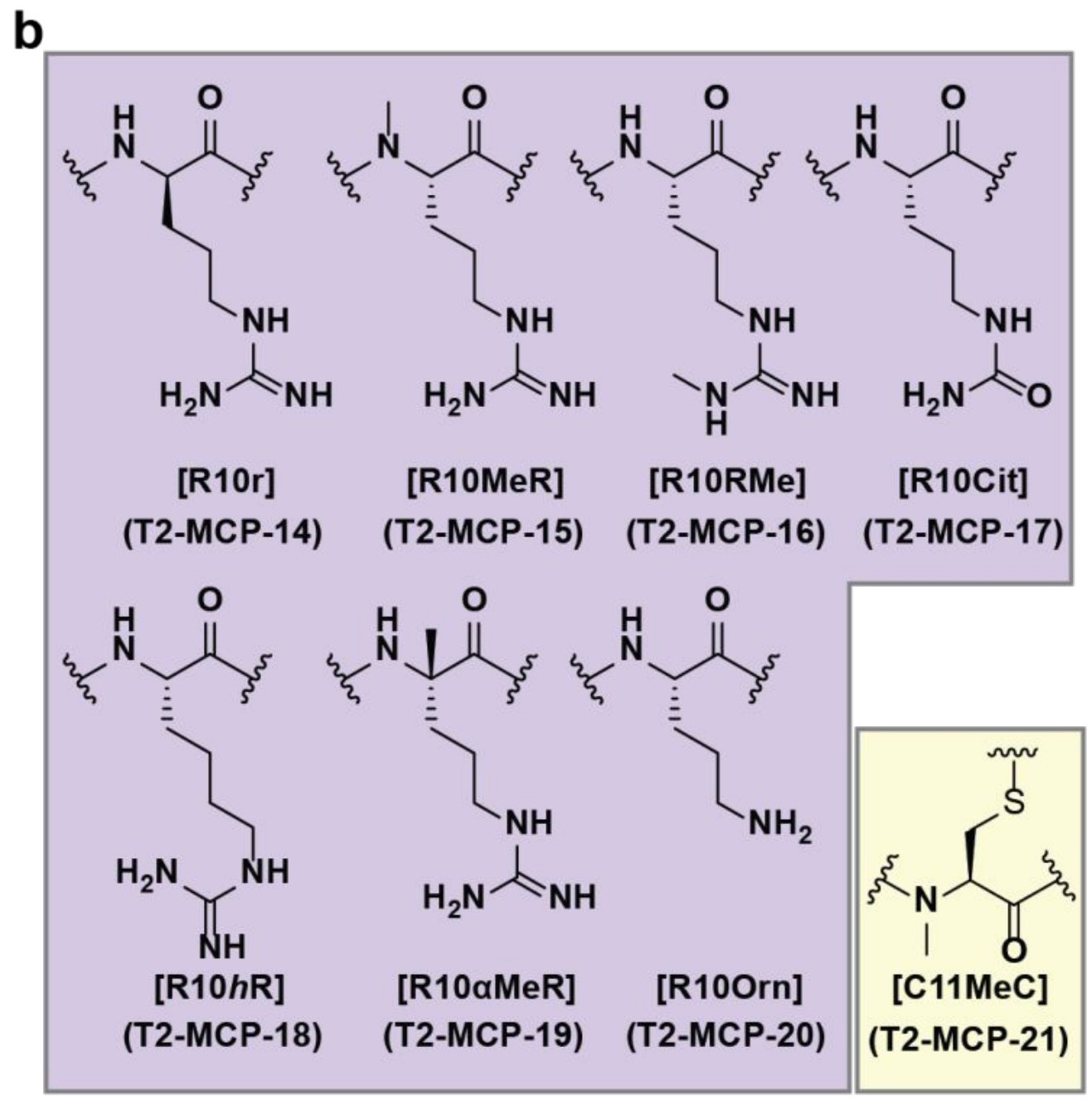

c

MCP	p10	p11
T2-MCP-09	R	C
Designed mutants		
T2-MCP-14	r	C
T2-MCP-15	MeR	C
T2-MCP-16	RMe	C
T2-MCP-17	Cit	C
T2-MCP-18	hR	C
T2-MCP-19	αMeR	C
T2-MCP-20	Orn	C
T2-MCP-21	R	MeC

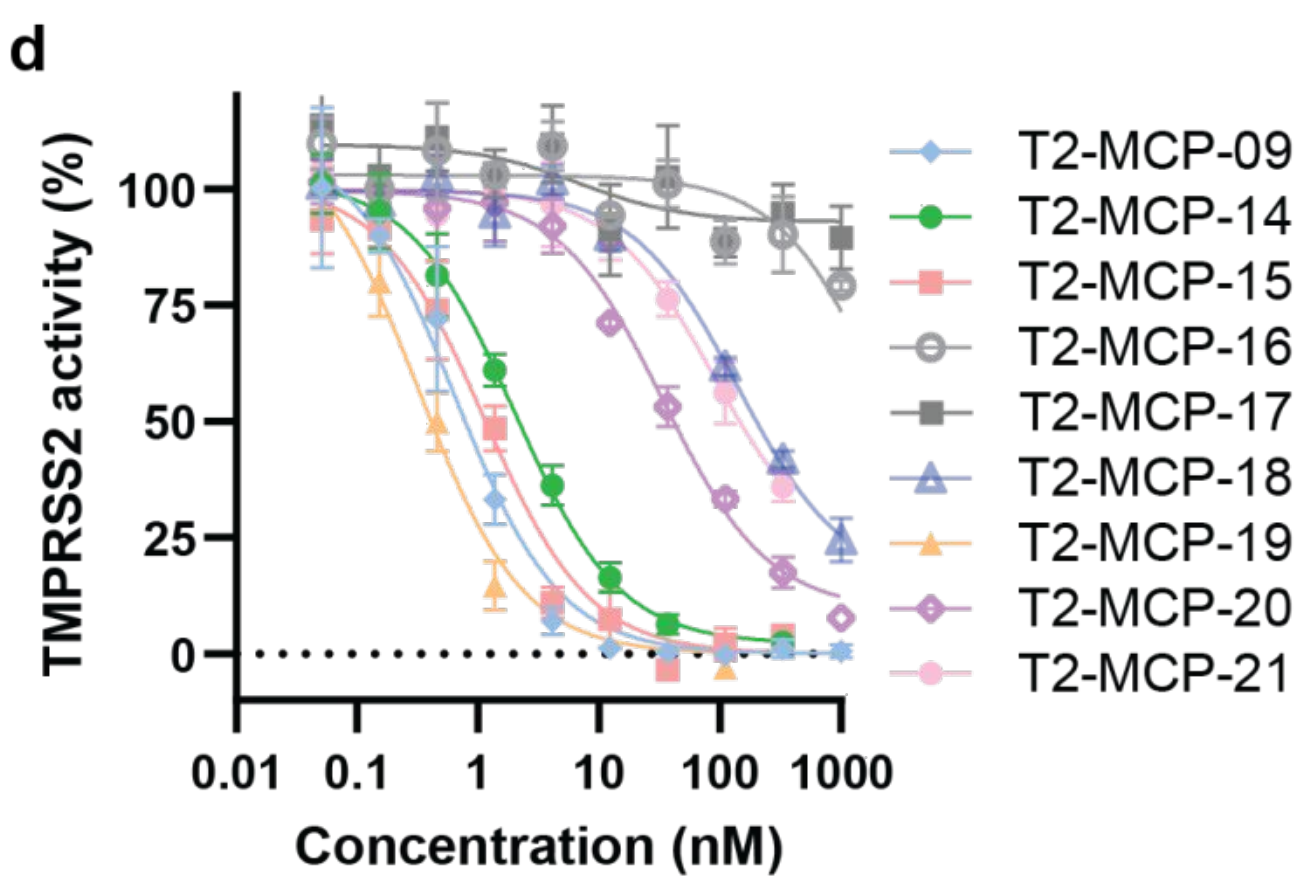

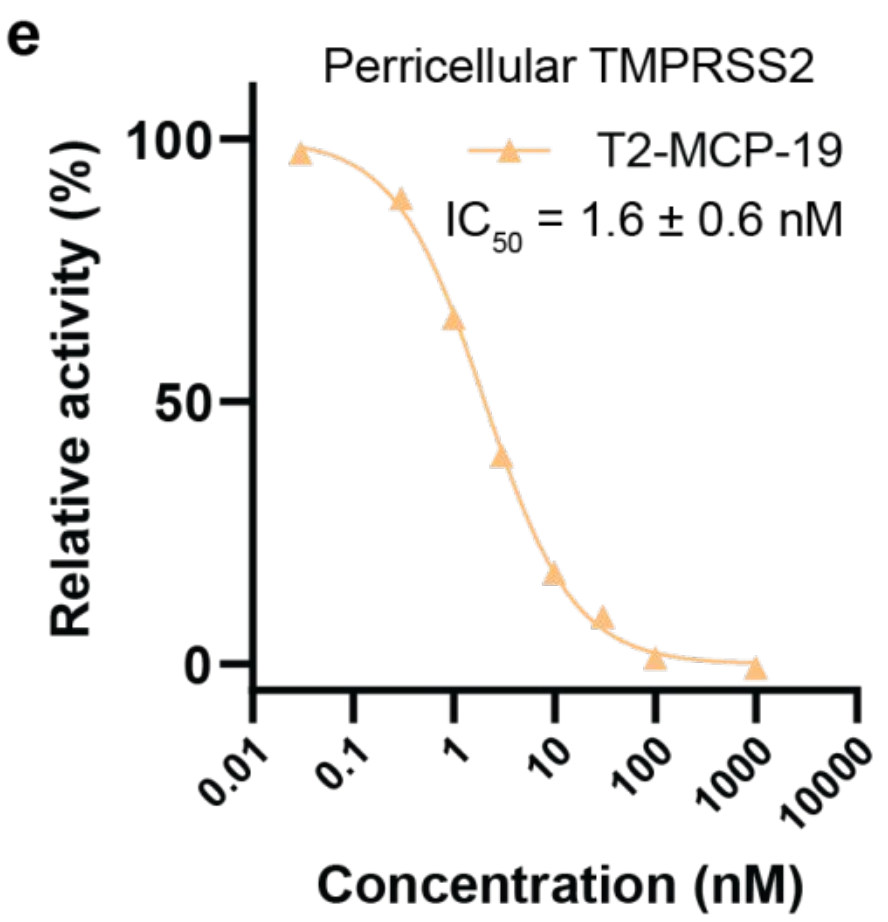

Figure S9. Rationally designed T2-MCP-09 analogues retain TMPRSS2 inhibitory activity. **a)** The Arg10 residue of T2-MCP-09 is important for TMPRSS2 inhibitory activity, as the R10A mutation abolishes

activity. "-GKK" indicates Gly-Lys-Lys which was added to the C-terminus of the peptides. All compounds were tested at 10 μM. Data points are shown as the average of two independent experiments. **b)** Chemical structures of the non-canonical amino acids used to replace Arg10 and Cys11 in T2-MCP-09. Arg10 derivatives are shown in the purple box (from T2-MCP-14 to T2-MCP-20) and the Cys11 derivative is shown in the yellow box (T2-MCP-21). **c)** A list of the designed MCPs in which residues at positions 10 and 11 of T2-MCP-09 were replaced with non-canonical amino acids. Amino acid mutations from the parent T2-MCP-09 are highlighted as red. **d)** Several of the designed MCPs strongly inhibit cleavage of fluorogenic substrate Boc-QAR-AMC by recombinant TMPRSS2. The parent T2-MCP-09 is shown for reference. IC_{50} values were calculated by generating a non-linear regression with variable slope (four parameters) analysis from a log[compound] versus normalized fluorescence data plot. Data shown are the average ± SD of three independent experiments. **e)** T2-MCP-19 inhibits pericellular cleavage of the fluorogenic substrate Boc-QAR-AMC by Vero E6 cells expressing a TMPRSS2-Myc-His construct. IC_{50} values were calculated by generating a non-linear regression with variable slope (four parameters) analysis from a log[compound] versus fluorescence data plot. A representative IC_{50} curve is shown, with the mean value ± SD of three independent experiments. Data is graphically represented by normalizing fluorescence data using the top (100%) and bottom (0%) parameters of the fitted curve used for IC_{50} calculation. These normalized data were then fitted using a four-parameter variable-slope non-linear regression.

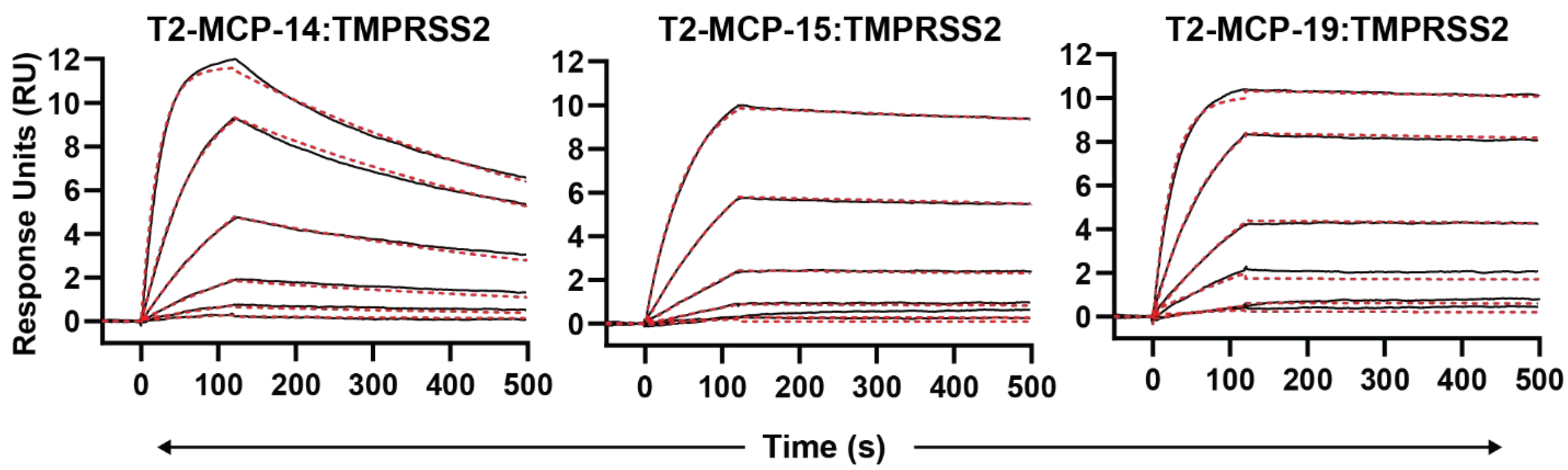

Figure S10. The rationally designed T2-MCP-09 analogues T2-MCP-14, T2-MCP-15 and T2-MCP-19 have strong binding affinity for TMPRSS2. SPR sensorgrams showing concentration-dependent binding (0.2–50 nM) of T2-MCP-14, T2-MCP-15 and T2-MCP-19 to immobilized TMPRSS2. Sensorgrams are representative of three independent experiments.

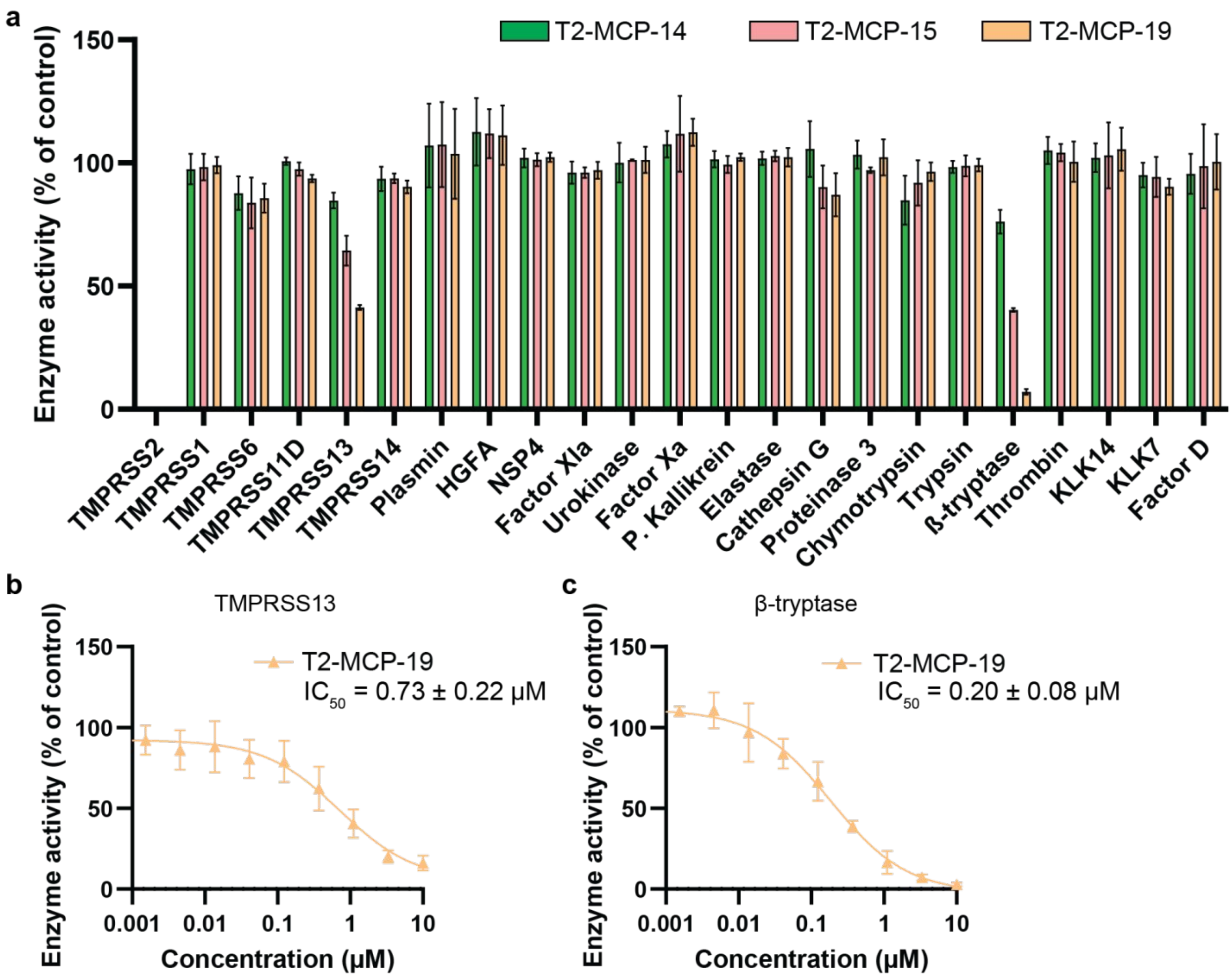

Figure S11. The rationally designed T2-MCP-09 analogues T2-MCP-14, T2-MCP-15 and T2-MCP-19 have strong selectivity for TMPRSS2 among trypsin fold serine proteases. **a)** The inhibitory activities of T2-MCP-14, T2-MCP-15 and T2-MCP-19 were tested against a panel of trypsin fold serine proteases, including several TMPRSS2-related TTSP family members. Compounds were investigated at a concentration of 1 µM. **b–c)** T2-MCP-19 inhibits the enzymatic activity of recombinant human TMPRSS13 (**b**) and human β-tryptase (**c**). IC_{50} values were calculated by generating a non-linear regression with variable slope (four parameters) analysis from a log[compound] versus normalized fluorescence data plot. Data shown are the average ± SD of three independent experiments.

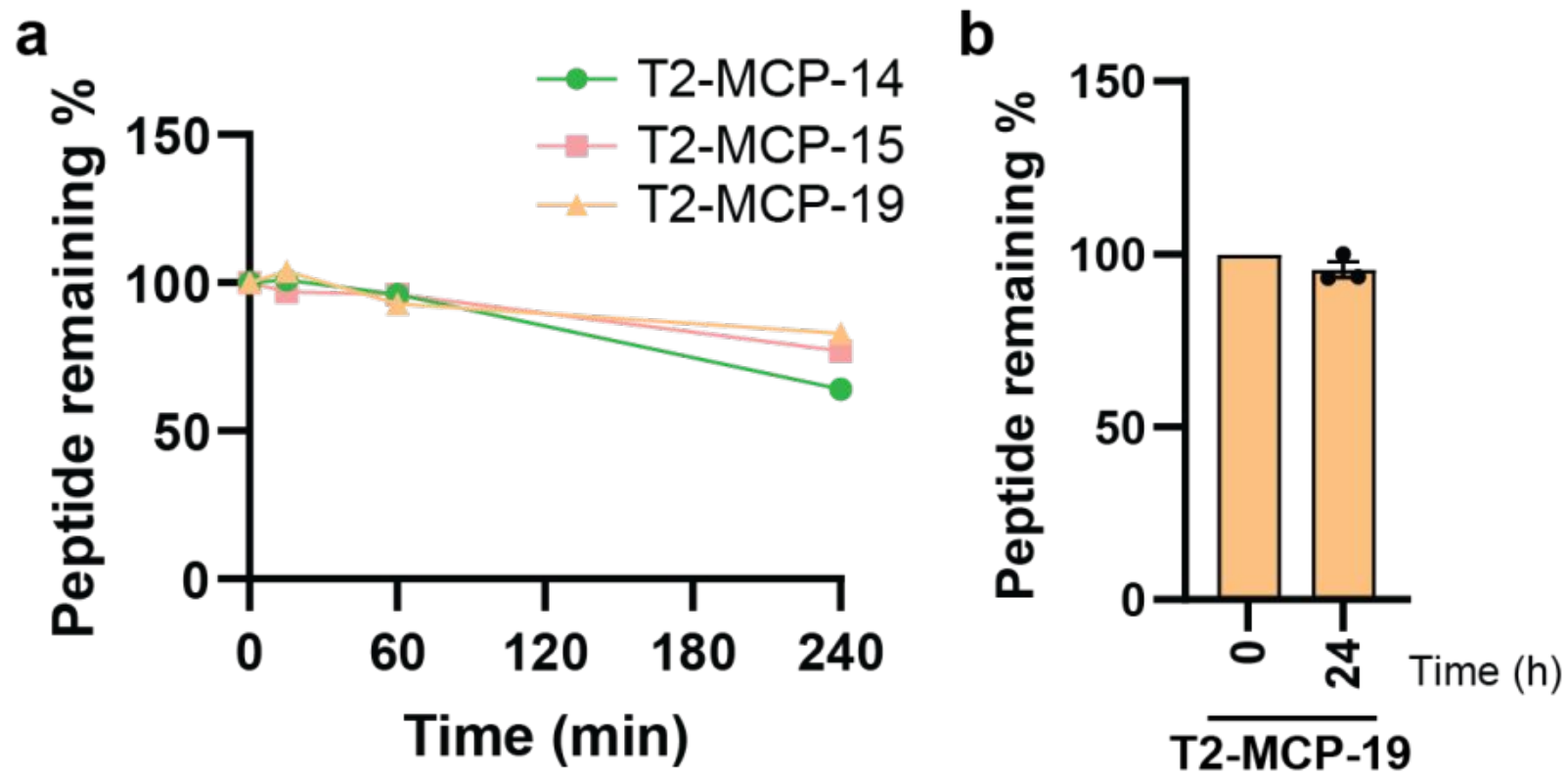

Figure S12. The rationally designed T2-MCP-09 analogues T2-MCP-14, T2-MCP-15 and T2-MCP-19 have improved proteolytic stability. **a)** In vitro mouse plasma stability of T2-MCP-14, T2-MCP-15 and T2-MCP-19. The remaining intact peptide was quantified by LC-MS. Data points are shown as the average of two independent experiments. **b)** Stability of T2-MCP-19 in the presence of recombinant TMPRSS2 (1:1 molar ratio) for 24 h. Remaining intact peptide was quantified by LC-MS. Data shown are the average ± SD of three independent experiments.

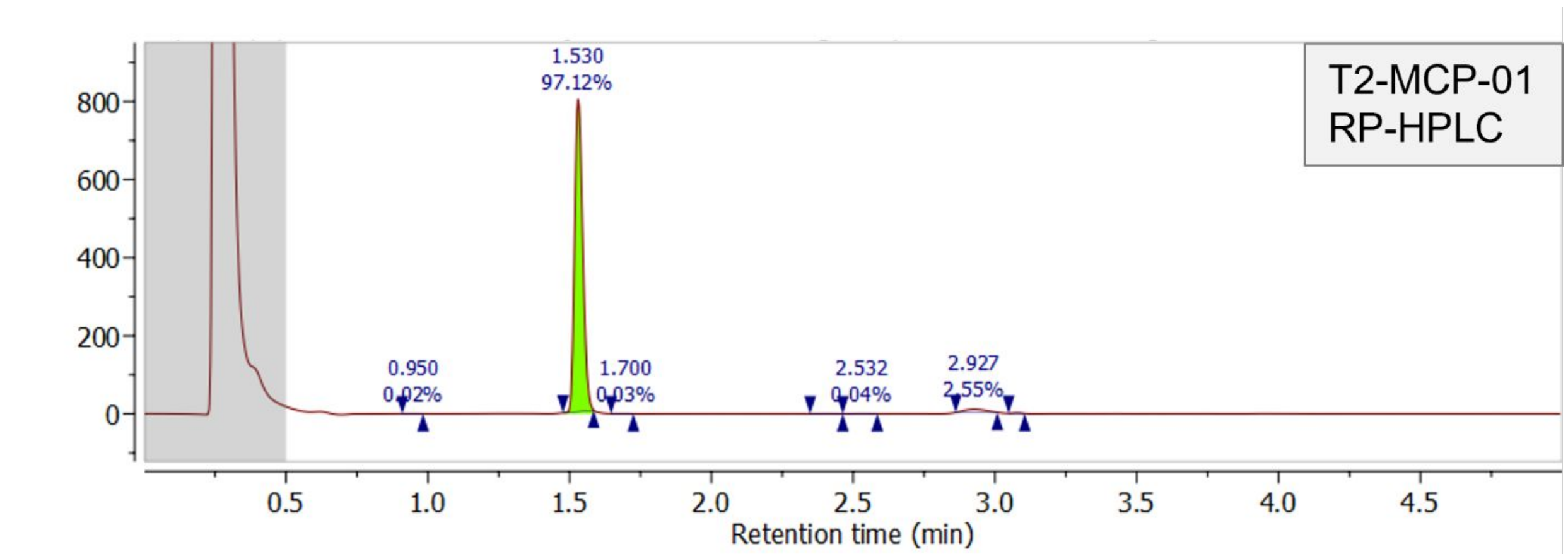
T2-MCP-01
RP-HPLC
1.530
97.12%
0.950
0.02%
1.700
0.03%
2.532
0.04%
2.927
2.55%
Retention time (min)

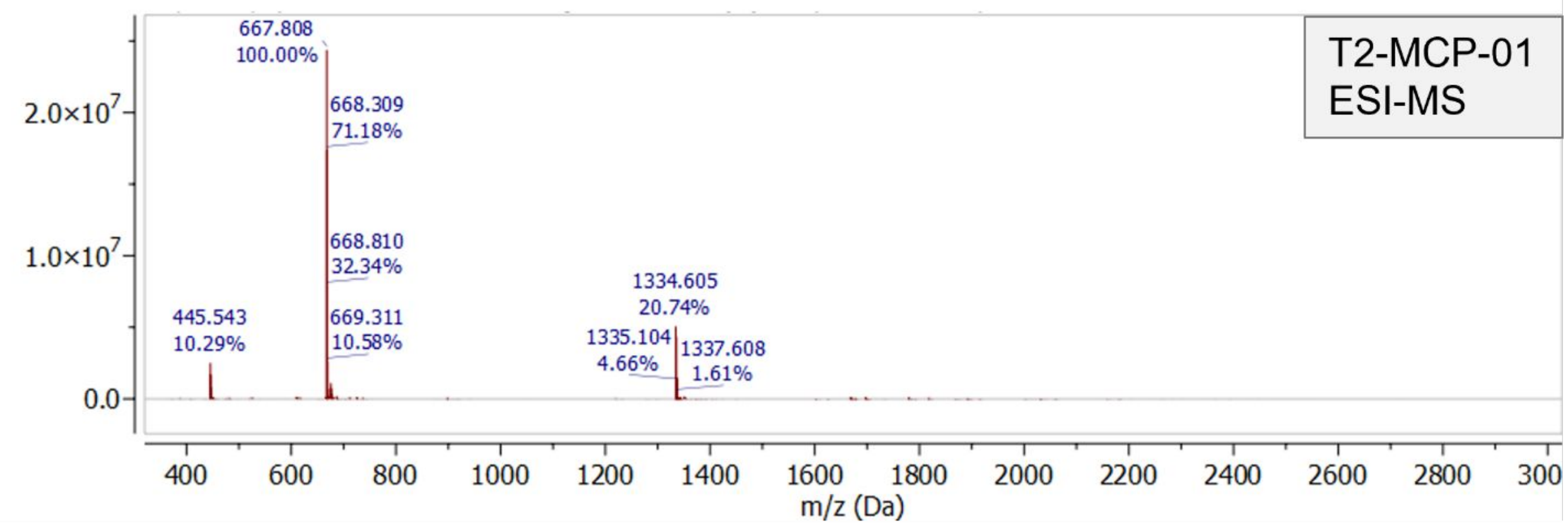
T2-MCP-01
ESI-MS
667.808
100.00%
668.309
71.18%
668.810
32.34%
669.311
10.58%
445.543
10.29%
1334.605
20.74%
1335.104
4.66%
1337.608
1.61%
m/z (Da)

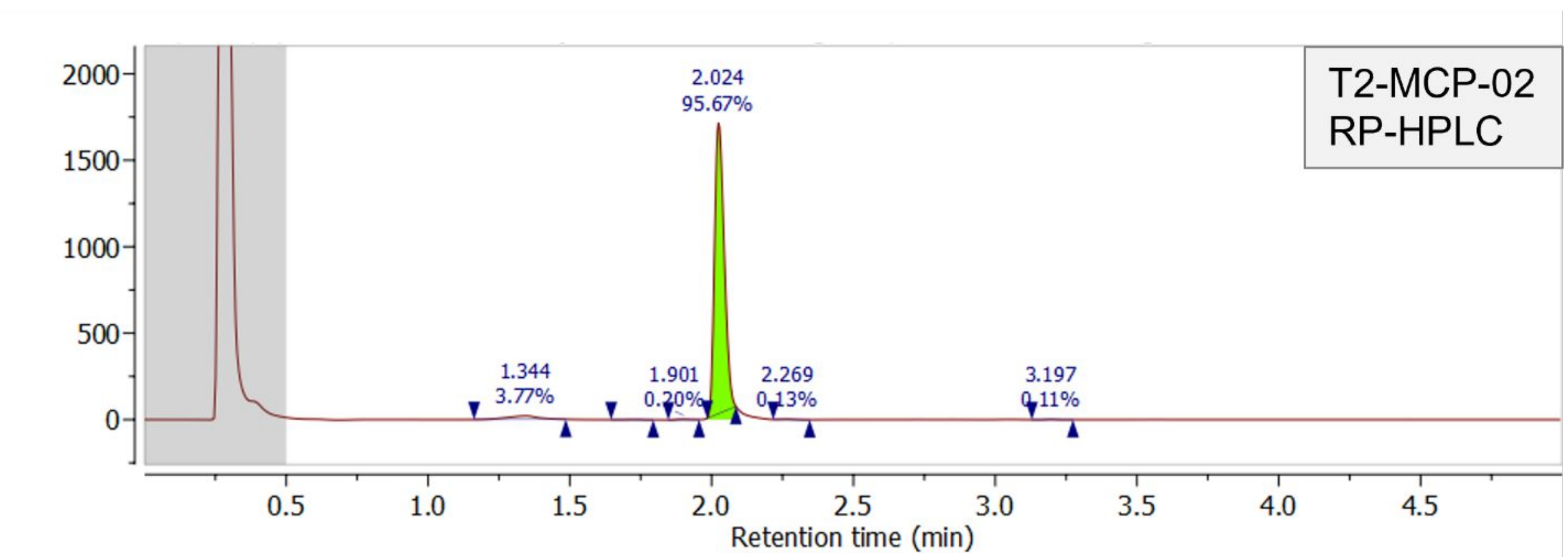
T2-MCP-02
RP-HPLC
2.024
95.67%
1.344
3.77%
1.901
0.20%
2.269
0.13%
3.197
0.11%
Retention time (min)

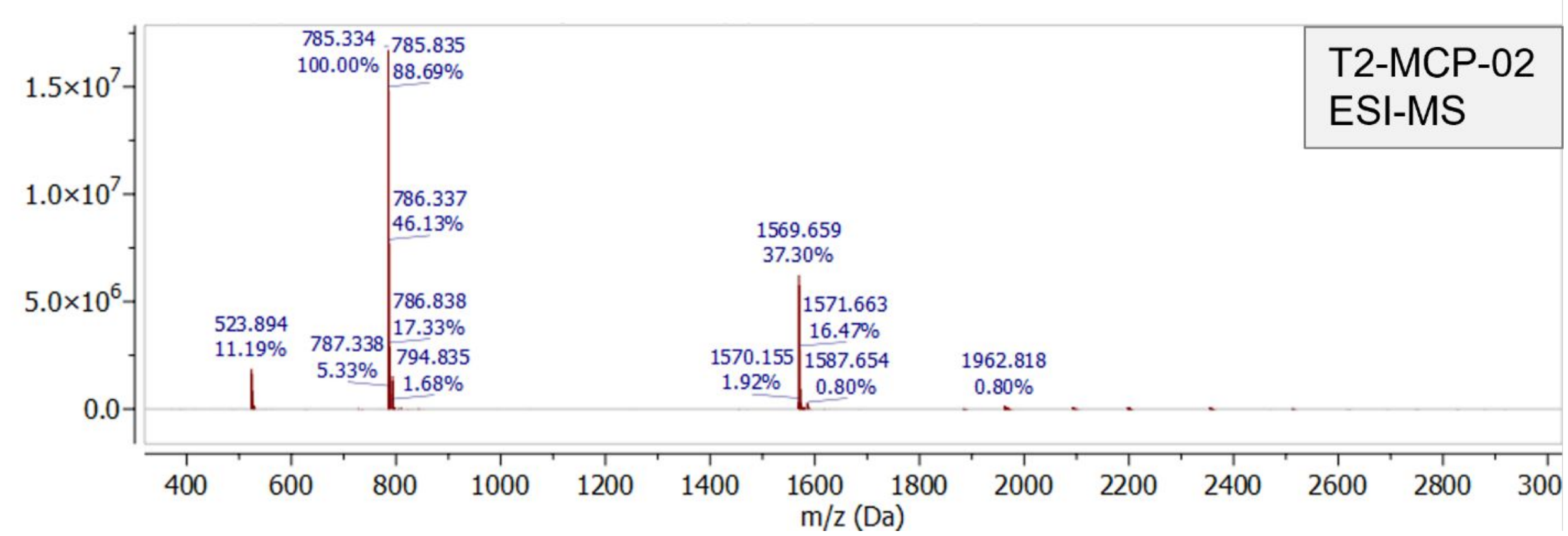
T2-MCP-02
ESI-MS
785.334
100.00%
785.835
88.69%
786.337
46.13%
786.838
17.33%
794.835
1.68%
787.338
5.33%
523.894
11.19%
1569.659
37.30%
1571.663
16.47%
1570.155
1.92%
1587.654
0.80%
1962.818
0.80%
m/z (Da)

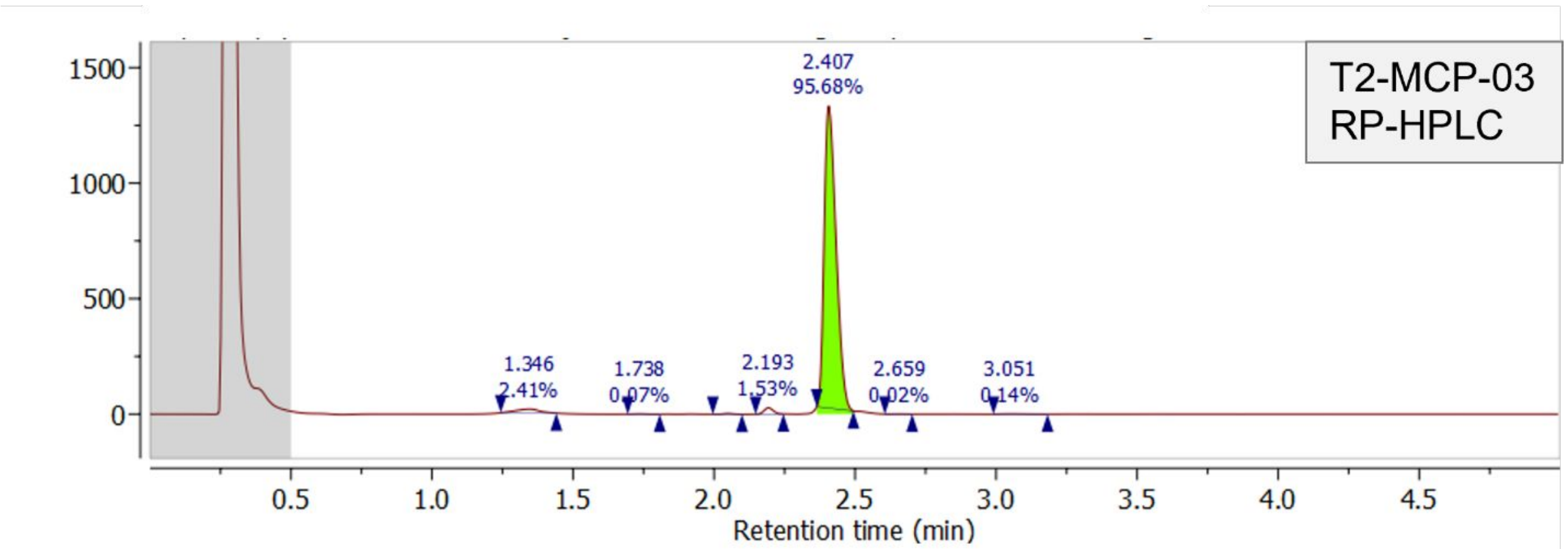
T2-MCP-03
RP-HPLC
2.407
95.68%
1.346
2.41%
1.738
0.07%
2.193
1.53%
2.659
0.02%
3.051
0.14%
Retention time (min)

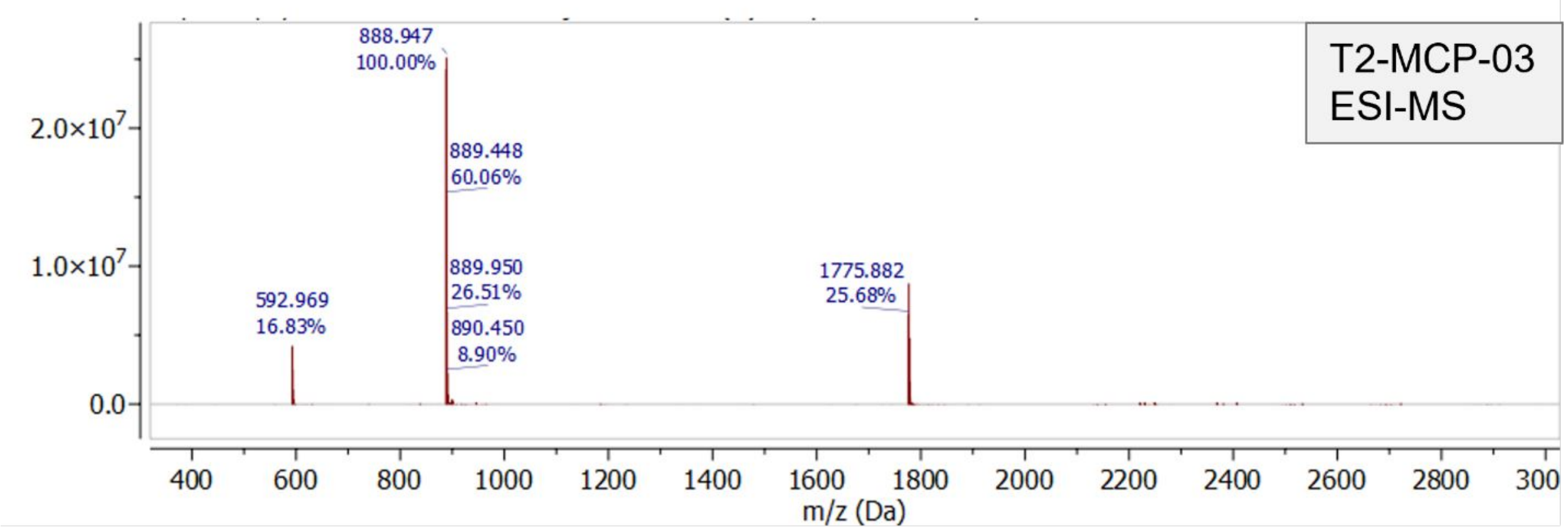
T2-MCP-03
ESI-MS
888.947
100.00%
889.448
60.06%
889.950
26.51%
890.450
8.90%
592.969
16.83%
1775.882
25.68%
m/z (Da)

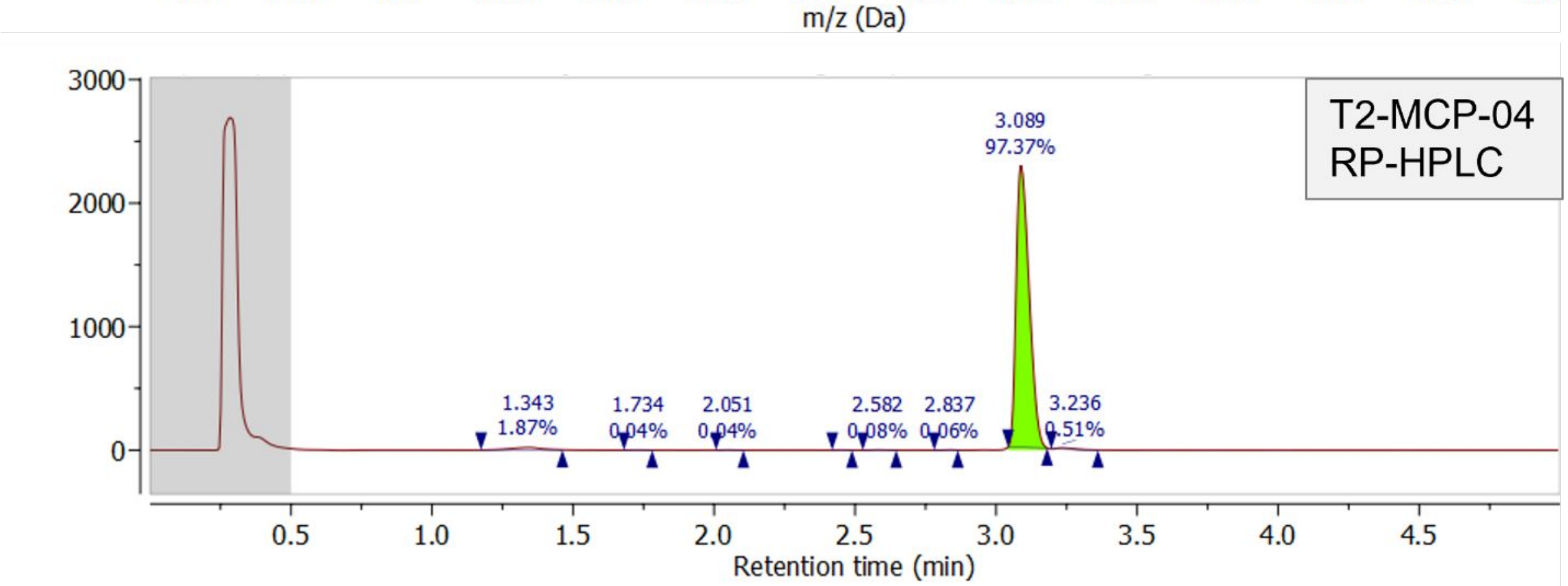
T2-MCP-04
RP-HPLC
3.089
97.37%
1.343
1.87%
1.734
0.04%
2.051
0.04%
2.582
0.08%
2.837
0.06%
3.236
0.51%
Retention time (min)

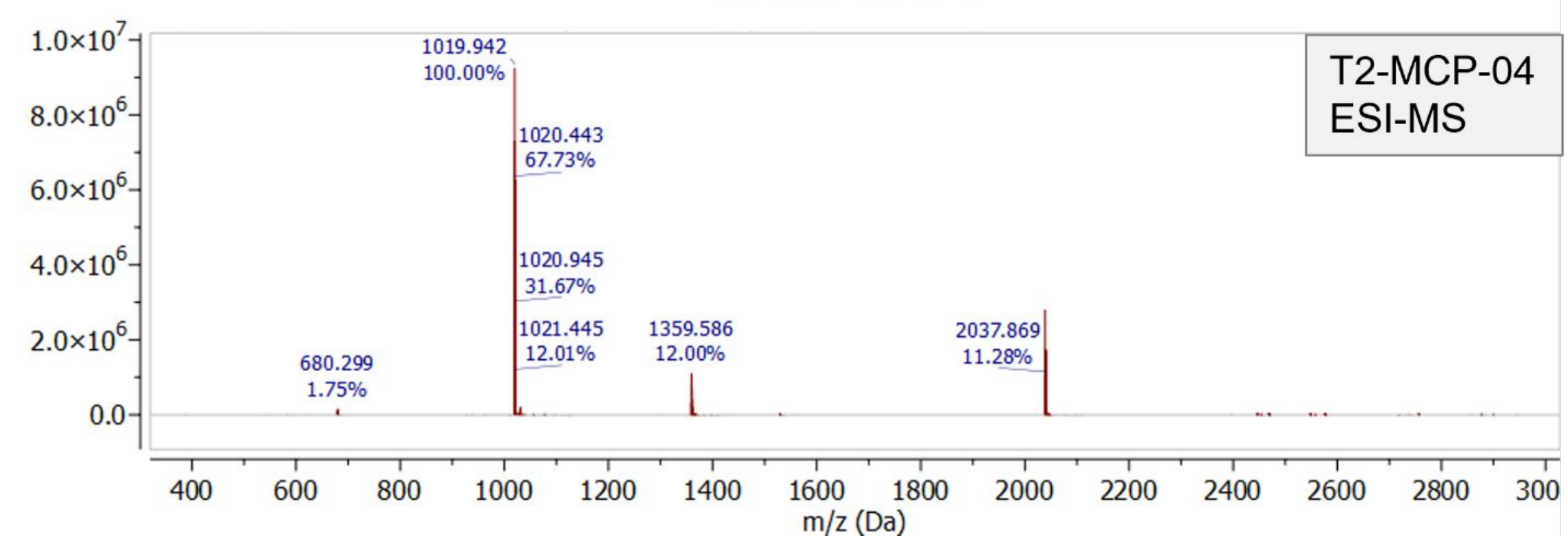
T2-MCP-04
ESI-MS
1019.942
100.00%
1020.443
67.73%
1020.945
31.67%
1021.445
12.01%
1359.586
12.00%
680.299
1.75%
2037.869
11.28%
m/z (Da)

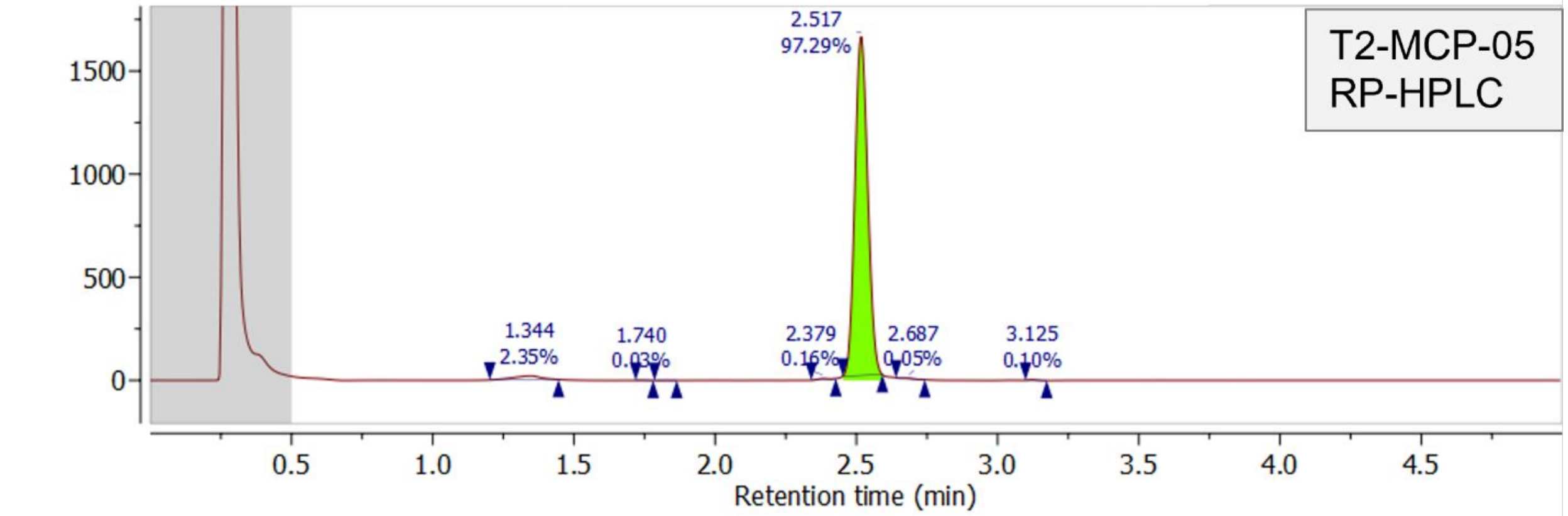
T2-MCP-05
RP-HPLC
2.517
97.29%
1.344
2.35%
1.740
0.03%
2.379
0.16%
2.687
0.05%
3.125
0.10%
1500
1000
500
0
0.5
1.0
1.5
2.0
2.5
3.0
3.5
4.0
4.5
Retention time (min)

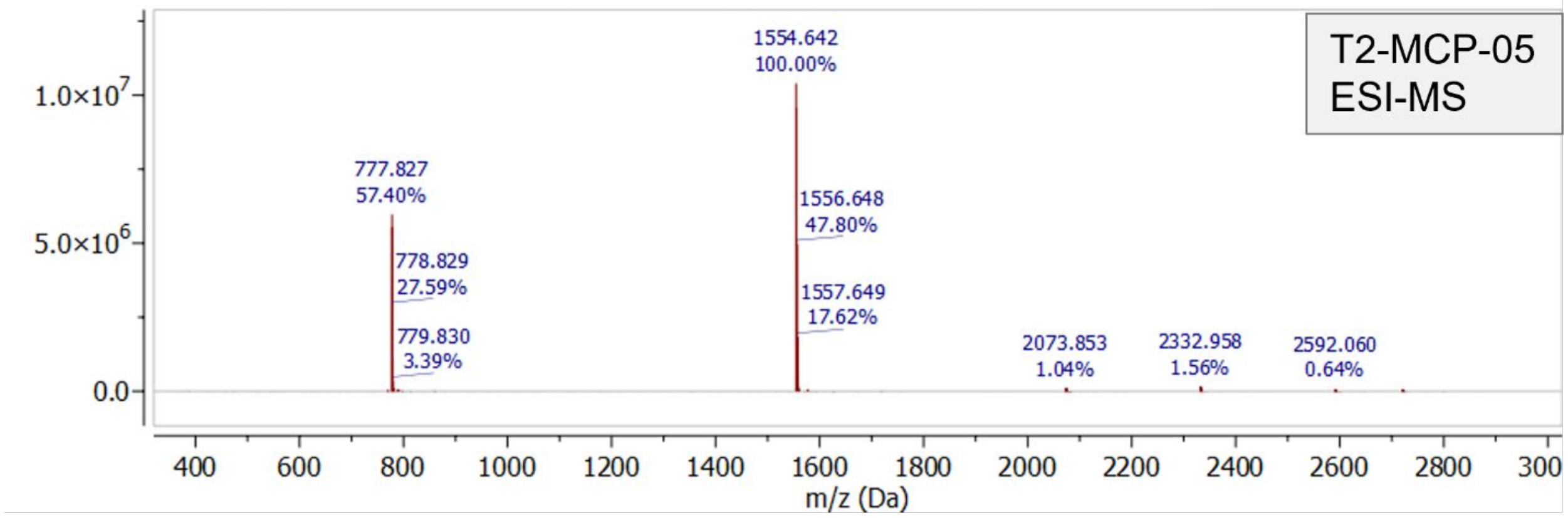
T2-MCP-05
ESI-MS
1554.642
100.00%
777.827
57.40%
1556.648
47.80%
778.829
27.59%
1557.649
17.62%
779.830
3.39%
2073.853
1.04%
2332.958
1.56%
2592.060
0.64%
1.0×10^7
5.0×10^6
0.0
400
600
800
1000
1200
1400
1600
1800
2000
2200
2400
2600
2800
300
m/z (Da)

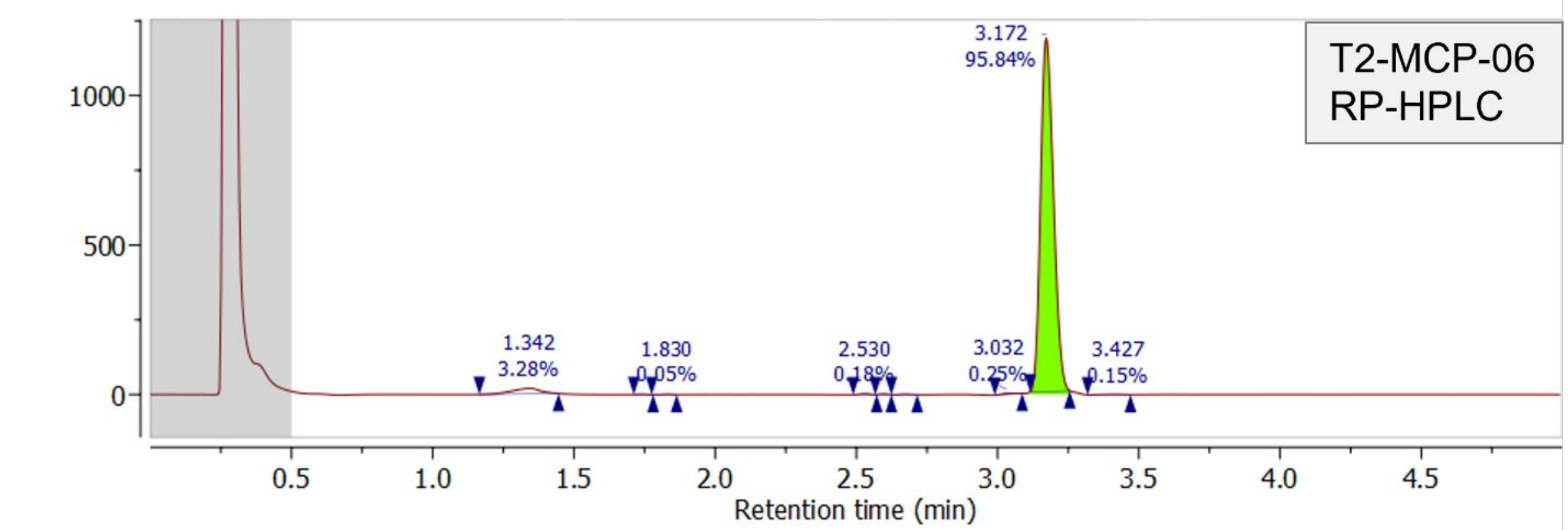
T2-MCP-06
RP-HPLC
3.172
95.84%
1.342
3.28%
1.830
0.05%
2.530
0.18%
3.032
0.25%
3.427
0.15%
1000
500
0
0.5
1.0
1.5
2.0
2.5
3.0
3.5
4.0
4.5
Retention time (min)

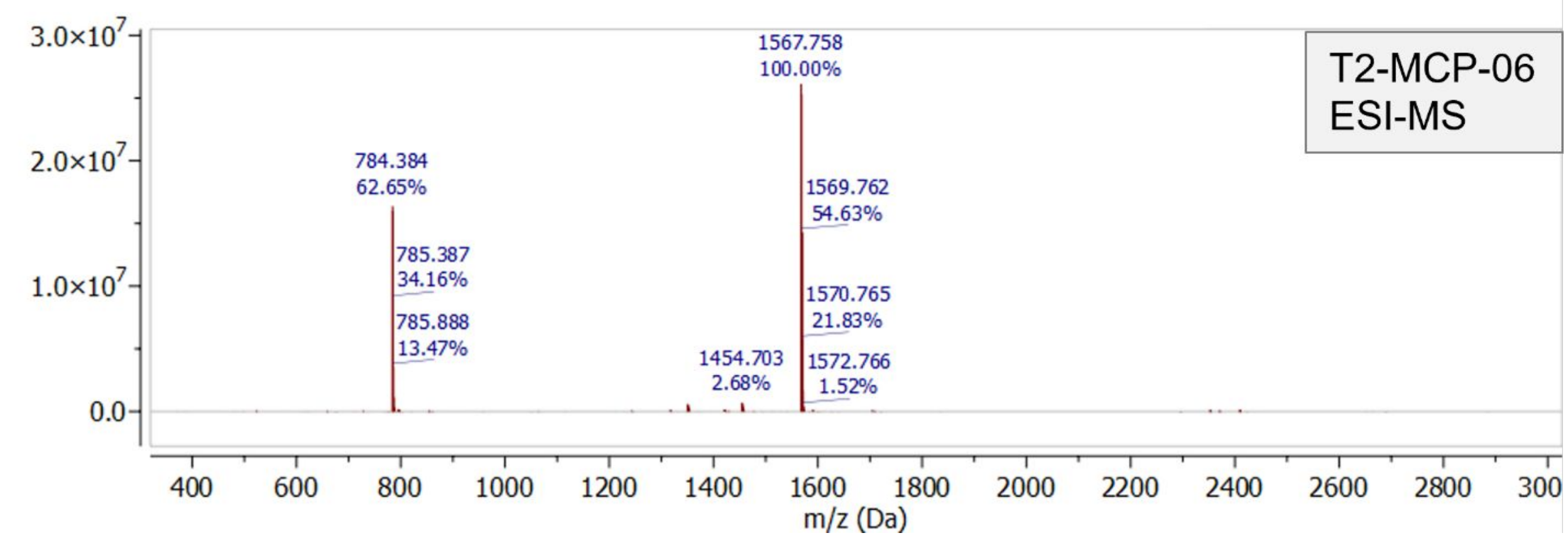
T2-MCP-06
ESI-MS
1567.758
100.00%
784.384
62.65%
1569.762
54.63%
785.387
34.16%
1570.765
21.83%
785.888
13.47%
1454.703
2.68%
1572.766
1.52%
3.0×10^7
2.0×10^7
1.0×10^7
0.0
400
600
800
1000
1200
1400
1600
1800
2000
2200
2400
2600
2800
300
m/z (Da)

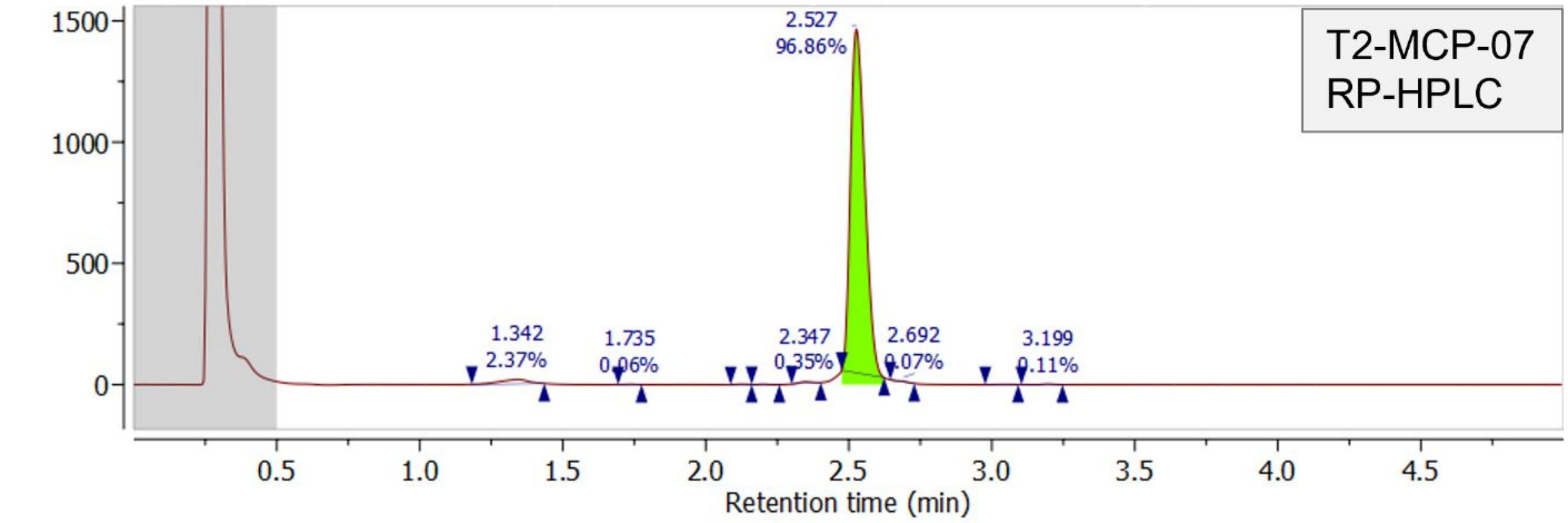
T2-MCP-07
RP-HPLC
2.527
96.86%
1.342
2.37%
1.735
0.06%
2.347
0.35%
2.692
0.07%
3.199
0.11%
Retention time (min)

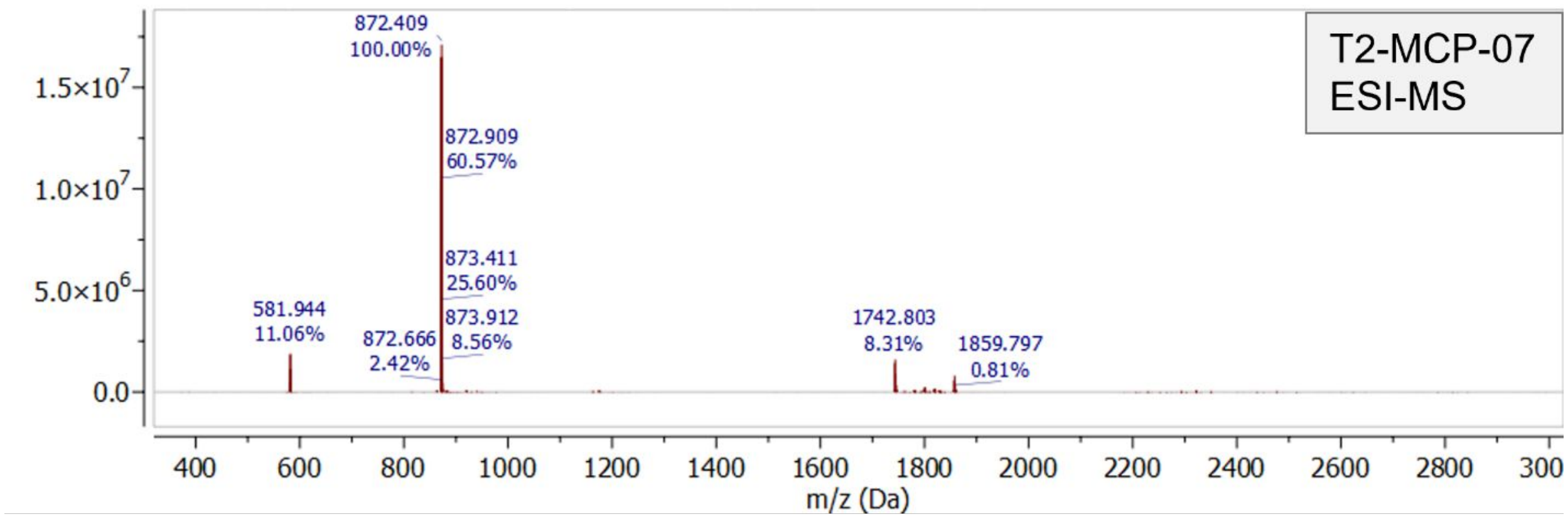
T2-MCP-07
ESI-MS
872.409
100.00%
872.909
60.57%
873.411
25.60%
873.912
8.56%
581.944
11.06%
872.666
2.42%
1742.803
8.31%
1859.797
0.81%
m/z (Da)

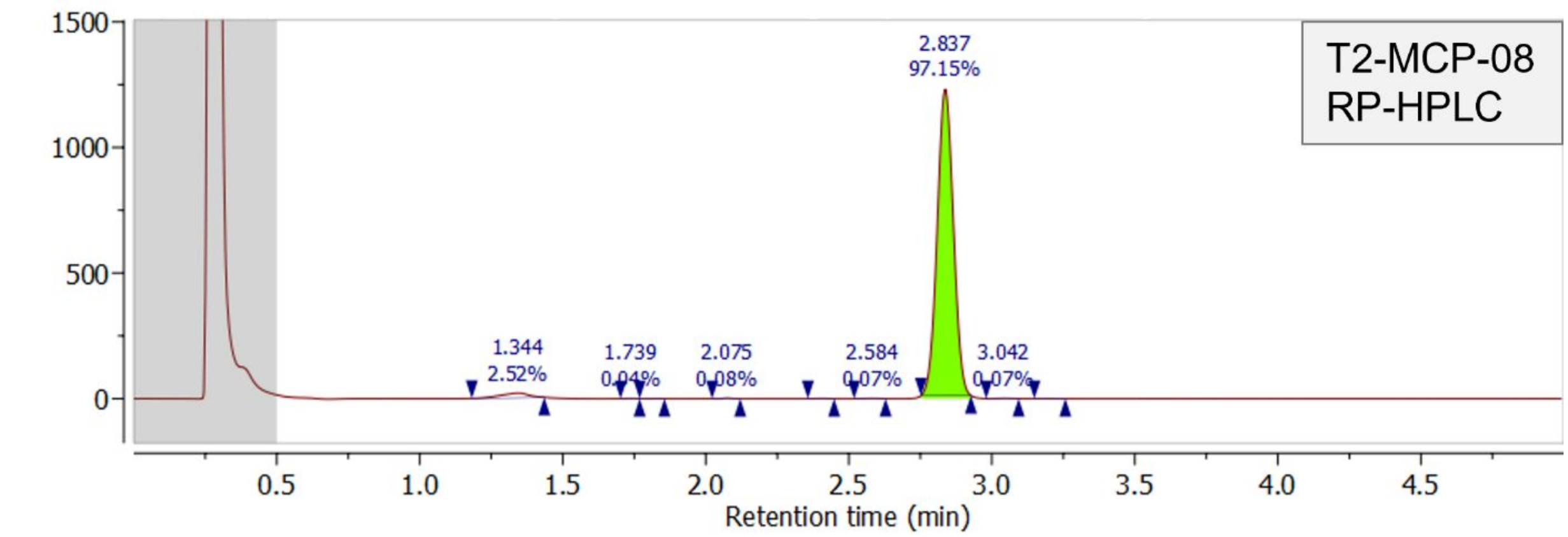
T2-MCP-08
RP-HPLC
2.837
97.15%
1.344
2.52%
1.739
0.04%
2.075
0.08%
2.584
0.07%
3.042
0.07%
Retention time (min)

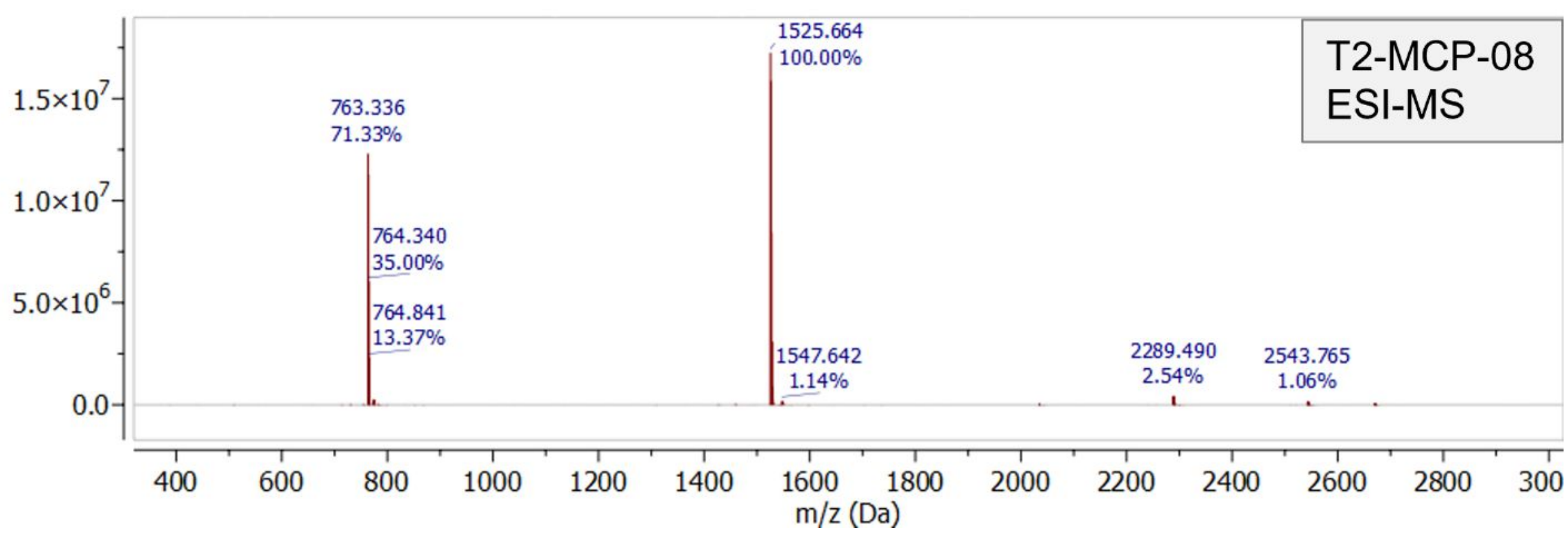
T2-MCP-08
ESI-MS
1525.664
100.00%
763.336
71.33%
764.340
35.00%
764.841
13.37%
1547.642
1.14%
2289.490
2.54%
2543.765
1.06%
m/z (Da)

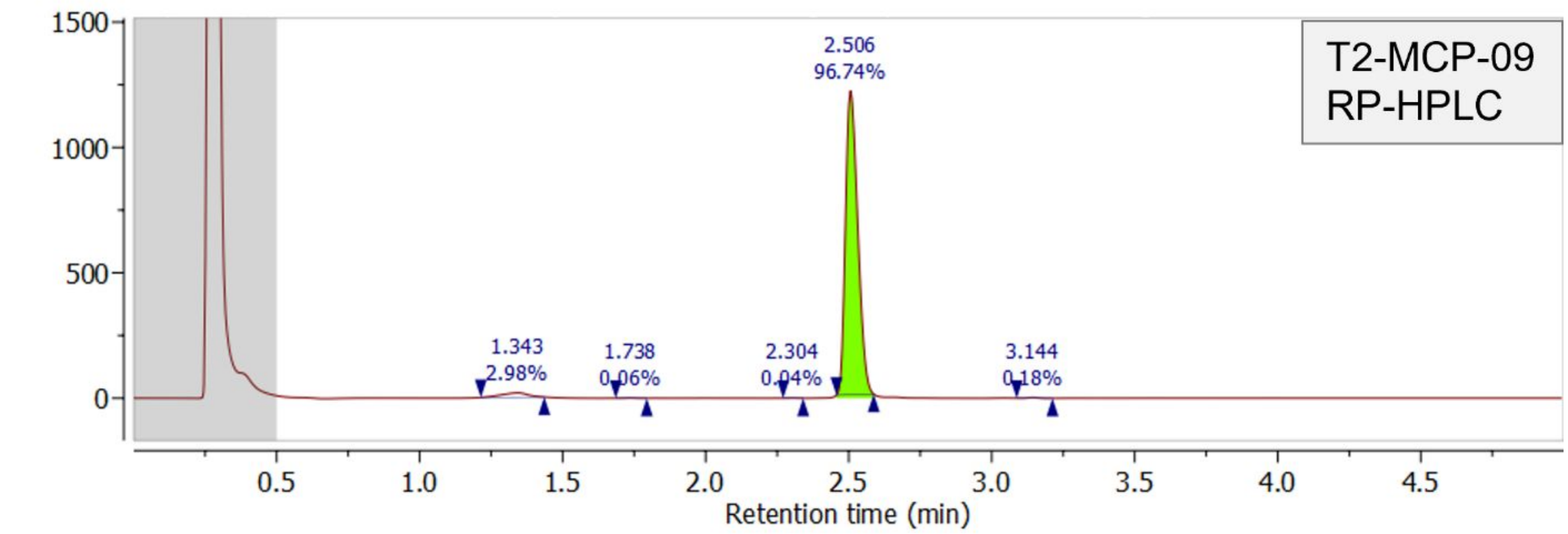
T2-MCP-09
RP-HPLC
2.506
96.74%
1.343
2.98%
1.738
0.06%
2.304
0.04%
3.144
0.18%
Retention time (min)

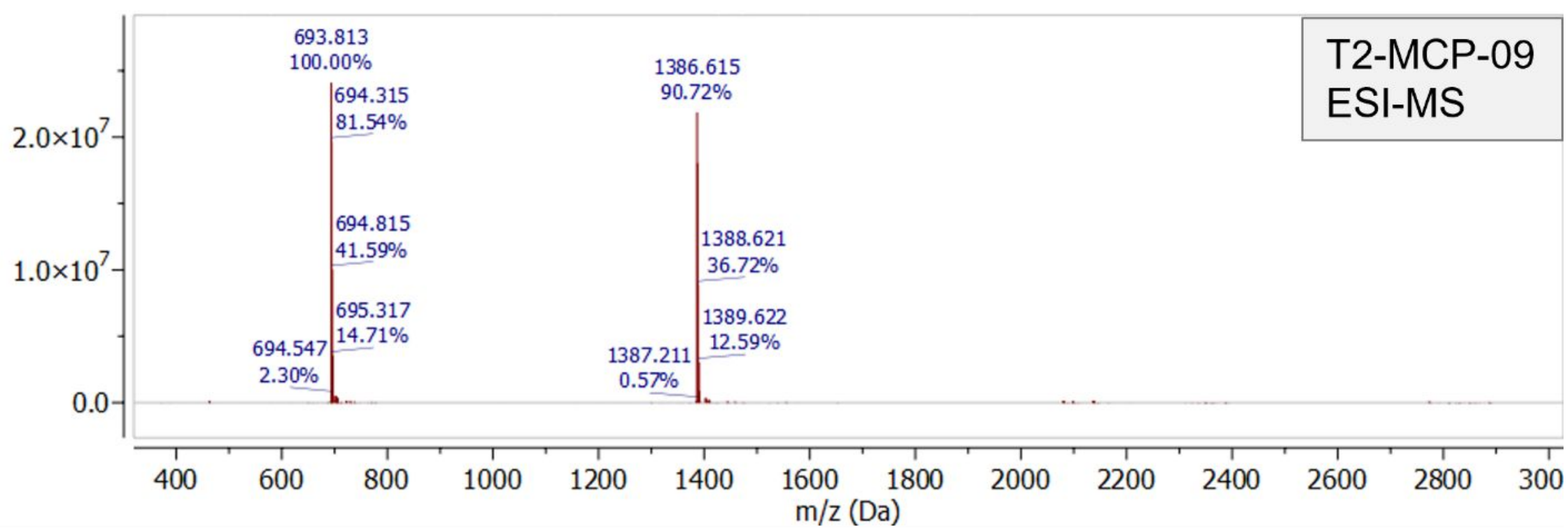
T2-MCP-09
ESI-MS
693.813
100.00%
694.315
81.54%
694.815
41.59%
695.317
14.71%
694.547
2.30%
1386.615
90.72%
1388.621
36.72%
1389.622
12.59%
1387.211
0.57%
m/z (Da)

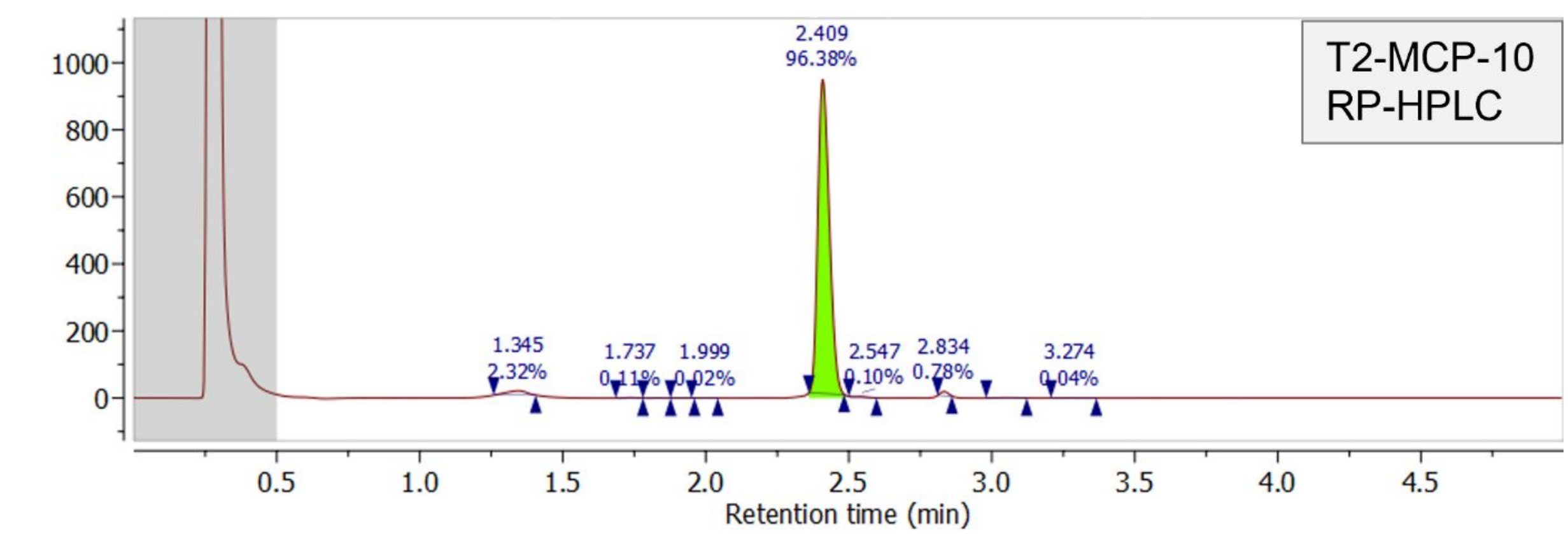
T2-MCP-10
RP-HPLC
2.409
96.38%
1.345
2.32%
1.737
0.11%
1.999
0.02%
2.547
0.10%
2.834
0.78%
3.274
0.04%
Retention time (min)

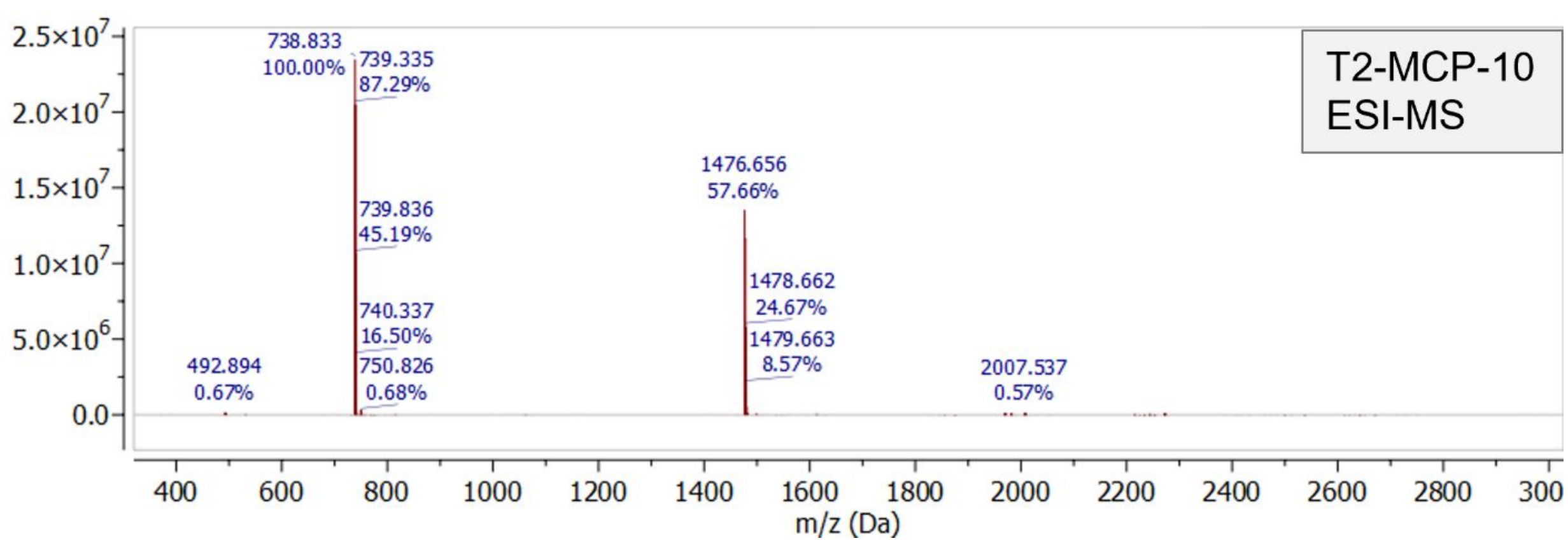
T2-MCP-10
ESI-MS
738.833
100.00%
739.335
87.29%
739.836
45.19%
740.337
16.50%
750.826
0.68%
492.894
0.67%
1476.656
57.66%
1478.662
24.67%
1479.663
8.57%
2007.537
0.57%
m/z (Da)

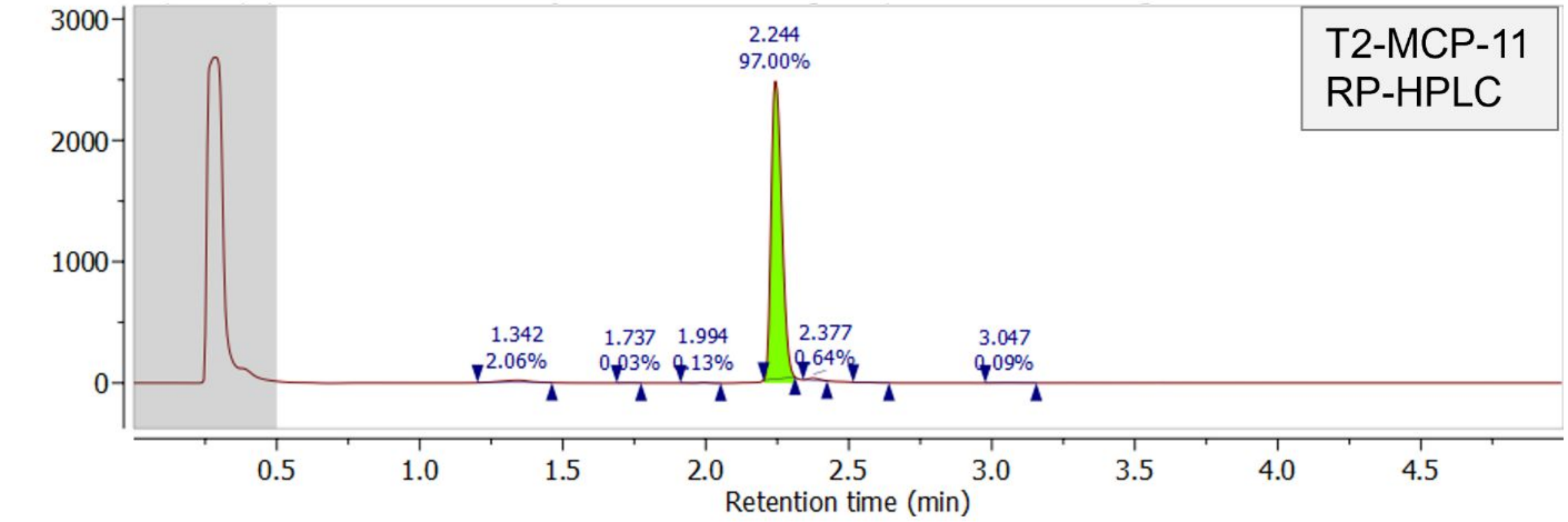
T2-MCP-11
RP-HPLC
2.244
97.00%
1.342
2.06%
1.737
0.03%
1.994
0.13%
2.377
0.64%
3.047
0.09%
3000
2000
1000
0
0.5
1.0
1.5
2.0
2.5
3.0
3.5
4.0
4.5
Retention time (min)

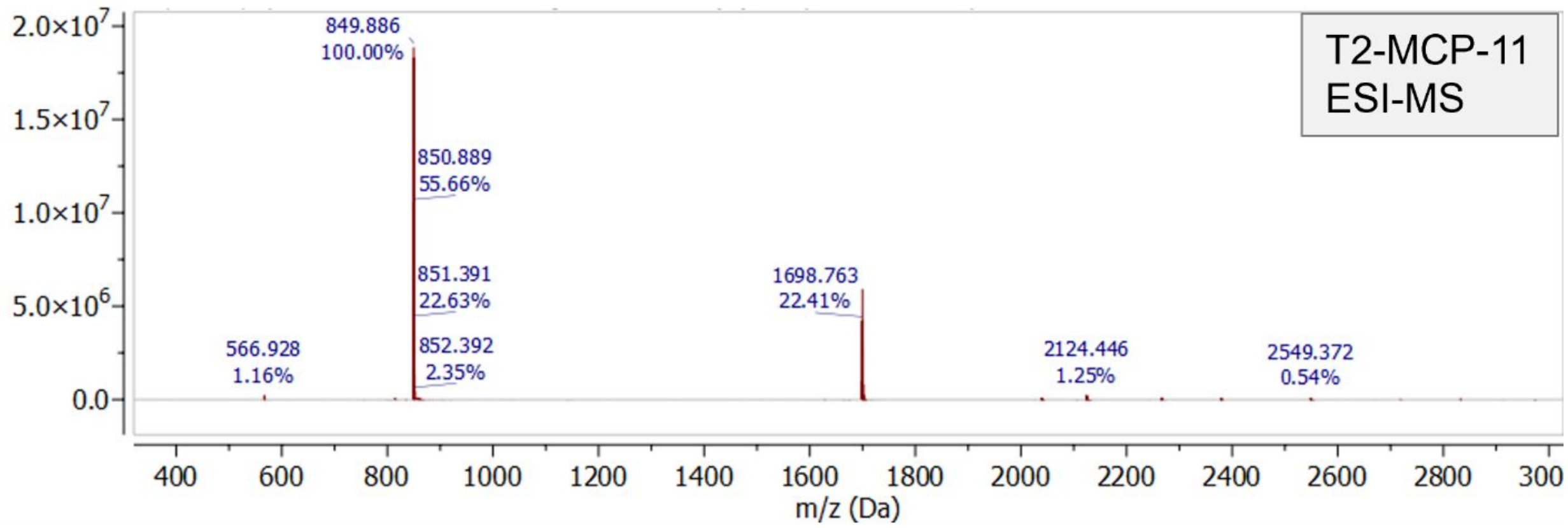
T2-MCP-11
ESI-MS
849.886
100.00%
850.889
55.66%
851.391
22.63%
852.392
2.35%
566.928
1.16%
1698.763
22.41%
2124.446
1.25%
2549.372
0.54%
2.0×10^7
1.5×10^7
1.0×10^7
5.0×10^6
0.0
400
600
800
1000
1200
1400
1600
1800
2000
2200
2400
2600
2800
300
m/z (Da)

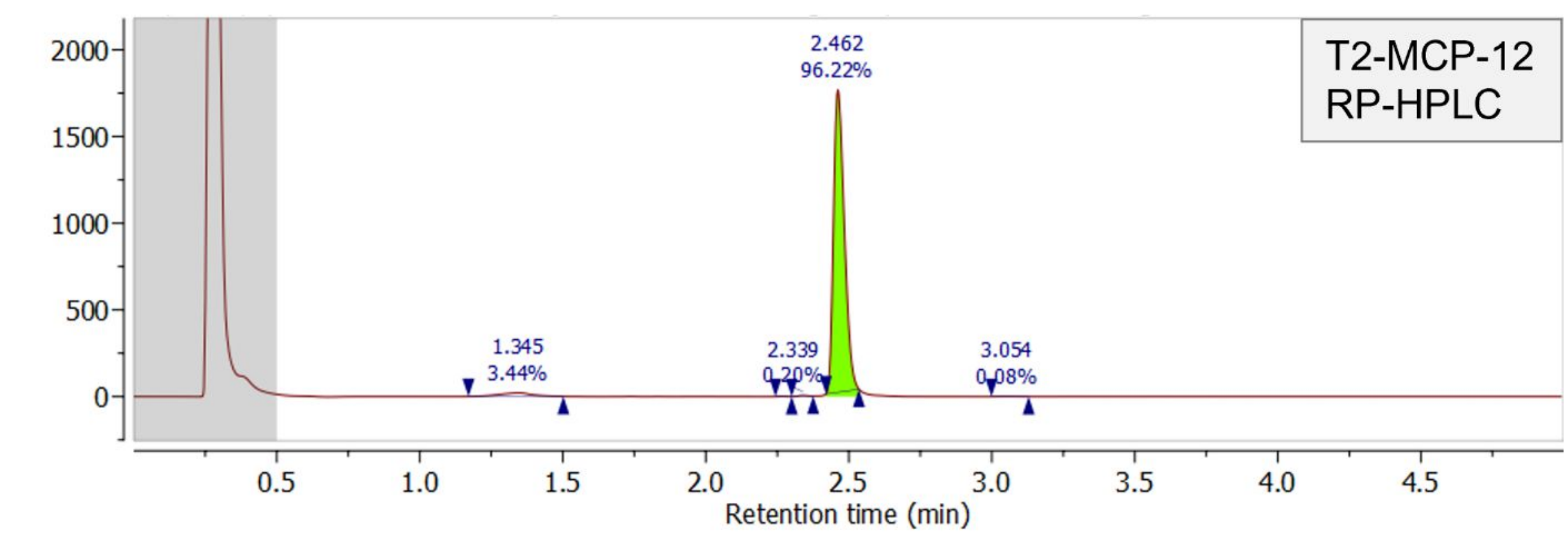
T2-MCP-12
RP-HPLC
2.462
96.22%
1.345
3.44%
2.339
0.20%
3.054
0.08%
2000
1500
1000
500
0
0.5
1.0
1.5
2.0
2.5
3.0
3.5
4.0
4.5
Retention time (min)

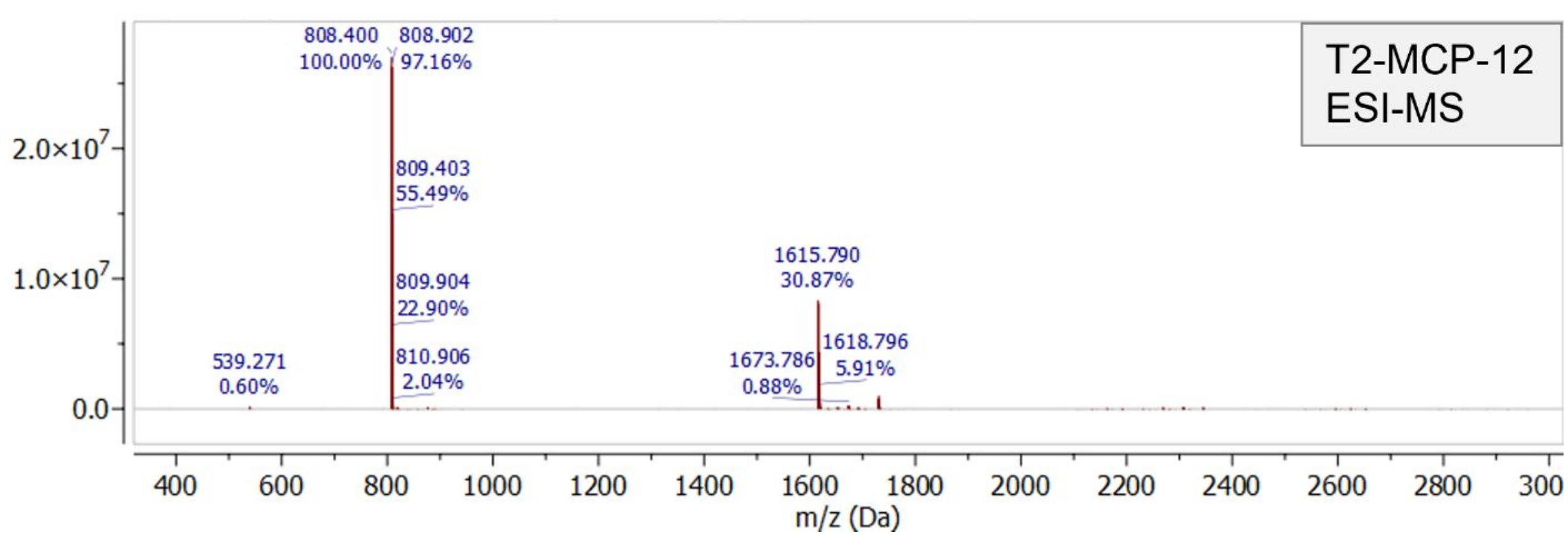
T2-MCP-12
ESI-MS
808.400
100.00%
808.902
97.16%
809.403
55.49%
809.904
22.90%
810.906
2.04%
539.271
0.60%
1615.790
30.87%
1618.796
5.91%
1673.786
0.88%
2.0×10^7
1.0×10^7
0.0
400
600
800
1000
1200
1400
1600
1800
2000
2200
2400
2600
2800
300
m/z (Da)

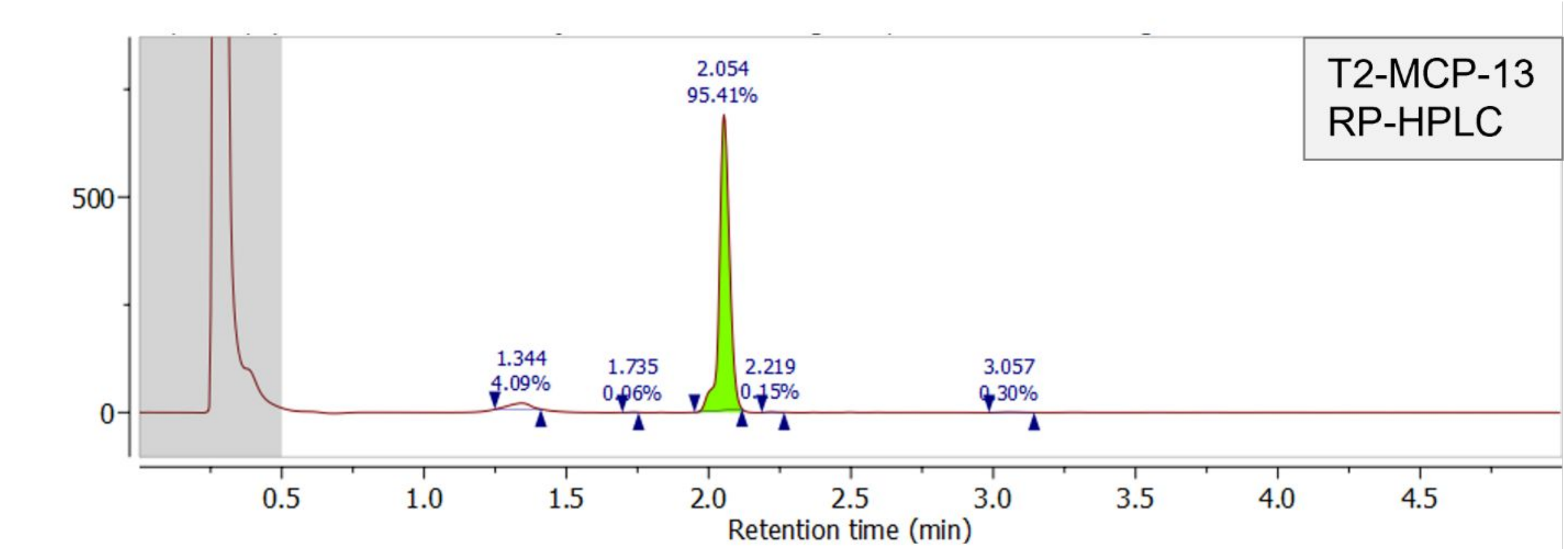
T2-MCP-13
RP-HPLC
2.054
95.41%
1.344
4.09%
1.735
0.06%
2.219
0.15%
3.057
0.30%
Retention time (min)

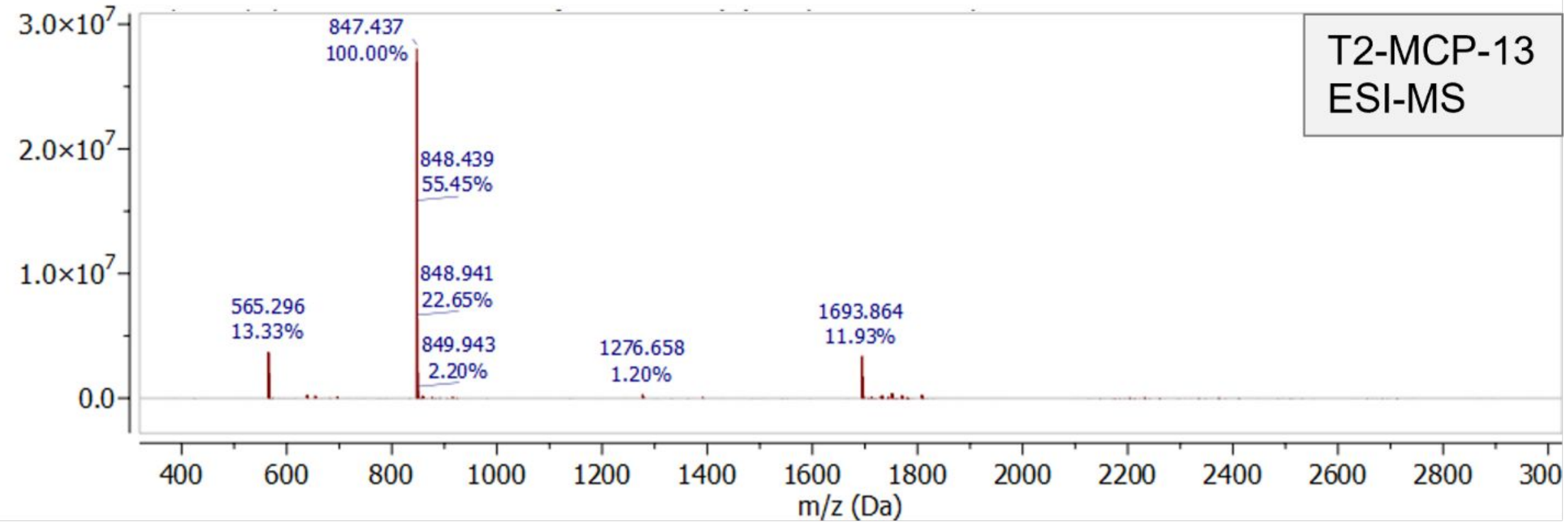
T2-MCP-13
ESI-MS
847.437
100.00%
848.439
55.45%
848.941
22.65%
849.943
2.20%
565.296
13.33%
1276.658
1.20%
1693.864
11.93%
m/z (Da)

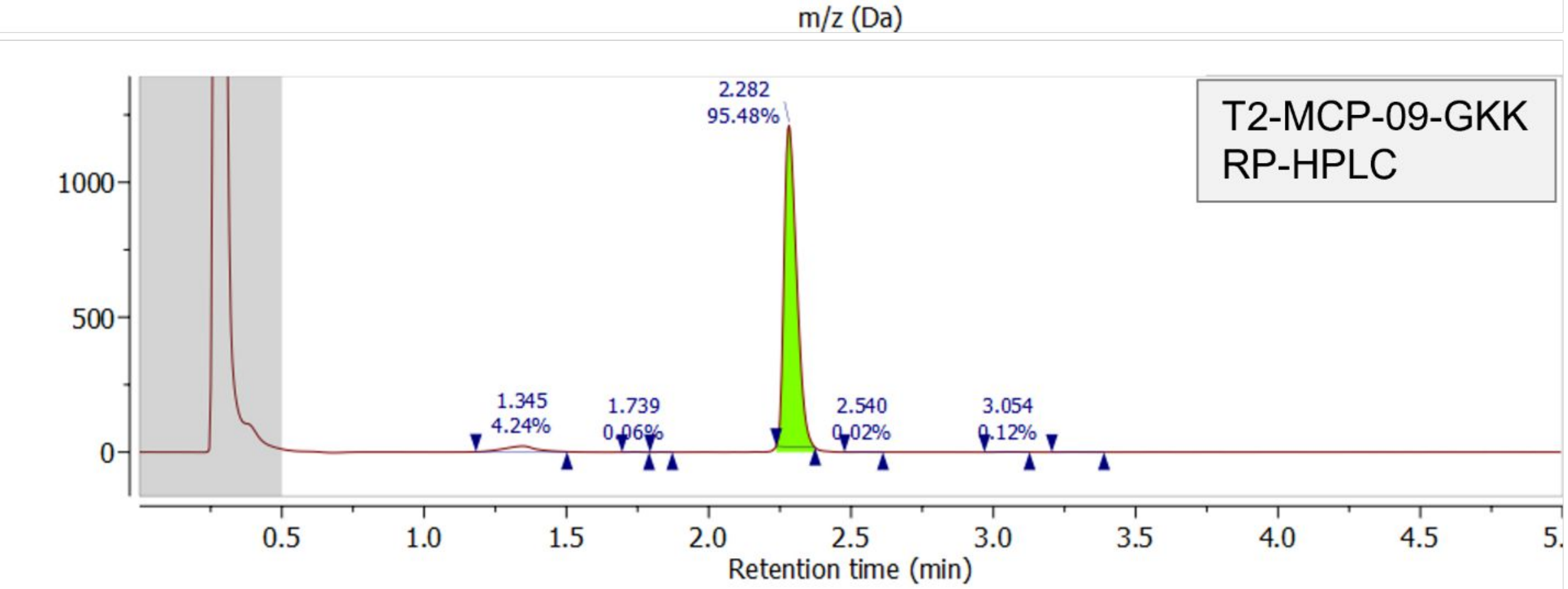
T2-MCP-09-GKK
RP-HPLC
2.282
95.48%
1.345
4.24%
1.739
0.06%
2.540
0.02%
3.054
0.12%
Retention time (min)

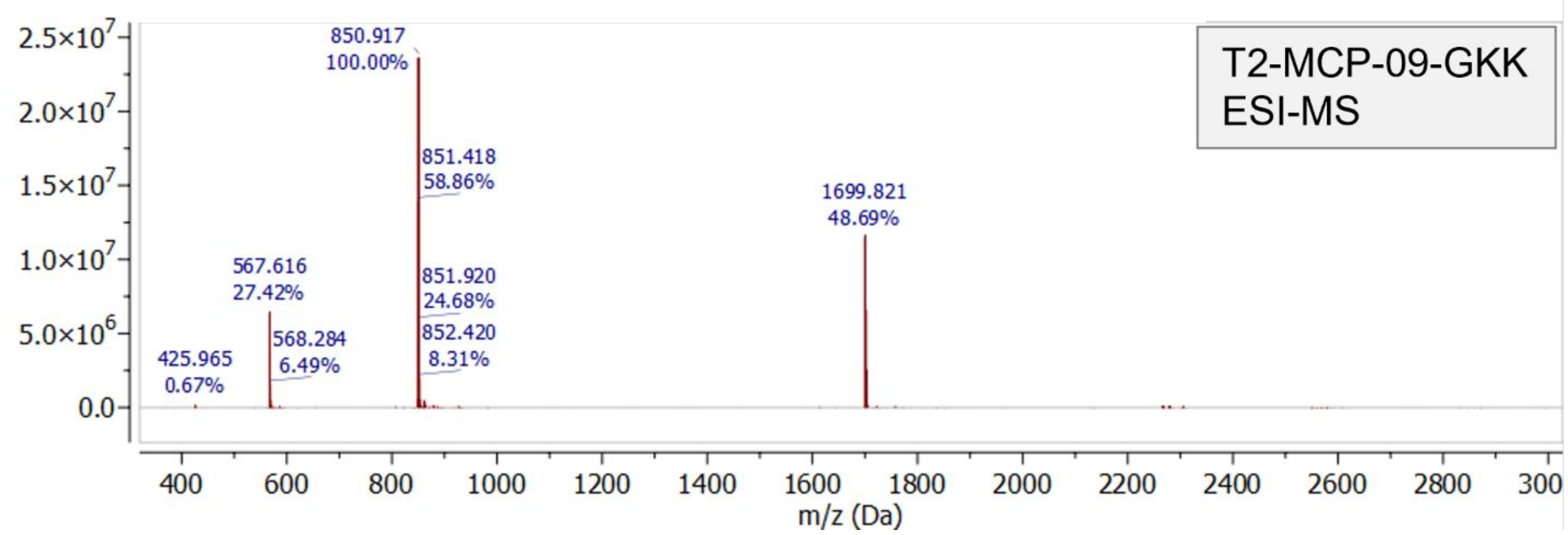
T2-MCP-09-GKK
ESI-MS
850.917
100.00%
851.418
58.86%
851.920
24.68%
852.420
8.31%
567.616
27.42%
568.284
6.49%
425.965
0.67%
1699.821
48.69%
m/z (Da)

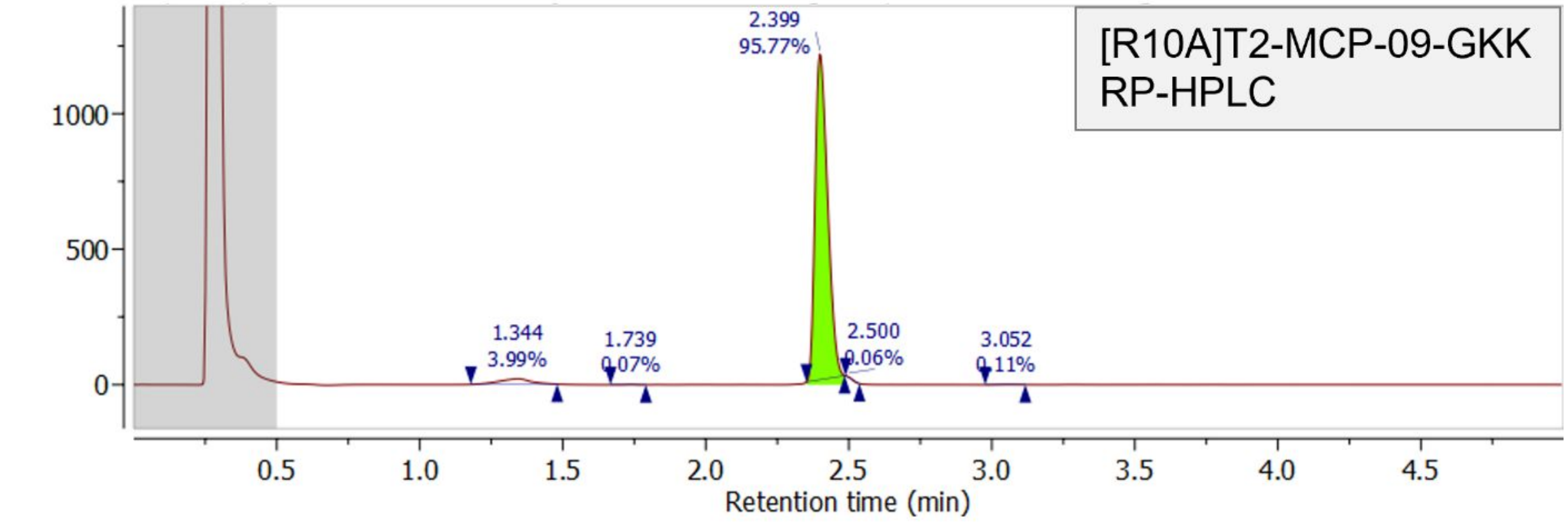
[R10A]T2-MCP-09-GKK
RP-HPLC
2.399
95.77%
1.344
3.99%
1.739
0.07%
2.500
0.06%
3.052
0.11%
Retention time (min)

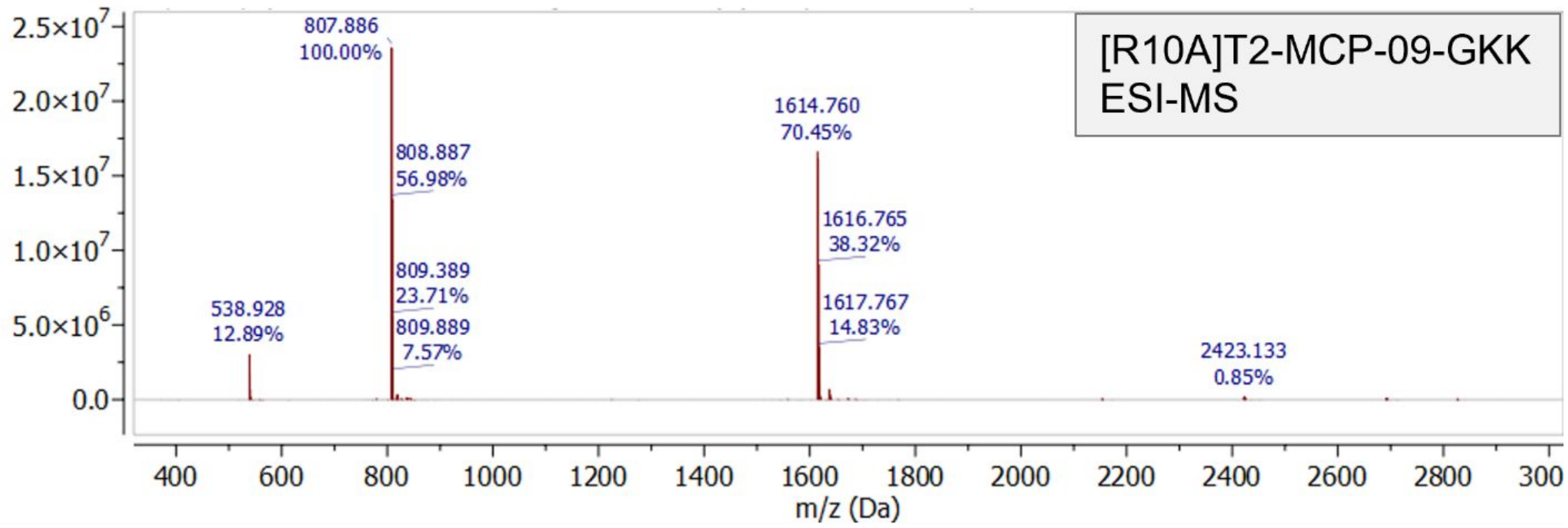
[R10A]T2-MCP-09-GKK
ESI-MS
807.886
100.00%
808.887
56.98%
809.389
23.71%
809.889
7.57%
538.928
12.89%
1614.760
70.45%
1616.765
38.32%
1617.767
14.83%
2423.133
0.85%
m/z (Da)

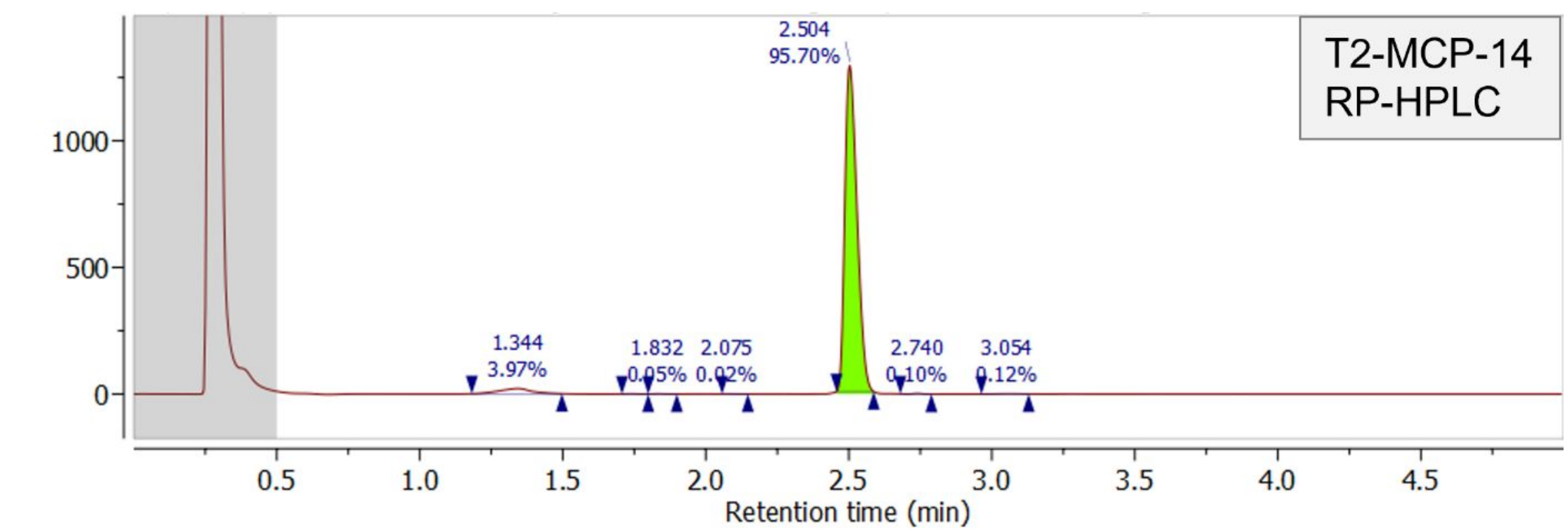
T2-MCP-14
RP-HPLC
2.504
95.70%
1.344
3.97%
1.832
0.05%
2.075
0.02%
2.740
0.10%
3.054
0.12%
Retention time (min)

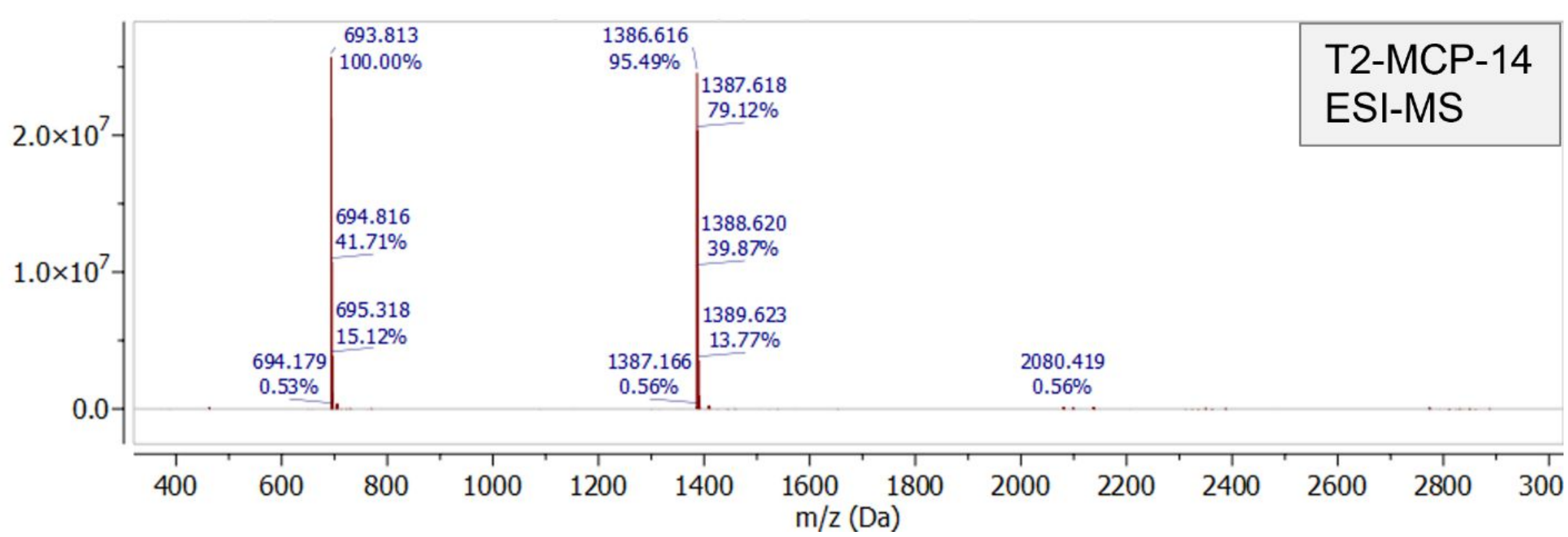
T2-MCP-14
ESI-MS
693.813
100.00%
694.816
41.71%
695.318
15.12%
694.179
0.53%
1386.616
95.49%
1387.618
79.12%
1388.620
39.87%
1389.623
13.77%
1387.166
0.56%
2080.419
0.56%
m/z (Da)

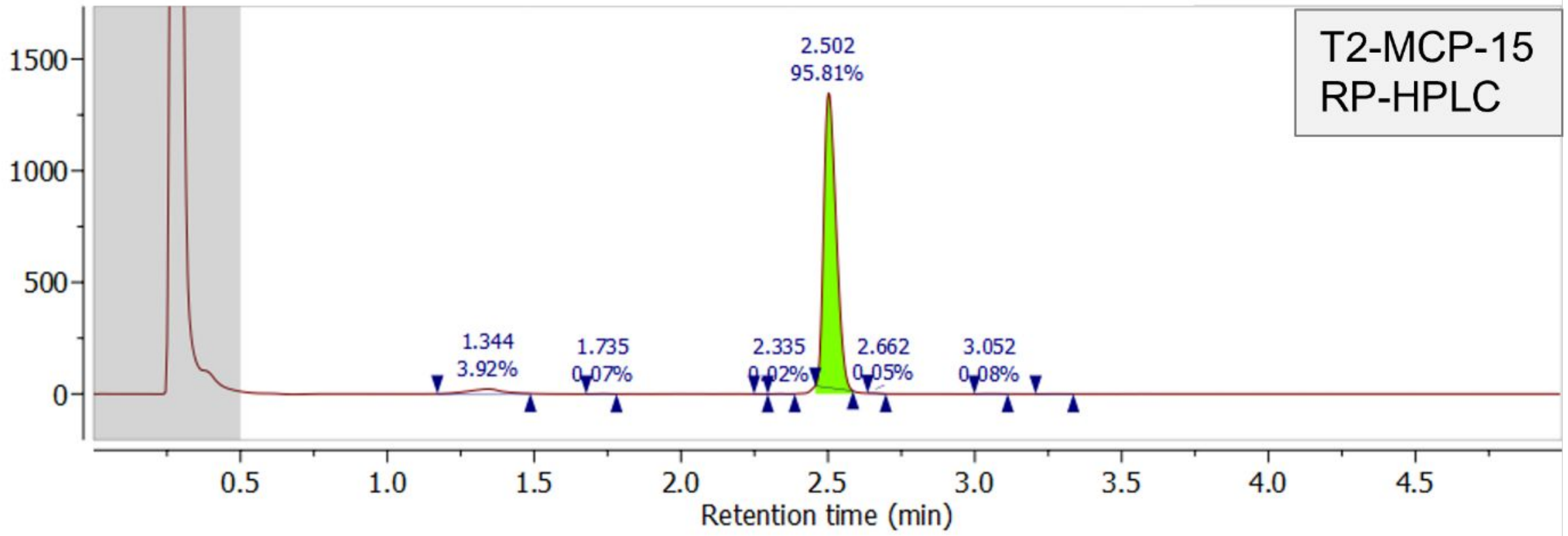
T2-MCP-15
RP-HPLC
2.502
95.81%
1.344
3.92%
1.735
0.07%
2.335
0.02%
2.662
0.05%
3.052
0.08%
Retention time (min)

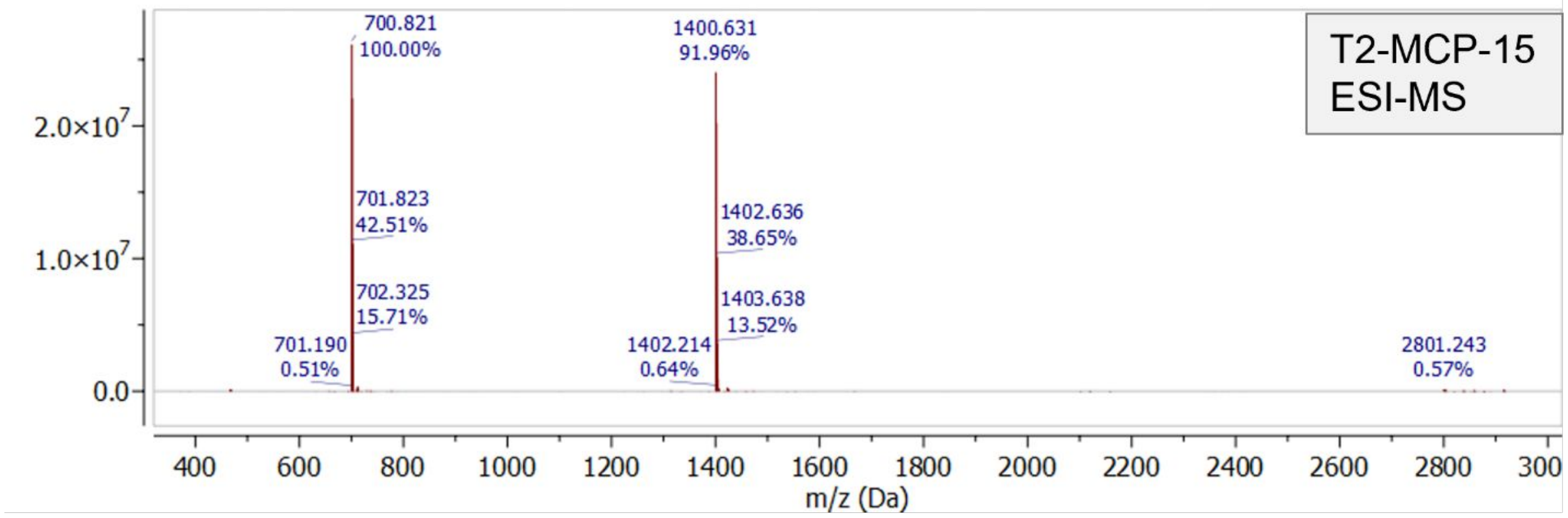
T2-MCP-15
ESI-MS
700.821
100.00%
1400.631
91.96%
701.823
42.51%
1402.636
38.65%
702.325
15.71%
1403.638
13.52%
701.190
0.51%
1402.214
0.64%
2801.243
0.57%
m/z (Da)

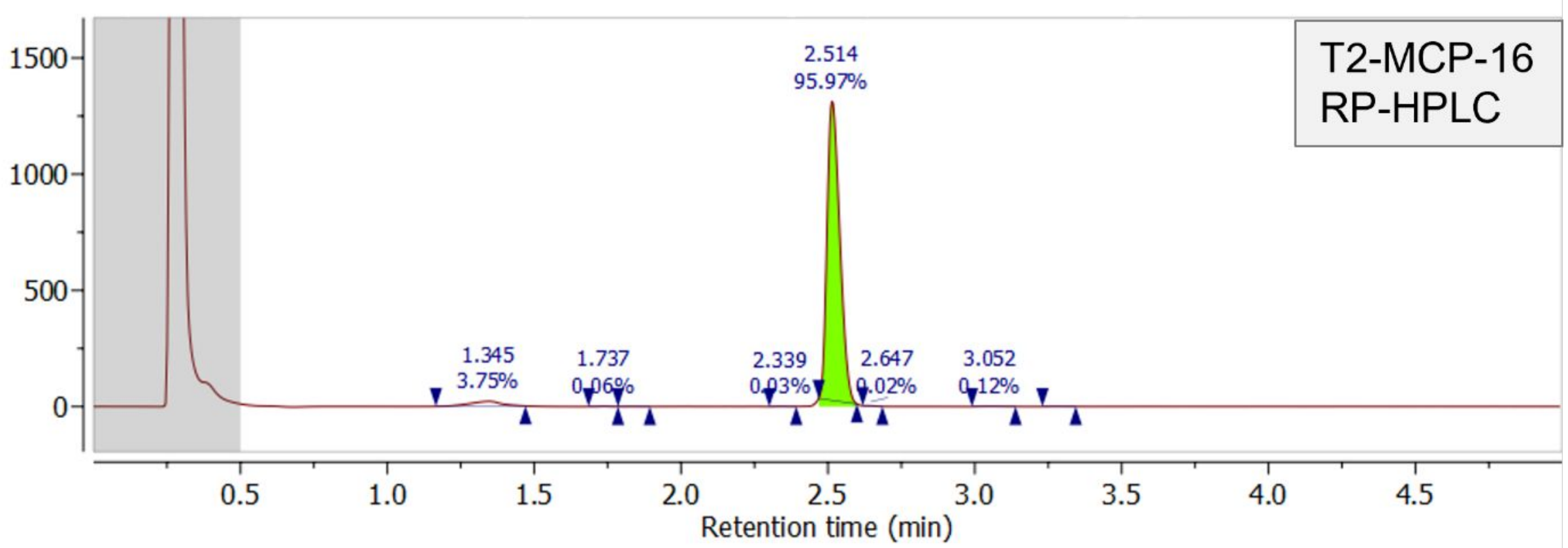
T2-MCP-16
RP-HPLC
2.514
95.97%
1.345
3.75%
1.737
0.06%
2.339
0.03%
2.647
0.02%
3.052
0.12%
Retention time (min)

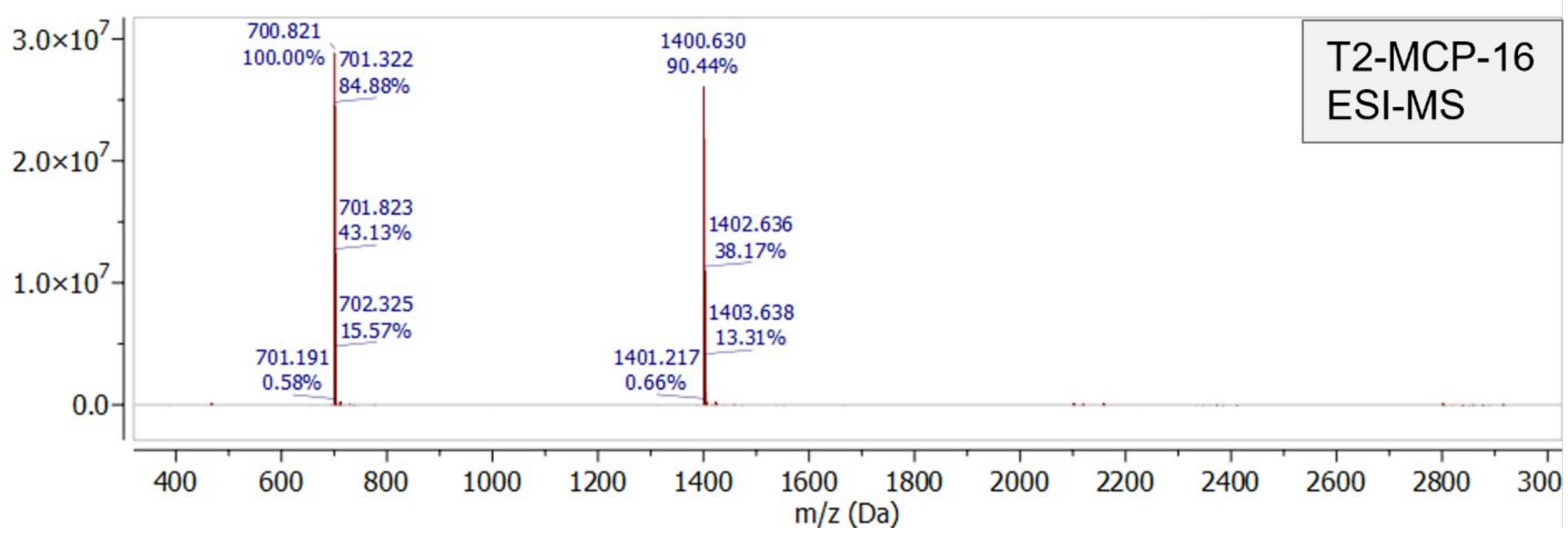
T2-MCP-16
ESI-MS
700.821
100.00%
701.322
84.88%
1400.630
90.44%
701.823
43.13%
1402.636
38.17%
702.325
15.57%
1403.638
13.31%
701.191
0.58%
1401.217
0.66%
m/z (Da)

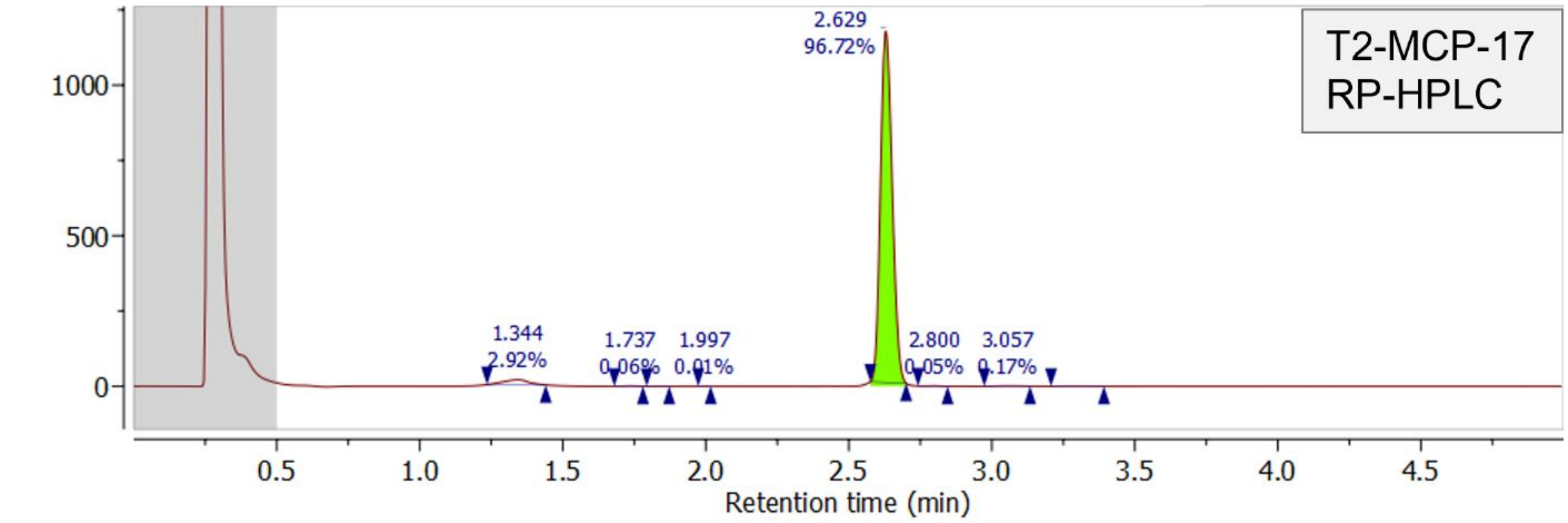
T2-MCP-17
RP-HPLC
2.629
96.72%
1.344
2.92%
1.737
0.06%
1.997
0.01%
2.800
0.05%
3.057
0.17%
1000
500
0
0.5
1.0
1.5
2.0
2.5
3.0
3.5
4.0
4.5
Retention time (min)

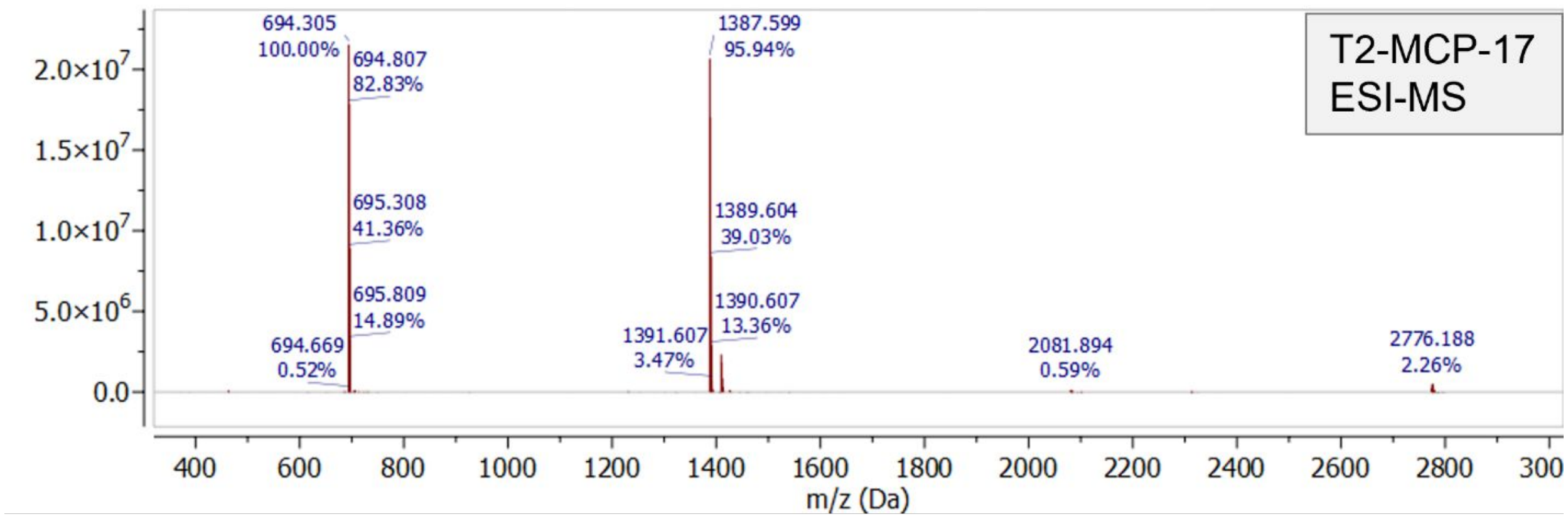
T2-MCP-17
ESI-MS
694.305
100.00%
694.807
82.83%
695.308
41.36%
695.809
14.89%
694.669
0.52%
1387.599
95.94%
1389.604
39.03%
1390.607
13.36%
1391.607
3.47%
2081.894
0.59%
2776.188
2.26%
2.0×10^7
1.5×10^7
1.0×10^7
5.0×10^6
0.0
400
600
800
1000
1200
1400
1600
1800
2000
2200
2400
2600
2800
300
m/z (Da)

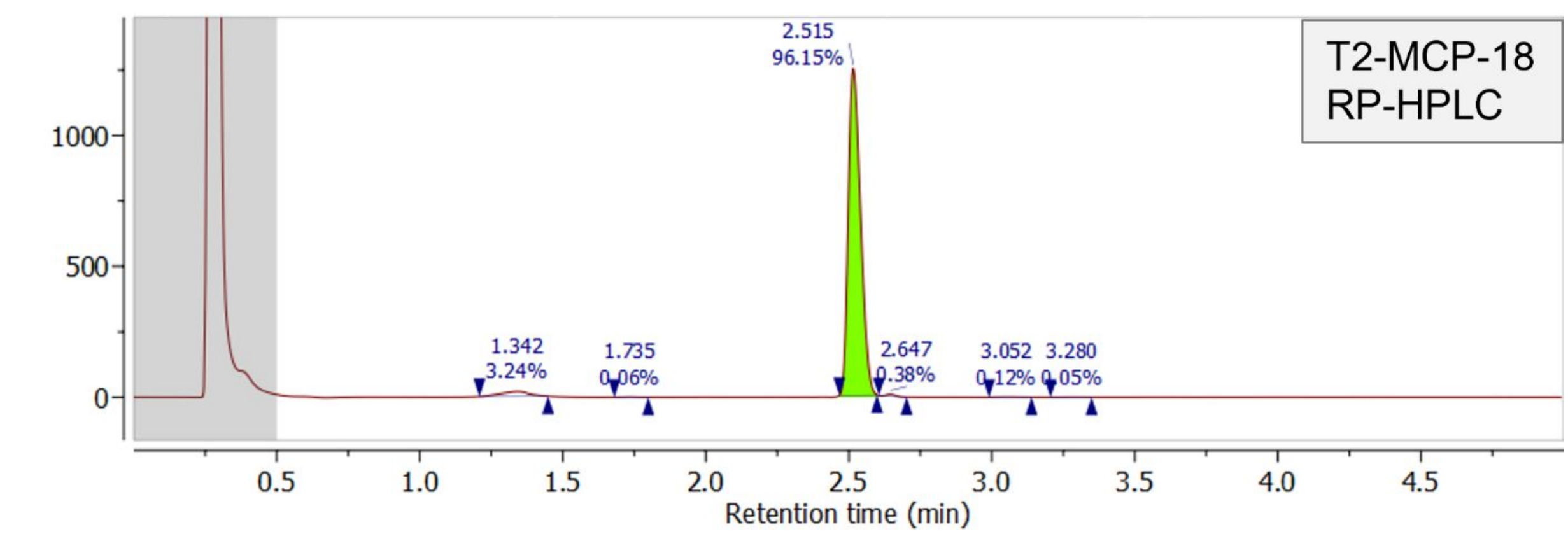
T2-MCP-18
RP-HPLC
2.515
96.15%
1.342
3.24%
1.735
0.06%
2.647
0.38%
3.052
0.12%
3.280
0.05%
1000
500
0
0.5
1.0
1.5
2.0
2.5
3.0
3.5
4.0
4.5
Retention time (min)

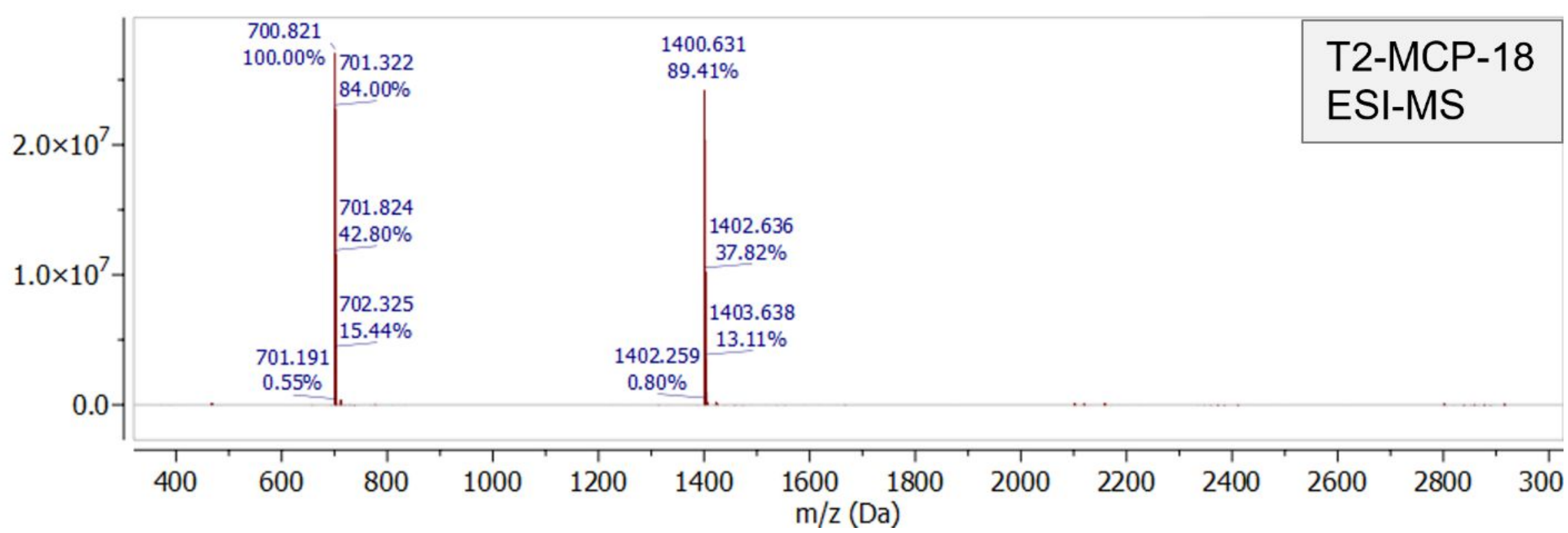
T2-MCP-18
ESI-MS
700.821
100.00%
701.322
84.00%
701.824
42.80%
702.325
15.44%
701.191
0.55%
1400.631
89.41%
1402.636
37.82%
1403.638
13.11%
1402.259
0.80%
2.0×10^7
1.0×10^7
0.0
400
600
800
1000
1200
1400
1600
1800
2000
2200
2400
2600
2800
300
m/z (Da)

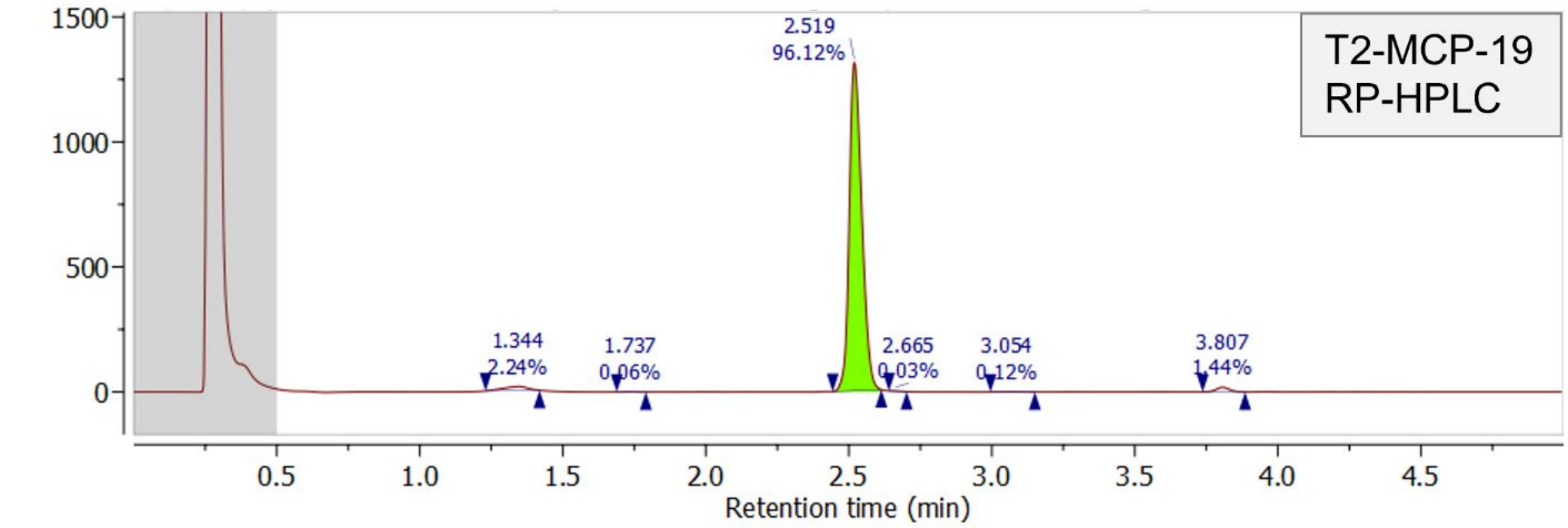
T2-MCP-19
RP-HPLC
2.519
96.12%
1.344
2.24%
1.737
0.06%
2.665
0.03%
3.054
0.12%
3.807
1.44%
Retention time (min)

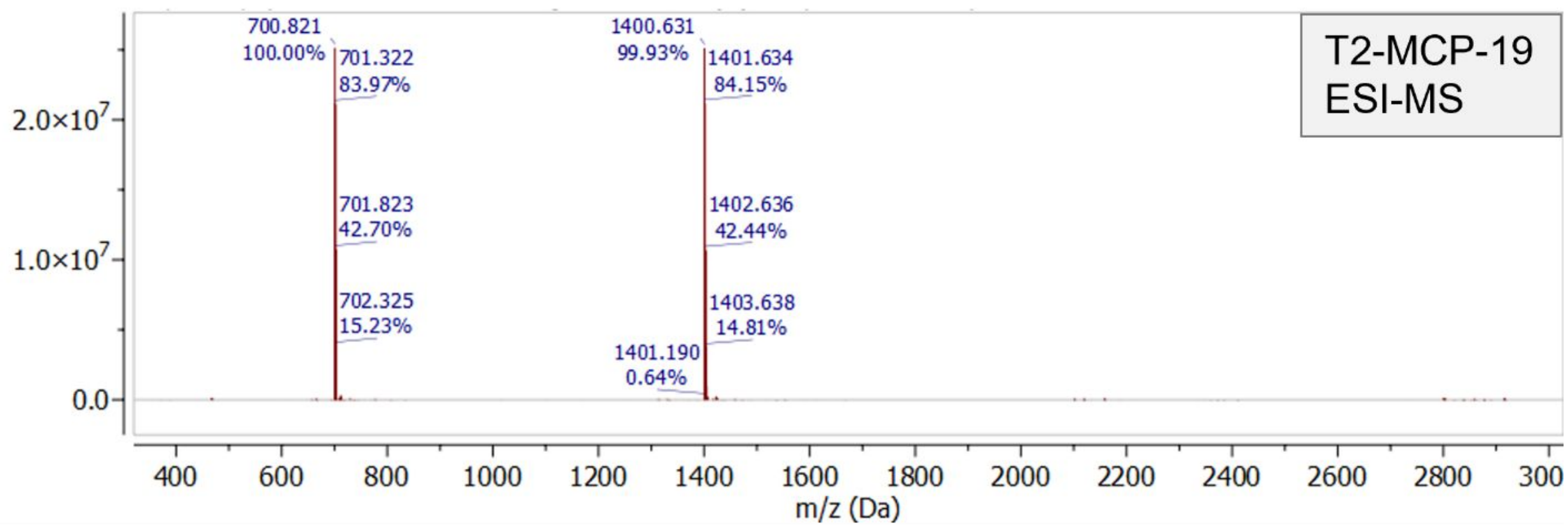
T2-MCP-19
ESI-MS
700.821
100.00%
701.322
83.97%
701.823
42.70%
702.325
15.23%
1400.631
99.93%
1401.634
84.15%
1402.636
42.44%
1403.638
14.81%
1401.190
0.64%
m/z (Da)

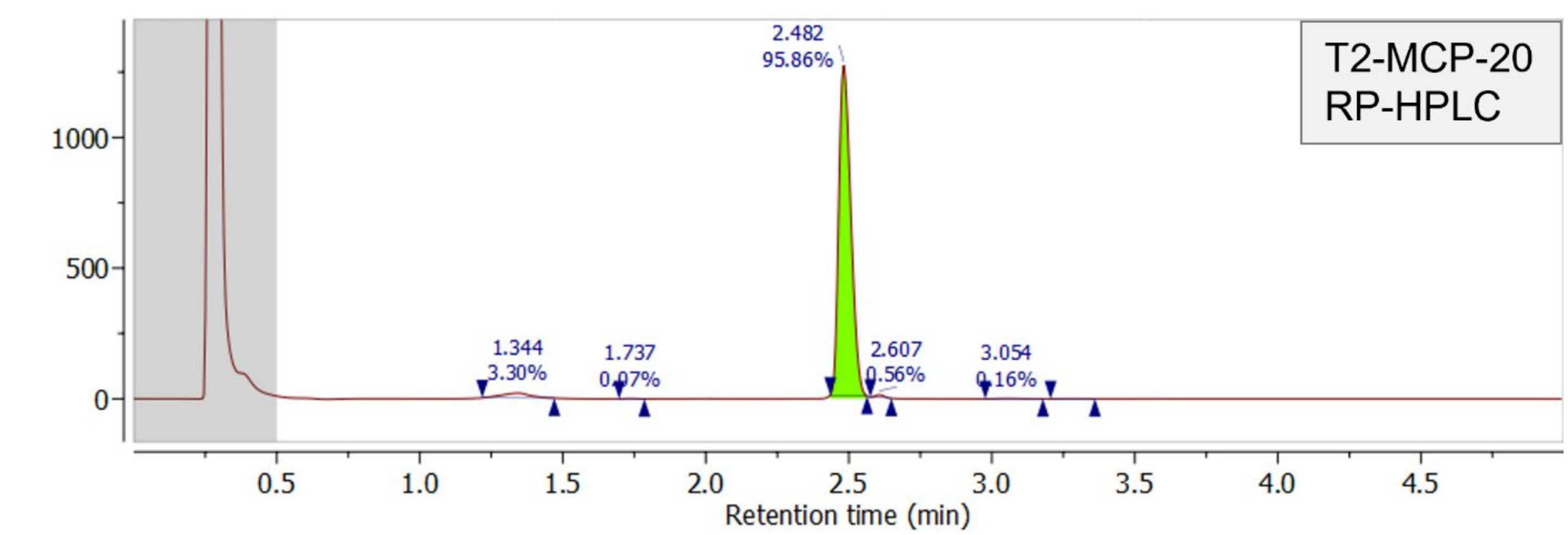
T2-MCP-20
RP-HPLC
2.482
95.86%
1.344
3.30%
1.737
0.07%
2.607
0.56%
3.054
0.16%
Retention time (min)

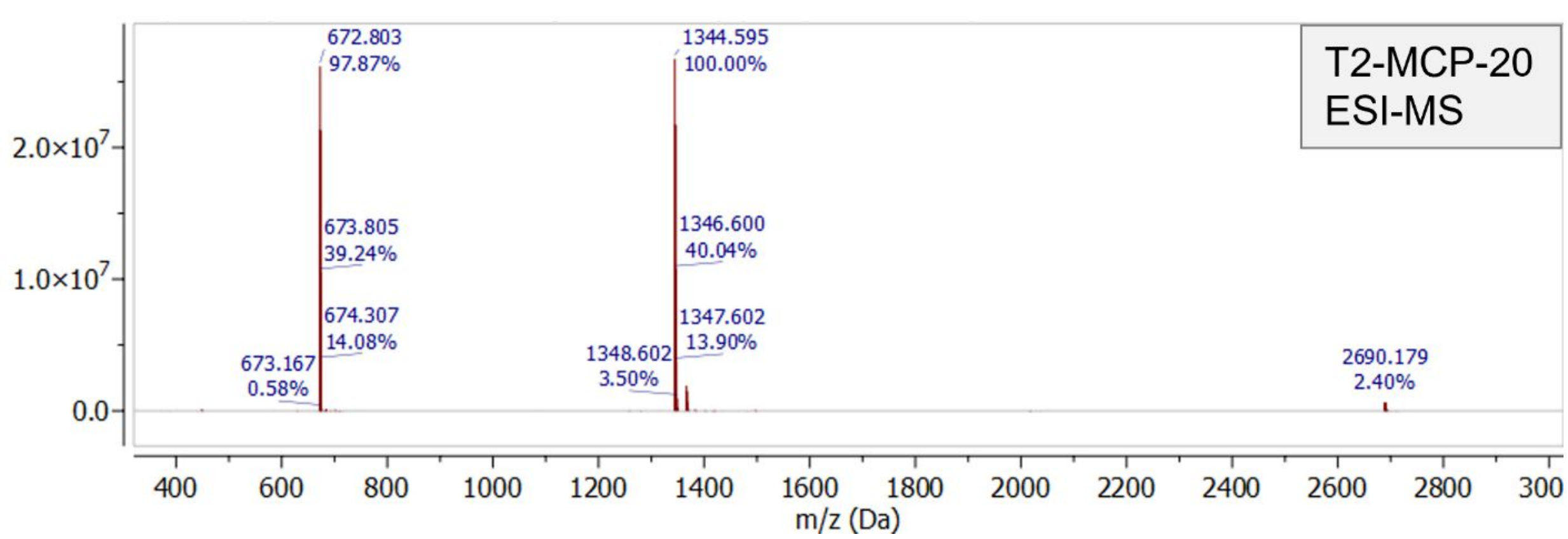
T2-MCP-20
ESI-MS
672.803
97.87%
673.805
39.24%
674.307
14.08%
673.167
0.58%
1344.595
100.00%
1346.600
40.04%
1347.602
13.90%
1348.602
3.50%
2690.179
2.40%
m/z (Da)

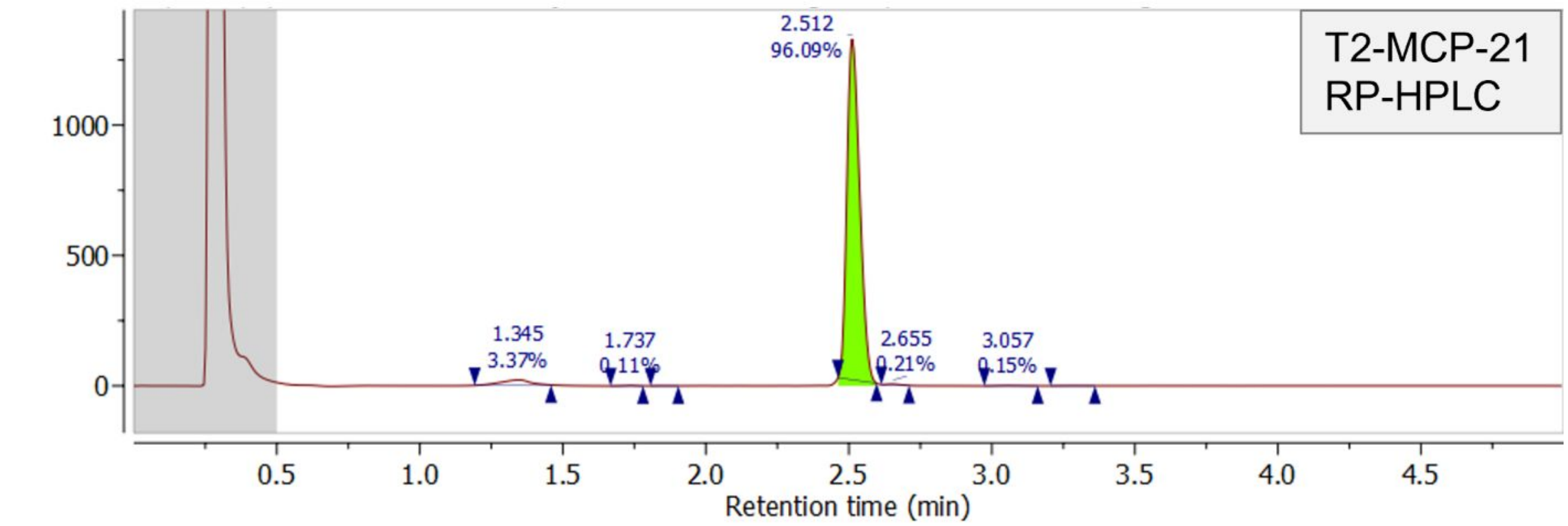

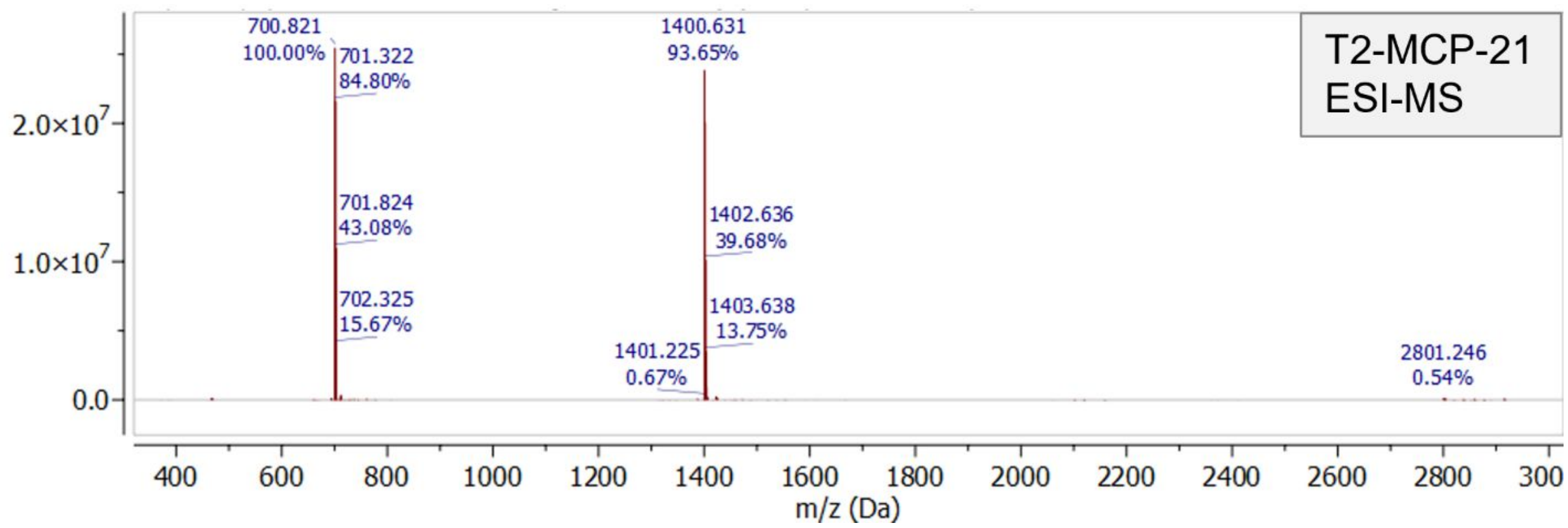

Figure S13. RP-HPLC and ESI-MS characterization of synthesized macrocyclic peptides. Representative RP-HPLC chromatograms (top panels) and ESI-MS spectra (bottom panels) for all macrocyclic peptides investigated in this study. Each chromatogram and spectrum and labeled with their respective peptide. RP-HPLC analysis was used to assess peptide purity, while ESI-MS confirmed the molecular identity of each peptide. Observed masses were consistent with the expected molecular weights of the synthesized compounds (see Table S3). Analytical LC-MS was performed on an Agilent 1260 Infinity II Liquid Chromatograph coupled to an Agilent 6230 TOF MS (0.7 mL min–1 flow rate, gradient elution from 15 to 85% ACN over 4 min) using an Agilent PLRP-S polymeric reversed-phase analytical column (50 mm × 2.1 mm ID, Part No. PL1912-1502) to verify compound purity. All final compounds achieved a minimum purity of 95% (Table S3).

Table S1. Frequency across the selection rounds of mRNA-display derived MCPs against recombinant TMPRSS2. Frequencies are reported as percentages of the total number of reads for each respective round. A value of *0* indicates complete absence of the MCP in a given selection round.

Library	MCP	Frequency in selection round				
		1	2	3	4	5
1	T2-MCP-01	0.01%	0.01%	2.93%	15.69%	23.93%
	T2-MCP-02	0.01%	0.01%	1.76%	9.31%	9.59%
	T2-MCP-03	0.00%	0.00%	0.73%	3.49%	3.88%
2	T2-MCP-04	0.00%	0.00%	0.17%	5.04%	25.81%
	T2-MCP-05	*0*	0.00%	0.04%	0.52%	1.21%
	T2-MCP-06	*0*	0.00%	0.04%	0.72%	1.78%
	T2-MCP-07	*0*	*0*	0.05%	0.42%	1.09%
3	T2-MCP-08	*0*	0.00%	0.04%	0.41%	2.63%
	T2-MCP-09	*0*	0.00%	0.03%	0.35%	2.98%
	T2-MCP-10	*0*	*0*	0.01%	0.07%	0.62%
4	T2-MCP-11	0.00%	0.00%	0.01%	0.32%	8.96%
	T2-MCP-12	0.00%	*0*	0.00%	0.02%	0.30%
	T2-MCP-13	0.00%	*0*	0.00%	0.09%	2.31%

Table S2. Inhibitory activities of selected MCPs against pericellular TMPRSS2 using VeroE6 cells transiently expressing human TMPRSS2. IC_{50} values shown are the average ± SD (n = 3–4 independent experiments).

**MCP**	**IC_{50} (nM)**
**T2-MCP-06**	99 ± 20
**T2-MCP-09**	9.8 ± 2.6
**T2-MCP-19**	1.6 ± 0.6

Table S3. The calculated peptide mass (M + H^+ calcd.) and the experimentally observed peptide mass (M + H^+ found) for each MCP synthesized. The peptide purity as determined by RP-HPLC is shown.

MCP	Peptide formula	M+H^+ calcd.	M+H^+ found	LC assay%
T2-MCP-01	C54H87N21O17S	1334.638	1334.605	97.12
T2-MCP-02	C70H96N20O20S	1569.690	1569.659	95.67
T2-MCP-03	C83H122N24O18S	1775.916	1775.882	95.68
T2-MCP-04	C103H124N22O21S	2037.911	2037.869	97.37
T2-MCP-05	C76H95N15O19S	1554.672	1554.642	97.29
T2-MCP-06	C78H106N18O15S	1567.788	1567.758	95.84
T2-MCP-07	C85H111N23O16S	1742.837	1742.803	96.86
T2-MCP-08	C75H96N16O17S	1525.693	1525.664	97.15
T2-MCP-09	C67H87N17O14S	1386.641	1386.615	96.74
T2-MCP-10	C69H93N19O16S	1476.684	1476.656	96.38
T2-MCP-11	C78H111N19O22S	1698.795	1698.763	97.00
T2-MCP-12	C78H110N20O16S	1615.820	1615.790	96.22
T2-MCP-13	C75H120N24O19S	1693.896	1693.864	95.41
T2-MCP-14	C67H87N17O14S	1386.641	1386.616	95.70
T2-MCP-15	C68H89N17O14S	1400.657	1400.631	95.81
T2-MCP-16	C68H89N17O14S	1400.657	1400.630	95.97
T2-MCP-17	C67H86N16O15S	1387.625	1387.599	96.72
T2-MCP-18	C68H89N17O14S	1400.657	1400.631	96.15
T2-MCP-19	C68H89N17O14S	1400.657	1400.631	96.12
T2-MCP-20	C66H85N15O14S	1344.619	1344.595	95.86
T2-MCP-21	C68H89N17O14S	1400.657	1400.631	96.09
T2-MCP-09-GKK	C81H114N22O17S	1699.853	1699.821	95.48
[R10A]T2-MCP-09-GKK	C78H107N19O17S	1614.789	1614.760	95.77
